# Novel Peptide-Based Fluorescent Probe for Simultaneous Sensing of Chymotrypsin and Hydrogen Peroxide

**DOI:** 10.1021/acsomega.4c00303

**Published:** 2024-04-05

**Authors:** David Milićević, Jan Hlaváč

**Affiliations:** Department of Organic Chemistry, Faculty of Science, Palacký University Olomouc, 17. Listopadu 12, 771 46 Olomouc, Czech Republic

## Abstract

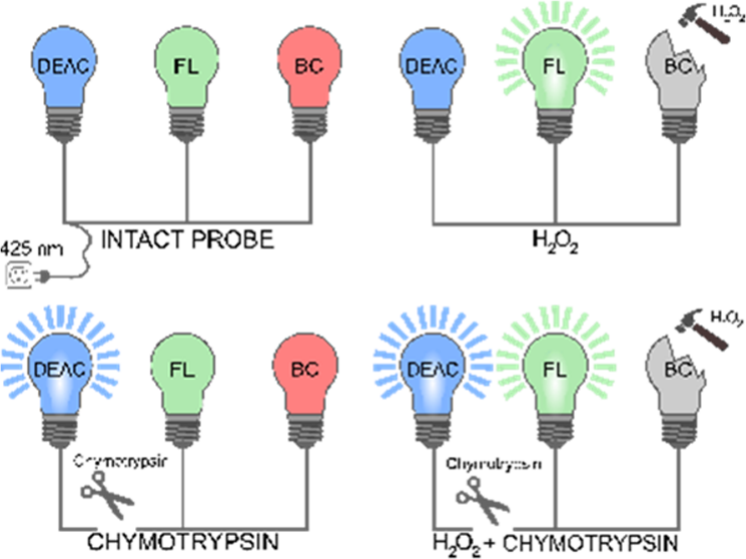

The developed multifunctional fluorescent probe enables the simultaneous detection of chymotrypsin as a model protease and hydrogen peroxide as a representative of reactive oxygen species (ROS) in biologically relevant concentration ranges. The chymotrypsin sensing is based on the cleavage of its selectively recognizable peptide sequence and the consequent disruption of FRET between coumarin (DEAC) and fluorescein (FL). Analogously, the presence of hydrogen peroxide causes the gradual degradation of the H_2_O_2_-labile benzopyrylium-coumarin (BC) dye. Considering the fluorescence emission responses of individual chymotrypsin-peroxide probe-attached fluorophores after their excitation at 425 nm, the sole presence of either chymotrypsin (50–1000 ng/mL) or hydrogen peroxide (10–200 μM) in a sample could be unambiguously confirmed or refuted. In addition, reliable simultaneous detection and approximate quantification of both studied species in the concentration ranges of 100–1000 ng/mL and 20–200 μM for chymotrypsin and H_2_O_2_, respectively, could be performed as well. The obtained results are summarized and visualized in the graphical models.

## Introduction

Reactive oxygen species (ROS) are enzymatic reaction byproducts with regulatory functions in many cellular processes such as metabolism, proliferation, osteoblastic differentiation, and immunity.^1,2^ However, in the case of elevated ROS levels, oxidative stress is induced in cells,^3^ resulting in cell organelle damage and subsequent cell death. One of the main representatives of reactive oxygen species is hydrogen peroxide (H_2_O_2_), whose intracellular concentration can vary by a few orders of magnitude, ranging from ∼10 nM (growth and proliferation) to ∼100 μM (apoptosis).^4^ Due to its general toxicity against the vast majority of cell types, H_2_O_2_ is also known as the most common apoptosis inducer.^5^ Because of its versatile role as a potent biomarker on a cellular level, the feasibility of its accurate and prompt screening in biological systems is of essential importance.^6^

Taking into account programmed cell death, apoptosis, caused by oxidative stress, we cannot overlook the fact that the involvements of hydrogen peroxide and proteases are tightly intertwined.^7^ Although the scientists may not always uniformly agree on, e.g., which initiator caspases are responsible for the activation of executioner caspases,^8^ there is no doubt that H_2_O_2_-induced apoptosis^9^ proceeds through a caspase activation pathway.^10,11^ Apart from the cysteine aspartic proteases, a direct connection between the serine proteases and hydrogen peroxide has been established as well. While some serine proteases such as, e.g., trypsin or neutrophil elastase, are cytotoxic and proven to increase ROS levels including H_2_O_2_ in cells,^12^ others, such as α-chymotrypsin, were found to be capable of suppressing oxidative stress and inflammation processes, consequently exhibiting protective potential against sepsis, and alleviating the damage to the kidneys, liver, and lungs.^13^

During the last couple of decades, numerous systems for the sole detection of ROS including hydrogen peroxide^14−18^ or various enzymes^19−21^ have been reported, among which fluorescence-based sensors are heavily represented. Because of their sensitivity, prompt responsiveness, and accuracy, they have been found to be indispensable in bioimaging^22^ and real-time sensing of numerous chemical,^23^ biochemical,^24^ and biological^25^ species. While the majority of these probes are capable of solely single-analyte screening, their multifunctional counterparts appear in significantly lower numbers. Considering the recent trends in medicinal and biological scientific areas associated with the growing interest in a deeper and more comprehensive elucidation of complex and frequently interconnected biochemical processes, the application of multifunctional sensors with the capability of simultaneous multicomponent detection is clearly irreplaceable in some cases.

While quite a few studies describing multiplex protease sensing,^26−30^ synchronous enzyme and pH monitoring,^31−34^ and detection of hydrogen peroxide in combination with other nonenzymatic species^35−38^ exist, to the best of our knowledge, there are only two known reports dealing with the simultaneous enzyme and hydrogen peroxide screening. Peng et al. developed a dual-locked NIR fluorescence-based sensor for selective marking of melanoma cells, applying peroxide tyrosinase cascade activation of a fluorescence-inactive methylene blue borate derivative.^39^ While this probe was proven effective for in vivo tumor visualization, it possesses some limitations from the perspective of individual H_2_O_2_ and tyrosinase detection, as only the copresence of both analytes results in the desired fluorescence response. Another dual-imaging system capable of in vivo protease and hydrogen peroxide detection operates on the principle of an “AND” molecular logic gate, where simultaneous dismembering of the H_2_O_2_ probe and caspase-8 peptide sequence occurs, resulting in two corresponding cleaved fragments that are subsequently combined in situ to form bioluminescent firefly luciferin.^40^ Although the authors highlighted the versatility of the developed system including its ability to monitor both studied analytes in a synchronous as well as individual manner with the addition of a complementary luciferin-forming precursor, the potential drawback might lie in the fact that cellular uptake, in-cell distribution, and specific organelle accumulation of both molecular entities could considerably differ case by case.

To address the scarcity of such kinds of systems for simultaneous hydrogen peroxide and protease screening, we constructed a single-molecule fluorescently tagged peptide probe for reliable aforementioned analyte screening in biologically relevant concentration ranges. The developed sensor can be employed for both individual and synchronous detection of H_2_O_2_ and chymotrypsin in a sample, applying a fluorophore decomposition and FRET principles, respectively.

## Methods and Materials

Both the CP probe (chymotrypsin-peroxide probe) and C probe (chymotrypsin probe) were synthesized on a solid support, applying Fmoc-based solid-phase synthesis. The chemicals and solvents used in this study were obtained from available commercial sources and were used without additional purification. Benzopyrylium-coumarin (BC)^41^ and 7-(diethylamino)coumarin-3-carboxylic acid (DEAC)^42^ fluorescent dyes were synthesized as described in the corresponding literature sources.

### Synthesis

Into a 20 mL plastic syringe (B. Braun Melsungen AG, Melsungen, Germany) furnished with a plastic sintered filter (Torviq, Tucson, AZ), polystyrene Wang resin (500 mg; 0.9 mmol/g, AAPPTec, Louisville, KY) was weighed. Subsequently, it was prewashed with dichloromethane (3 × 10 mL) and subjected to multistep synthesis on a microplate shaker (Thermo Fisher Scientific, Waltham, MA), using suitable reagents in appropriate concentrations. After completion of individual transformation steps, a solid support was manually washed with dimethyl sulfoxide (DMSO; 5 × 10 mL) and/or dimethylformamide (DMF; 10 × 10 mL) and dichloromethane (DCM; 10 × 10 mL), while an analytical amount of resin was transferred into an Eppendorf tube and treated with 50% trifluoroacetic acid (TFA) in DCM (V/V) for approximately 15 min. After the evaporation of volatiles under a stream of nitrogen, the resulting dry or sticky residue was diluted with 50% acetonitrile in ultrapure water (V/V), filtered through a nylon syringe filter (0.2 μm, J.T. Baker, Avantor, Pennsylvania), and analyzed on a UHPLC chromatograph (Acquity) with a photodiode array detector and a single quadrupole mass spectrometer (Waters, Borehamwood, U.K.). The analyses were performed on a reversed-phase C-18 XSelect HSS T3 2.5 μm XP (50 × 3.0 mm) column (Waters, Borehamwood, U.K.). A solution of ammonium acetate (10 mM) in ultrapure water and acetonitrile (gradient 20–80% during the first 4.5 min) was utilized. The chromatograms and corresponding mass spectra are presented in the Supporting Information (SI–Figures S1–S9).

### Purification and Storage

After finalization of the multistep synthesis, the resin-immobilized CP probe was first properly washed with dichloromethane (10 × 10 mL) and methanol (10 × 10 mL), subsequently dried under a stream of nitrogen, and finally cleaved from the solid support using 50% trifluoroacetic acid in DCM (3 × 10 mL; 3 × 15 min). After the removal of volatiles under a stream of nitrogen, the resulting dark-blue sticky residue was diluted with 70% acetonitrile in ultrapure water (V/V) and purified on a semiprep HPLC column (YMC-Actus Pro C18, 100 mm × 20 mm I.D. S-5 μm, 12 nm, Dinslaken, Germany), employing a gradient of 40–55% (16 min) and then 55–80% (2 min) acetonitrile in 0.1% TFA in ultrapure water (V/V). After in vacuo (Buchi R-215 Rotavapor, Marshall Scientific, Flawil, Switzerland) concentration of combined fractions and subsequent freeze drying (Scanvac Coolsafe Freeze-Dryer, LaboGene, Lillerød, Denmark) for 48 h, the obtained dry residue was again dissolved in 70% acetonitrile in ultrapure water (V/V) and subjected to the second round of purification on a semiprep HPLC column. This time, a gradient of 35–60% (12 min) acetonitrile in 10 mM ammonium acetate in ultrapure water was used. The concentrated water solution of the CP probe was then freeze-dried for 72 h, gaining a dark-blue solid, which was properly aliquoted into Eppendorf safe-lock tubes (1.5 mL, Hamburg, Germany) and finally stored at −80 °C in a deep freezer (Arctiko, Esbjerg Kommune, Denmark).

The isolation process of the C probe was very similar to the one described for the CP probe. It differed only in the application of different mobile phases during the two-step purification process. In this case, gradients of 30–65% (11 min) acetonitrile in 10 mM ammonium acetate in ultrapure water for the first and 35–70% (9 min) acetonitrile in 0.1% TFA in ultrapure water (V/V) for the second purification round were used. After freeze drying, a dark-orange solid (C probe) was aliquoted and stored at −80 °C.

### Application

Hydrogen peroxide (30%, AnalaR NORMAPUR) was ordered from VWR International (France), and α-chymotrypsin (bovine pancreas, type II, ≥40 units per mg of protein) was obtained from Sigma-Aldrich (Germany) in the form of lyophilized white powder. Before usage, the protease was reconstituted in 1 mM HCl in ultrapure water, aliquoted in Eppendorf safe-lock tubes, and finally stored at −80 °C in a deep freezer (Arctiko, Esbjerg Kommune, Denmark). All assays were performed in 0.1 M Tris buffer (Roche Diagnostics GmbH, Mannheim, Germany) at pH = 8.0, adjusted by gradual dropwise addition of concentrated NaOH aqueous solution.

To a fluorescent probe in the form of powder was added an appropriate amount of DMSO to obtain a solution with a target concentration of 1 mM. The corresponding aliquots of 10 μL were then placed in Eppendorf safe-lock tubes, stored at −80 °C, and used within the next few days.

To an Eppendorf tube with a 10 μL solution of 1 mM fluorescent probe in DMSO, Tris buffer (970 μL, pH = 8.0) was added. The obtained solution was transferred to a covered plastic fluorimeter cuvette (Merck, Italy), placed into a temperature-controlled cuvette holder inside a fluorescence spectrometer (Cary Eclipse, Agilent Technologies, Santa Carla, CA), and preheated to 37 °C for 15 min. After an emission spectrum for the time 0 min was measured, 10 μL of 1 mM HCl with or without protease in an appropriate concentration and 10 μL of water solution with or without hydrogen peroxide in a suitable amount were added, and the obtained sample in the covered fluorimeter cuvette was incubated at 37 °C for the time period of 45 min. The emission spectra upon uniform excitation with 425 nm were measured every five min at the times of 5, 10, 15, 20, 25, 30, 35, 40, and 45 min.

### CP Probe Selectivity Assays

Into a plastic fluorimeter cuvette (Merck, Italy), 10 μL of 1 mM CP probe in DMSO was placed. Then, Tris buffer (980 μL, pH = 8.0) and 10 μL of 10 mM solution of appropriate ROS (H_2_O_2_, ^•^OH, *t*BuOOH, ^•^O*t*Bu, and ClO^–^) in ultrapure water were added. For blank samples, 10 μL of ultrapure water was applied instead of the ROS solution. In the case of superoxide anions, Tris buffer (988 μL, pH = 8.0) and 10 μL of 10 mM solution of O_2_^•–^ in DMSO were added to 2 μL of 5 mM CP probe in DMSO. As a source of superoxide anions (O_2_^•–^), KO_2_ was utilized. Hydroxyl (^•^OH) and *tert*-butoxy (^•^O*t*Bu) radicals were generated by the reaction of 10 mM FeBr_2_ with hydrogen peroxide and *tert*-butyl hydroperoxide, respectively. All stock solutions of ROS were freshly prepared and administered shortly after their preparation. The experiments took place in covered fluorimeter cuvettes in an incubator (Heratherm, Thermo Fisher Scientific) at 37 °C. After the completion of 45 min of incubation, a cuvette with a sample was immediately transferred to a fluorescence spectrometer (Cary Eclipse, Agilent Technologies, Santa Carla, CA) equipped with a temperature-controlled cuvette holder (37 °C), and a fluorescence emission response upon excitation with 425 nm was measured. For the purpose of evaluation of CP probe selectivity toward hydrogen peroxide, emission maxima at 477 nm (DEAC) and 529 nm (FL) were taken into consideration.

### Spectral Properties

The CP probe was obtained in the form of a dark-blue powder, which turned into a green-yellowish solution when dissolved in 1% (V/V) solution of DMSO in 0.1 M Tris buffer (pH = 8.0). The fluorescence emission maximum readouts for the CP probe were set to 477, 529, and 722 nm for coumarin (DEAC), fluorescein (FL), and benzopyrylium-coumarin (BC), respectively, upon uniform excitation at 425 nm. Employing the same excitation wavelength of 425 nm, the emission responses for DEAC and FL at 477 and 529 nm, respectively, were taken into consideration also in the case of the C probe. The quantum yields of the CP probe-attached fluorescent dyes were determined, employing the general formula Φ_X_ = Φ_ref_ × (∇_X_/∇_ref_) × (η_X_^2^/η_ref_^2^), where Φ represents the fluorescence quantum yield, ∇ denotes a gradient of integrated fluorescence intensity vs absorbance, and η indicates the solvent refractive index. Fluorescein (0.1 M NaOH), Rhodamine 6G (water), and Rhodamine B (water) were used as references. The corresponding numerical data are collected in the Supporting Information (SI–Table S1).

## Results and Discussion

### Development and Spectral Characteristics of the CP Probe

**Scheme 1:**
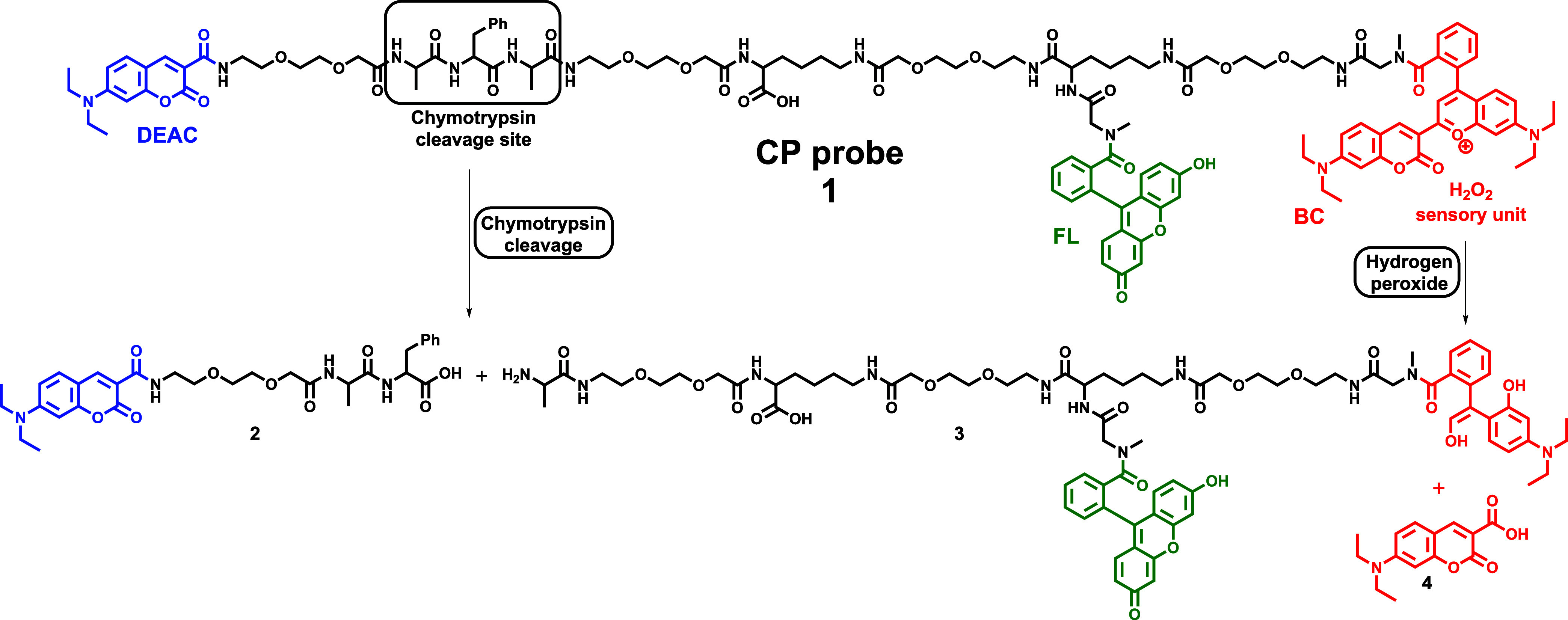
Dual-Purpose CP Probe for Simultaneous Chymotrypsin and Hydrogen Peroxide Screening and Its Mode of Action

The Fmoc-based solid-phase synthetic approach was applied for the preparation of the three-fluorophore CP probe consisting of two main parts: a selectively cleavable Ala-Phe-Ala peptide sequence surrounded by a FRET pair of fluorophores for chymotrypsin sensing and a fluorescent unit responsible for hydrogen peroxide detection (Scheme 1). While chymotrypsin is often considered a readily available and easy-to-handle model serine protease, it has also been advantageously employed in various medical applications as an antioxidant and anti-inflammatory agent.^43^ On the other hand, among the reactive oxygen species, hydrogen peroxide is known as one of the most common representatives. Taking into account these facts, the developed CP probe, capable of detecting both of the aforementioned species in biologically relevant concentration ranges, could be used as a model system or a real sensor with a practical application. To improve the efficiency of protease cleavage as well as the solubility of the designed sensor in aqueous media, four poly(ethylene glycol)-based (PEG) spacers were incorporated into the CP probe’s structure. Next, the binding sites for individual fluorophores were also taken into consideration. While DEAC was bound directly to the primary amino group of a poly(ethylene glycol)-based (PEG) spacer (Scheme 1; blue structure), FL and BC were attached to sarcosine through the tertiary amide (Scheme 1; green and red structures), to avoid the well-known formation of the corresponding spirolactam frameworks and consequent partial loss of their fluorescence properties. Finally, the utilization of Fmoc-Lys(Mtt)–OH with a selectively removable 4-methyltrityl (Mtt) lysine side-chain protecting group in the three-armed synthetic approach enabled two-sided prolongation of the immobilized peptide backbone as well as its decoration with FL in the middle of the amino acid sequence.

**Figure 1:**
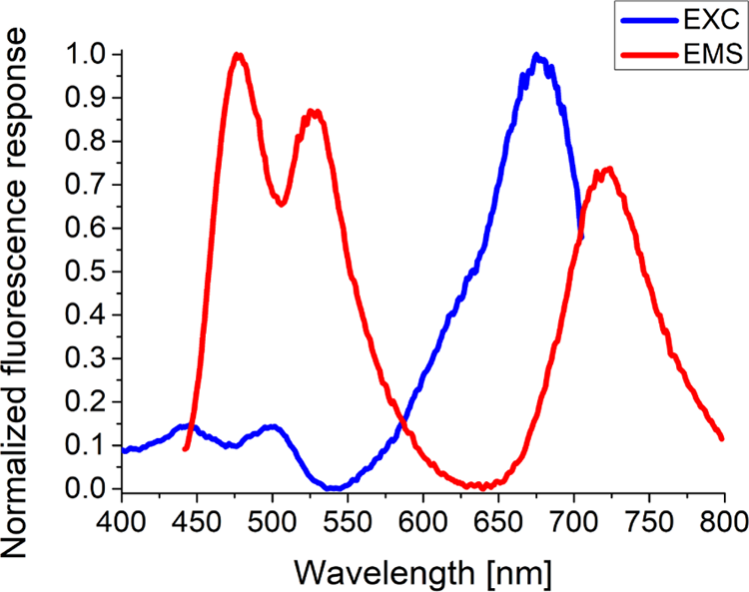
Normalized fluorescence excitation (λ_EMS._ = 722 nm) and emission (λ_EXC._ = 425 nm) profiles of the CP probe in 1% DMSO (V/V*)* in 0.1 M Tris buffer (pH = 8.0) at 37 °C.

Taking into account the excitation and emission profiles of the CP probe, three clearly expressed signals belonging to the three fluorophores attached to the peptide backbone could be identified in both cases (Figure 1). In comparison with the spectral maxima of the intact free fluorophores DEAC (λ_EMS._ = 471 nm; λ_EXC._ = 410 nm), FL (λ_EMS._ = 517 nm; λ_EXC._ = 497 nm), and BC (λ_EMS._ = 691 nm; λ_EXC._ = 657 nm) in 1% DMSO (V/V) in 0.1 M Tris buffer (pH = 8.0) (SI–Figures S18–S20), negligible to notable bathochromic shifts of 6, 12, and 31 nm for the CP probe-bound DEAC, FL, and BC, respectively, were observed.

### Application of the CP Probe

**Figure 2:**
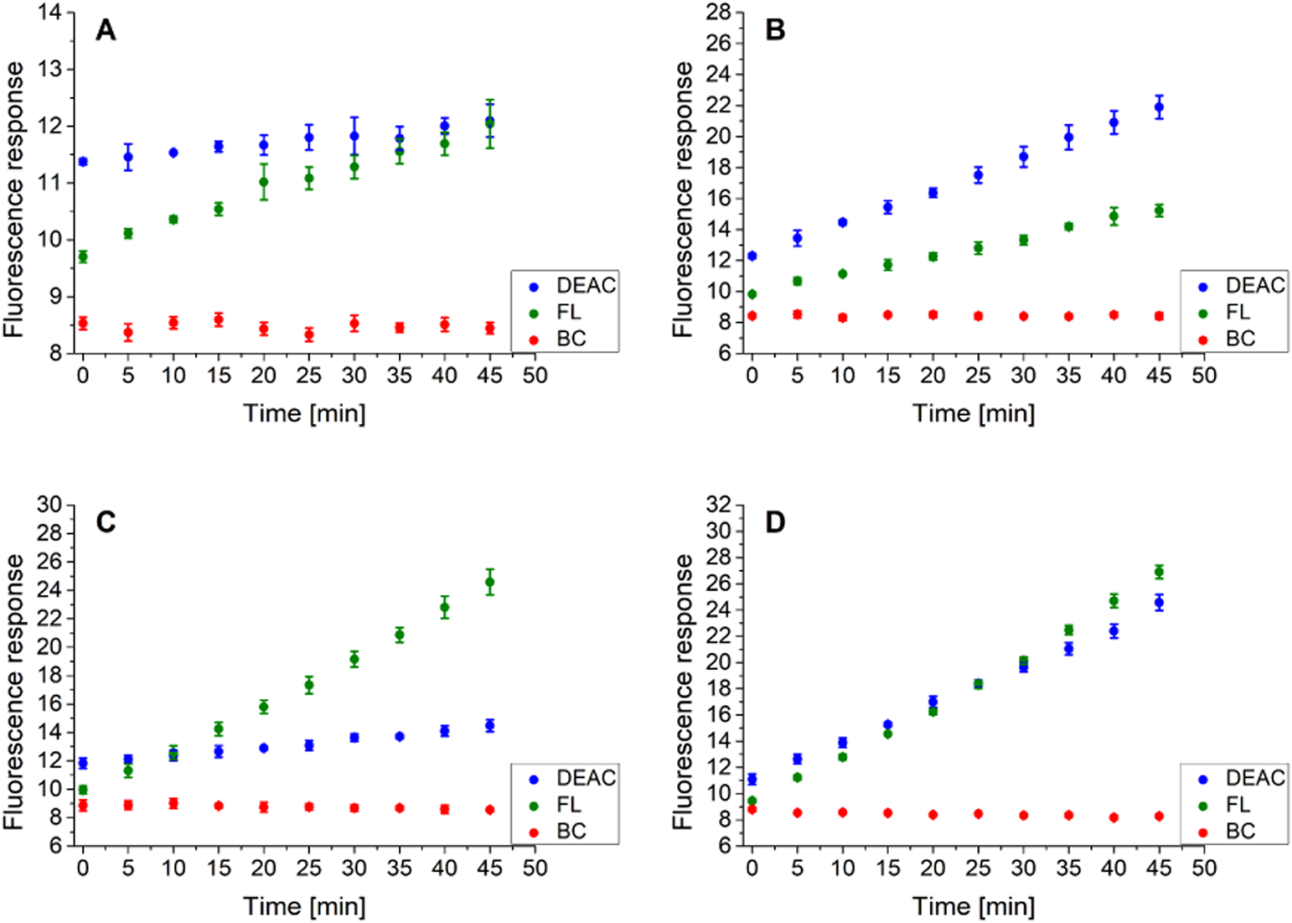
Fluorescence emission response of the intact CP probe within the time in the absence of both analytes (A), in the presence of chymotrypsin (200 ng/mL) (B), in the presence of hydrogen peroxide (50 μM) (C), and in the copresence of chymotrypsin (200 ng/mL) and hydrogen peroxide (50 μM) (D), upon excitation with 425 nm. The measurements were performed in three independent parallels in Tris buffer (pH = 8.0) at 37 °C. The average values and standard deviations are graphically presented, while the corresponding numerical data are collected in the Supporting Information (SI–Tables S2, S5, S10, and S18).

In the first step, the fluorescence emission maxima for all three fluorophores attached to the CP probe were monitored throughout the time. As can be seen in Figure 2A (SI–Table S2), slight rises of 6% in DEAC and 24% in FL emission intensities upon excitation with 425 nm were detected for blank samples during the time period of 45 min. In the presence of chymotrypsin, the cleavage between the *N*-terminus of alanine and the *C*-terminus of phenylalanine took place, causing the disruption of FRET between DEAC and FL and thus resulting in the notable increase in the DEAC emission response (Figure 2B and SI–Table S5). Analogously, the increase in DEAC intensity accompanied by the decrease in the BC emission signal was expected to take place in the presence of hydrogen peroxide, as during the H_2_O_2_-labile BC dye decomposition, a new molecule of DEAC is generated (Scheme 1). Instead, a predominant rise in the FL fluorescence signal upon excitation with 425 nm was detected (Figure 2C and SI–Table S10). Bearing in mind that the emission signal of FL and the excitation signal of BC are overlapped between 550 and 625 nm (Figure 1), we surmise that the fluorescence energy transfer might exist also between FL and BC and not only between DEAC and FL. Presumably, when the BC moiety is decomposed by hydrogen peroxide, the donor–acceptor energy transfer between FL and BC might be disabled, resulting in the enhancement of the FL emission signal. Finally, in the copresence of both studied analytes in a mixture, substantial increases in both DEAC and FL emission responses were clearly perceptible (Figure 2D and SI–Table S18).

### Principles of H_2_O_2_ Detection

**Scheme 2:**
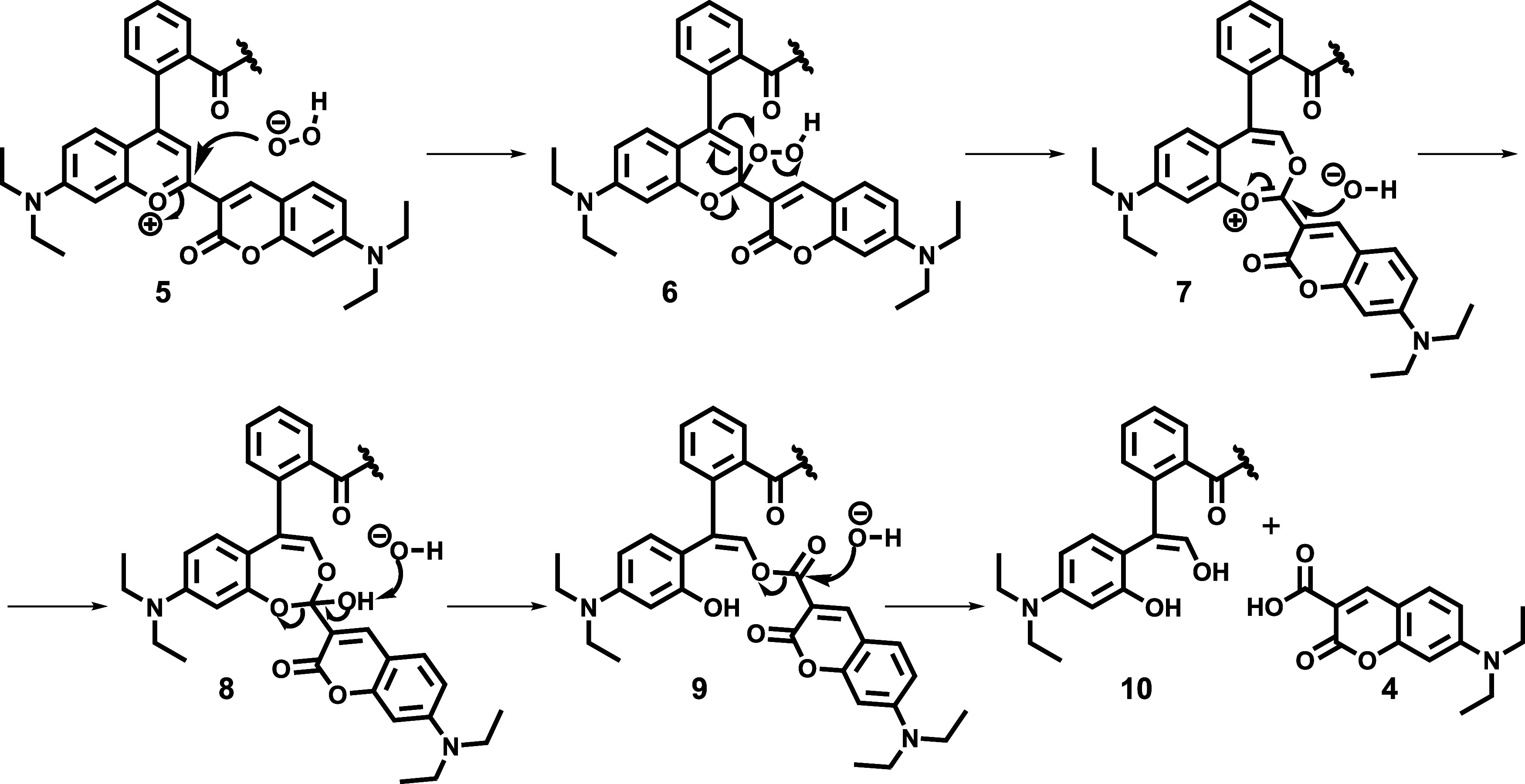
Slightly Modified Proposed Mechanism^41^ of H_2_O_2_-Induced BC Moiety Decomposition

A slightly modified putative mechanism of H_2_O_2_-induced BC framework degradation is proposed in Scheme 2. According to the inspiration from the literature,^41^ Baeyer–Villiger oxidation is probably initiated by the nucleophilic attack of hydroperoxyl species to the carbonyl group with the more positively charged oxygen atom (compound **5**), to gain the corresponding 2*H*-chromen derivative **6**. In the next step, the oxidative rearrangement takes place, yielding dioxepinium compound **7**, which then presumably undergoes another nucleophilic attack to the carbonyl group with the more positively charged oxygen atom, to provide hydroxybenzodioxepinyl intermediate **8**. After its tautomerism-induced ring-opening and subsequent hydrolysis of the resulting vinyl ester **9**, pertinent diol **10** and a molecule of DEAC (**4**) are formed.

**Scheme 3:**
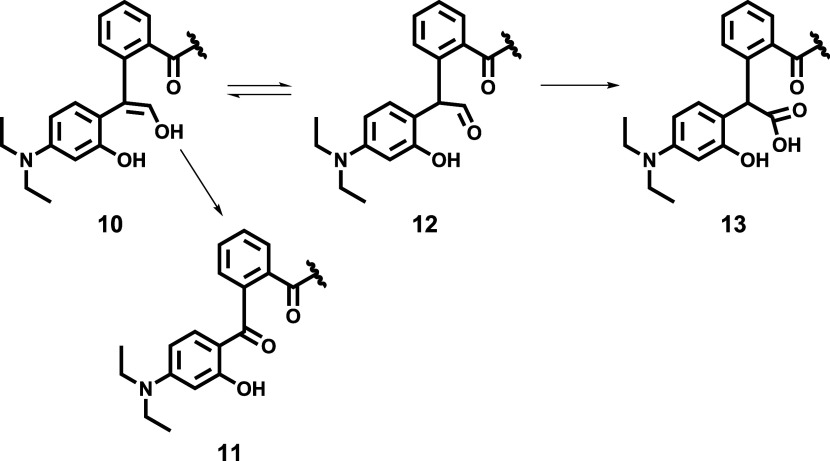
Transformation of Compound **10** into the Corresponding Ketone (**11**), Aldehyde (**12**), and Carboxylic Acid (**13**) Derivatives The corresponding LC-MS data are collected in the Supporting Information (SI–Figure S13).

Based on the LC-MS studies and as depicted in Scheme 3, compound **10** is then probably subjected to oxidative cleavage transformation to give the appropriate ketone derivative **11** (SI–Figure S13; Compound **I**) as well as to vinyl alcohol–acetaldehyde tautomerism to yield the corresponding aldehyde **12** (SI–Figure S13; Compound **III**), which is subsequently further oxidized to the suitable carboxylic acid **13** (SI–Figure S13; Compound **II**).

From the perspective of the CP probe spectral properties and its behavior in the presence of H_2_O_2_, the existence of intermediate **9** (SI–Figure S13; Compound IV) might be of crucial importance. We believed that the second FRET channel between ester-bound–DEAC-bearing intermediate **9** and FL could be temporarily established during the H_2_O_2_-induced BC dye decomposition process. Consequently, upon excitation with 425 nm, two molecules of DEAC might contribute to FL excitation, resulting in its enhanced emission response.

**Figure 3:**
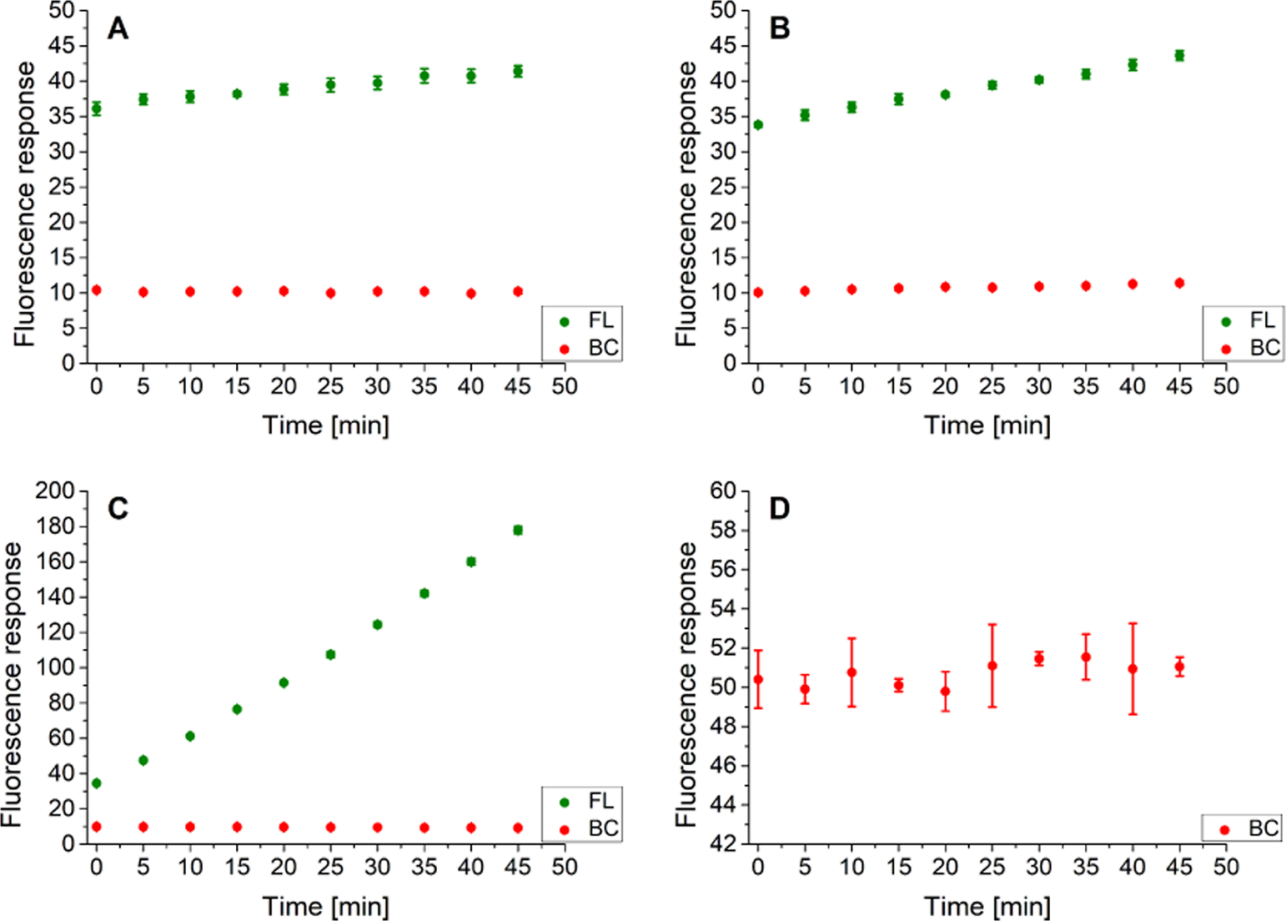
Fluorescence emission response of the CP probe within the time in the absence of both analytes (A), in the presence of chymotrypsin (1 μg/mL) (B), and in the presence of hydrogen peroxide (200 μM) (C), upon excitation with 500 nm. Fluorescence emission response of the CP probe within the time, in the presence of hydrogen peroxide (200 μM) (D), upon excitation with 680 nm. The measurements were performed in three independent parallels in Tris buffer (pH = 8.0) at 37 °C. The average values and standard deviations are graphically presented, while the corresponding numerical data are collected in the Supporting Information (SI–Tables S30–S33).

To evaluate this assumption, potential FL signal enrichment caused by FRET between DEAC and FL was avoided by employing an excitation wavelength of 500 nm, while the corresponding FL emission response at 529 nm was taken into consideration. The measurements were performed in the presence of the maximal studied concentrations of chymotrypsin (1 μg/mL) and hydrogen peroxide (200 μM) as well as in the absence of both analytes. In the case of blank samples (Figure 3A) and those subjected to chymotrypsin cleavage (Figure 3B), only insignificant rises in FL emission intensities of 15% (SI–Table S30) and 29% (SI–Table S31), respectively, during 45 min were detected. On the other hand, a 45 min incubation of the CP probe with H_2_O_2_ (200 μM) resulted in a more than fivefold increase in the FL response (Figure 3C and SI–Table S32). This indicates that the potential establishment of the two-channel FRET during the BC dye degradation and consequent generation of an additional ester-bound DEAC molecule (intermediate **9**) cannot be the only trigger of the predominant FL signal enhancement, but it could be accompanied by, e.g., BC-induced FL quenching. Despite the fact that the BC gradual decomposition in the presence of hydrogen peroxide was distinctly confirmed (SI–Figure S13), only negligible decreases of 13% (λ_EXC._ = 425 nm; SI–Table S12) and 7% (λ_EXC._ = 500 nm; SI–Table S32) in the BC emission response (λ_EMS._ = 722 nm) were detected during the 45 min CP probe treatment with H_2_O_2_ (200 μM). Finally, no worth-mentioning changes in the BC signal (λ_EMS._ = 722 nm) within the time were observed, when the excitation wavelength of 680 nm was applied (Figure 3D). These observations are in fairly good compliance with the literature,^41^ where the changes in BC fluorescence response upon treatment with H_2_O_2_ were found to be significantly less pronounced for the red channel than for the green channel.

### Application of the C Probe

**Scheme 4:**
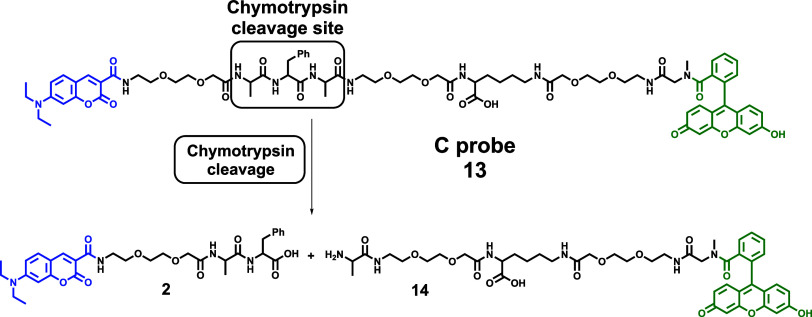
Single-Purpose C Probe for Chymotrypsin Screening and Its Mode of Action

To unambiguously establish whether the DEAC-FL segment of the CP probe could be somehow affected by hydrogen peroxide or the presence of the BC framework is mandatory from the perspective of H_2_O_2_ sensing, a single-purpose C probe (Scheme 4) consisting of a chymotrypsin-cleavable sequence equipped with a FRET pair of DEAC and FL was synthesized and applied to both target species screening. For the purpose of clear and unambiguous evaluation of its characteristics, 10-fold aforementioned maximal studied concentrations of chymotrypsin (10 μg/mL) and hydrogen peroxide (2 mM) were used.

**Figure 4:**
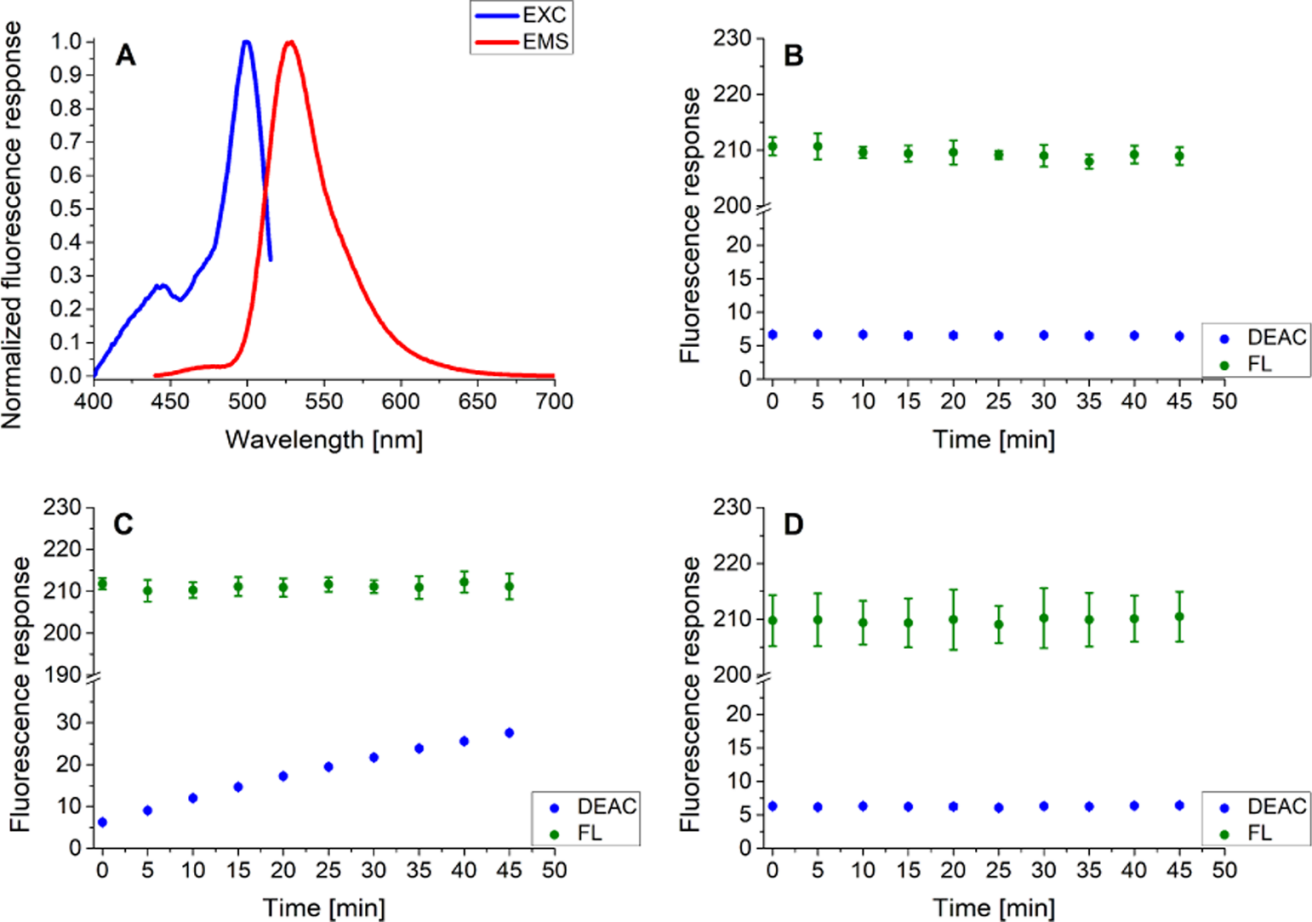
Normalized fluorescence excitation (λ_EMS._ = 530 nm) and emission (λ_EXC._ = 425 nm) profiles of the C probe in 1% DMSO (V/V) in 0.1 M Tris buffer (pH = 8.0) at 37 °C (A). Fluorescence emission response of the C probe within the time, in the absence of both analytes (B), in the presence of chymotrypsin (10 μg/mL) (C), and in the presence of hydrogen peroxide (2 mM) (D). The measurements (B–D) were performed in three independent parallels in Tris buffer (pH = 8.0) at 37 °C. The average values and standard deviations are graphically presented, while the corresponding numerical data are collected in the Supporting Information (SI–Tables S34–S36).

The normalized excitation and emission profiles of the C probe in 1% DMSO (V/V) in 0.1 M Tris buffer (pH = 8.0) are presented in Figure 4A. As can be seen in Figure 4B (SI–Table S34), in the absence of both target analytes, the fluorescence emission responses of both C probe-attached fluorophores were found unchanged during the entire duration of the experiment. According to the expectations, the C probe was dismembered in two well-defined peptide fragments when treated with chymotrypsin (SI–Figure S14), resulting in a significant rise in the DEAC fluorescence signal (Figure 4C and SI–Table S35). In contrast, the intact LC-MS chromatogram of the C probe after its incubation with hydrogen peroxide (SI-Figure S15) as well as its stable fluorescence response within a time indicates its complete unresponsiveness to H_2_O_2_ (Figure 4D and SI–Table S36).

Considering all of the properties and functionalities discussed above of both synthesized sensors, we can rightly assume that the presence of BC in a probe importantly influences the FL emission response by reducing it more than 20 times (comparing SI–Tables S2 and S34). This phenomenon could be potentially caused by the contributions of a few events such as energy transfer between fluorophores, fluorescence quenching due to the dyes’ proximity, or differences in peptide folding directly related to the conformational entropy of a system. With the progressive H_2_O_2_-induced degradation of BC, the fluorescence response of FL is gradually revived, resulting in its predominant rise upon the incubation of the CP probe with hydrogen peroxide.

### Chymotrypsin and H_2_O_2_ Sensing with the CP Probe

By setting the length of incubation to 45 min and considering the average values as well as standard deviations of the CP probe-bound DEAC, FL, and BC emission maxima obtained upon excitation with 425 nm, the lowest detectable concentrations of 50 ng/mL and 10 μM for the sole presence of chymotrypsin and hydrogen peroxide, respectively, were experimentally established. Then, the same procedure was applied for the simultaneous screening of both studied species in a mixture. In this case, the determined detection limits were found to be two times higher: 100 ng/mL for chymotrypsin and 20 μM for H_2_O_2_.

**Figure 5:**
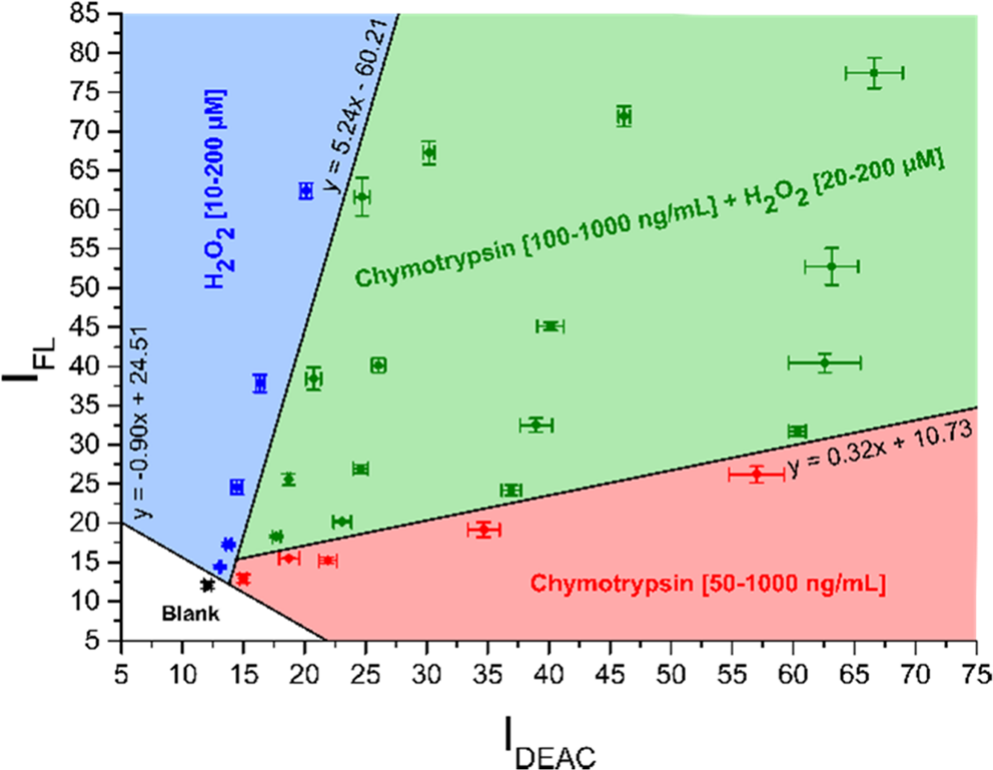
Graphical “detection model” visualizes the sole presence of chymotrypsin (red) and the sole presence of hydrogen peroxide (blue) as well as the coexistence of both species (green) in the defined concentration ranges. The blank measurements appear in the white area. The corresponding numerical data are collected in the Supporting Information (SI–Table S29).

For the purpose of both studied species detection visualizations, a graphical model was constructed, where only emission signals of DEAC and FL were taken into consideration, as solely negligible changes in BC emission intensity were detected throughout the entire sequence of the experiments. By plotting fluorescence emission responses of DEAC (*I*_DEAC_; λ_EMS._ = 477 nm) on the *X*-axis and FL (*I*_FL_; λ_EMS._ = 529 nm) on the *Y*-axis, the measured values were distributed in a two-dimensional space as presented in Figure 5. Then, the coordinate system was divided into four districts employing three linear lines with given equations and both coordinate axes. When neither protease nor H_2_O_2_ was present in a system, the resulting fluorescence responses after a 45 min assay appeared in the proximity of the blank point in the white area. The measurements in the blue sector specified the existence of only hydrogen peroxide in the defined concentrations, while those in the red region depicted the sole presence of chymotrypsin. Finally, the values in the green section resulted from the coexistence of both studied species in given concentration ranges.

**Figure 6:**
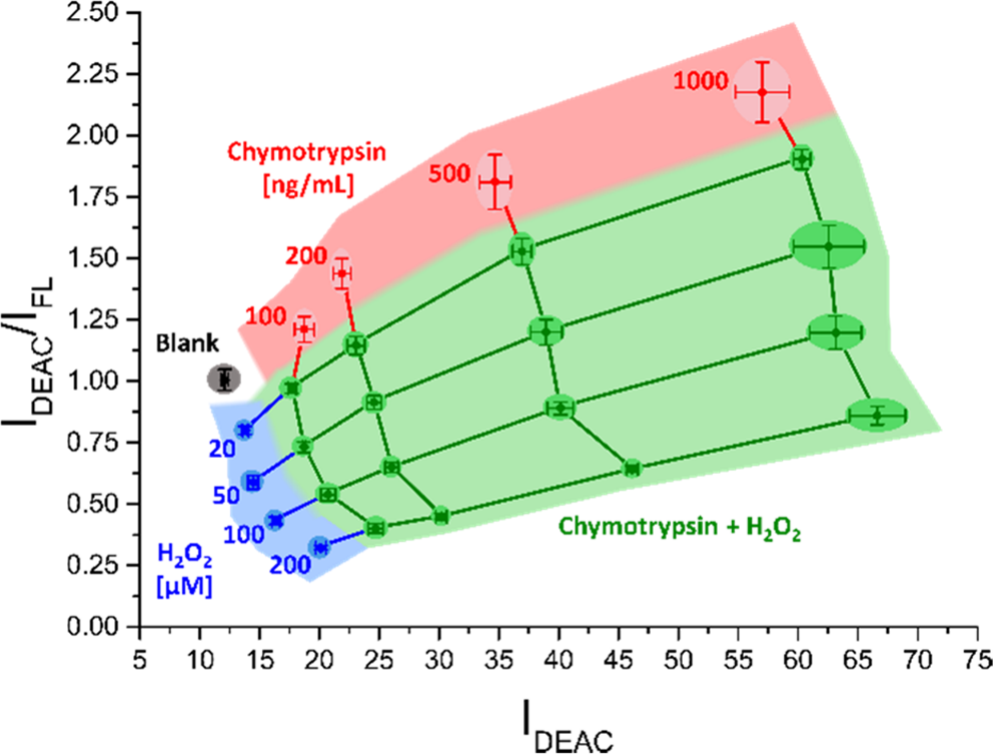
Graphical “determination model” enables approximate quantification of chymotrypsin (red), hydrogen peroxide (blue), and both species in a mixture (green) at various concentrations. The zones representing the same concentrations of individual species are interconnected with individual lines. The corresponding numerical data are collected in the Supporting Information (SI–Table S29).

After setting the rules for the unequivocal detection of protease and H_2_O_2_ in individual as well as combined manners, we focused on the quantification of the aforementioned analytes. Taking into account various combinations of fluorescence emission responses of CP probe-attached fluorophores measured after 45 min of incubation, the best results were obtained when DEAC/FL intensity ratios *I*_DEAC_/*I*_FL_ (λ_EMS._ = 477 nm/λ_EMS._ = 529 nm) were placed on the *Y*-axis. Considering the significantly smaller increase in the average DEAC emission response (6% rise in 45 min) in comparison with the FL one (24% rise in 45 min) for blank samples (Figure 2A and SI–Table S2), the absolute values of DEAC emission maximum *I*_DEAC_ (λ_EMS._ = 477 nm) were preferentially chosen and plotted on the *X*-axis. As can be seen in Figure 6, appropriate colorful fields that are color-matched with those in Figure 5 denote the presence of suitable analytes in a system. Inside particular larger areas, smaller zones strictly defined with the standard deviations of the measured average values could be found. When the zones representing the same combination of analyte concentrations are interconnected, the network enabling approximate quantification of chymotrypsin and hydrogen peroxide within the given concentration scopes is obtained (Figure 6). The accuracy of the graphical quantification tool could be potentially improved by the increased density of the network, which directly corresponds with the number and distribution of measured combinations of target species’ concentrations.

### Selectivity of the CP Probe toward H_2_O_2_

**Figure 7:**
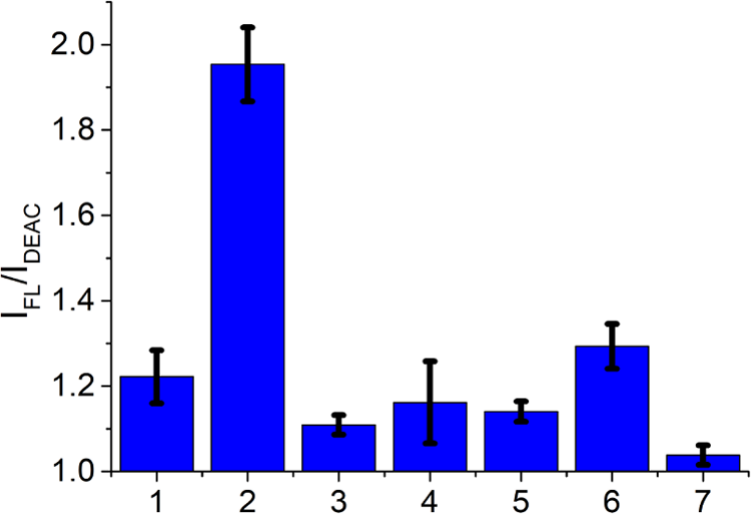
FL/DEAC ratio-based response of the CP probe for the blank (1), 100 μM H_2_O_2_ (2), 100 μM ^•^OH (3), 100 μM *t*BuOOH (4), 100 μM ^•^O*t*Bu (5), 100 μM O_2_^•–^ (6), and 100 μM ClO^–^ (7). The corresponding numerical data are collected in the Supporting Information (SI–Table S38).

To evaluate the selectivity of the synthesized sensor toward hydrogen peroxide, the CP probe was treated with various reactive oxygen species (ROS) in the concentration of 100 μM. Comparing the FL/DEAC emission response ratios measured after 45 min of incubation at 37 °C (Figure 7), the obtained average value for the samples subjected to hydrogen peroxide was found to be more than 50% higher than for the parallels treated with other ROS species and for blank controls. The acquired results are in good compliance with the reported selectivity study for the BC fluorophore.^41^

## Conclusions

To summarize, a three-fluorophore peptide-based chymotrypsin-peroxide probe, exploiting the principles of FRET between DEAC and FL for protease sensing, as well as gradual decomposition of the H_2_O_2_-labile BC unit for hydrogen peroxide detection, has been constructed and successfully applied in practice. Based on the emission responses of the CP probe-attached fluorophores, measured after 45 min of incubation in Tris buffer using a single excitation wavelength of 425 nm, potential individual or synchronous presence of both studied analytes in a system could be unambiguously detected in the defined biologically relevant concentration ranges. Furthermore, the developed network model enables the approximate quantification of protease and H_2_O_2_ concentrations, while its accuracy rises with the increasing density of the net. By prompt and simple modification of a selectively cleavable site, the obtained sensor can be efficiently adapted to any other enzyme species screening. According to needs or application requirements, the probe’s emission maximum wavelengths could be properly adjusted by the weighty selection among the large variety of fluorescent dyes available nowadays.

## Supporting Information Available

Synthetic scheme, LC-MS spectra, and fluorescence measurement data (PDF)
