# Supplementary material for: Novel Peptide-Based Fluorescent Probe for Simultaneous Sensing of Chymotrypsin and Hydrogen Peroxide
Source: ACS Omega. 2024 Apr 5;9(15):17481–90. doi: 10.1021/acsomega.4c00303 (PMC11024966; doi:10.1021/acsomega.4c00303)
Supplement: Supplementary file 1 — ao4c00303_si_001.pdf [file ao4c00303_si_001.pdf]

<u>Supporting Information</u>

Novel Peptide-Based Fluorescent Probe for Simultaneous Sensing of Chymotrypsin and Hydrogen Peroxide

David Milićević and Jan Hlaváč*

Department of Organic Chemistry, Faculty of Science, Palacký University Olomouc, 17. Listopadu 12, 771 46 Olomouc, Czech Republic.

*Corresponding author email: jan.hlavac@upol.cz

Table of Contents

1. Synthesis....S2

2. LC-MS analyses – Synthesis of CP probe....S3

3. LC-MS analyses – Intact CP and C probes....S12

4. LC-MS analyses – Application....S14

5. HRMS analyses....S24

6. Fluorescence analyses....S26

1. Synthesis

Scheme S1. Synthesis of CP probe (chymotrypsin-peroxide probe).

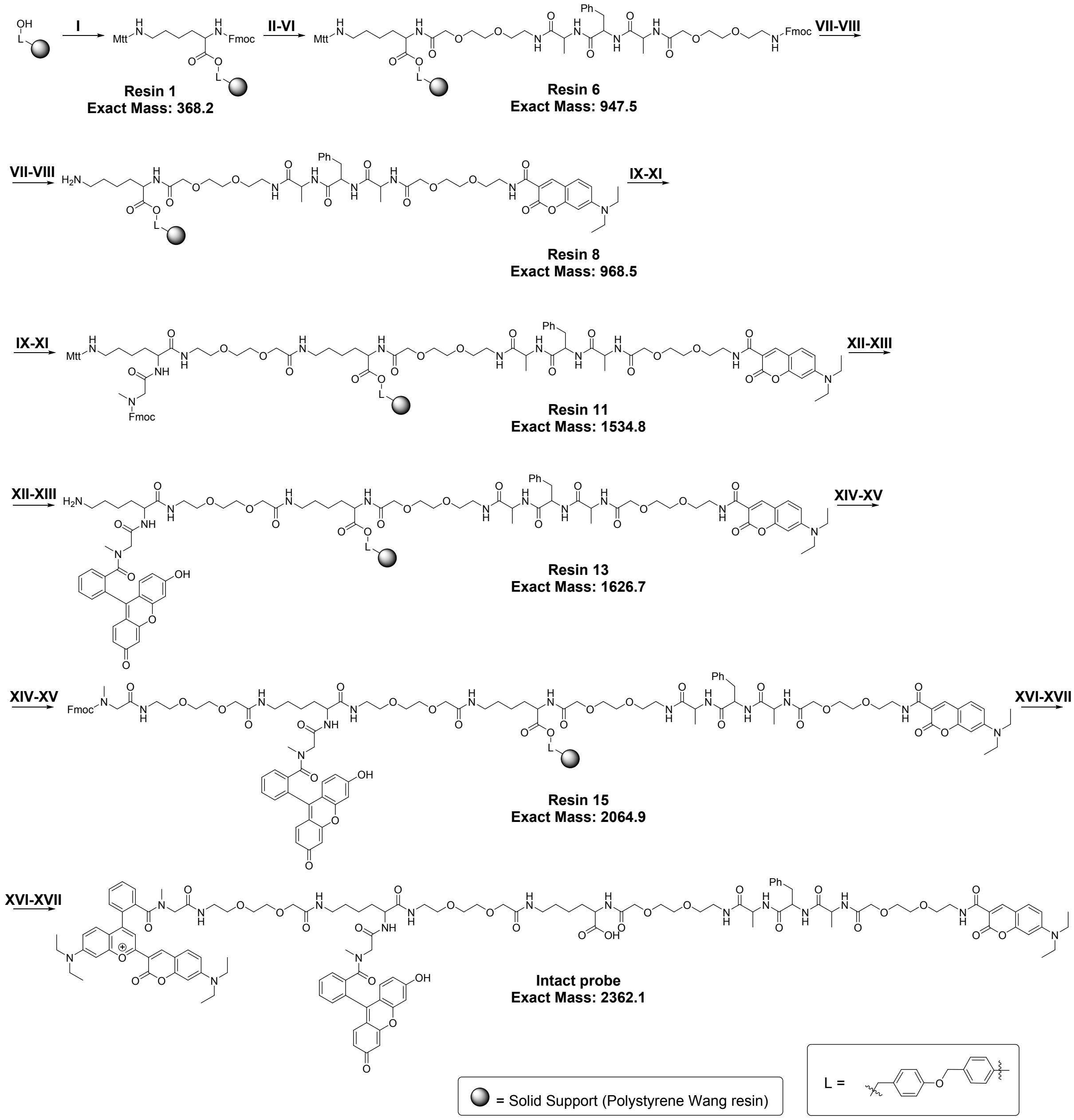

I. Fmoc-Lys(Mtt)-OH, HOBt, DMAP, DIC, DMF:DCM 1:1, rt, 16h; **II.** a.) 50% piperidine in DMF, rt, 30 min; b.) Fmoc-PEG, HOBt, DIC, DMF:DCM 1:1, rt, 2h; **III**. a.) 50% piperidine in DMF, rt, 30 min; b.) Fmoc-Ala-OH, HOBt, DIC, DMF:DCM 1:1, rt, 2h; **IV.** a.) 50% piperidine in DMF, rt, 30 min; b.) Fmoc-Phe-OH, HOBt, DIC, DMF:DCM 1:1, rt, 2h; **V.** a.) 50% piperidine in DMF, rt, 30 min; b.) Fmoc-Ala-OH, HOBt, DIC, DMF:DCM 1:1, rt, 2h; **VI.** a.) 50% piperidine in DMF, rt, 30 min; b.) Fmoc-PEG, HOBt, DIC, DMF:DCM 1:1, rt, 2h; **VII.** a.) 50% piperidine in DMF, rt, 30 min; b.) DEAC, HOBt, DMAP, DIC, DMF:DCM:DMSO 1:2:2, rt, 16h; **VIII.** DCE, TES, HFIP, TFE, 60 °C, 6h; **IX.** Fmoc-PEG, HOBt, DIC, DMF:DCM 1:1, rt, 3h; **X.** a.) 50% piperidine in DMF, rt, 30 min; b.) Fmoc-Lys(Mtt)-OH, HOBt, DIC, DMF:DCM 1:1, rt, 3h; **XI.** a.) 50% piperidine in DMF, rt, 30 min; b.) Fmoc-Sar-OH, HOBt, DIC, DMF:DCM 1:1, rt, 3h; **XII.** a.) 50% piperidine in DMF, rt, 30 min; b.) FL, HOBt, DMAP, DIC, DMF:DCM:DMSO 1:2:2, rt, 16h; **XIII.** DCE, TES, HFIP, TFE, 60 °C, 6h; **XIV.** Fmoc-PEG, HOBt, DIC, DMF:DCM 1:1, rt, 16h; **XV.** a.) 50% piperidine in DMF, rt, 30 min; b.) Fmoc-Sar-OH, HOBt, DIC, DMF:DCM 1:1, rt, 3h; **XVI.** a.) 50% piperidine in DMF, rt, 30 min; b.) BC, HOBt, DMAP, DIC, DMF:DCM:DMSO 1:2:2, rt, 16h; **XVII.** 50% TFA in DCM, rt, 45 min.

Exact molecular masses of resin-cleaved peptides are reported. 4-Methyltrityl (Mtt) protecting group is removed during the chemical cleavage with 50% TFA in DCM.

Synthesis of C probe (chymotrypsin probe)

The CP probe reaction steps **I–IX**, **XI** and **XII** (Scheme S1) were applied also for C probe preparation. The transformation step **X** (Scheme S1) was omitted, as Fmoc-Sar-OH was attached directly to the *N*-terminus of PEG.

2. LC-MS analyses – Synthesis of CP probe

Method: *Ammonium acetate (10 mM) in ultrapure water and acetonitrile (gradient 20–80% during the first 4.5 min)*
LC-MS column: *50 x 3.0 mm XSelect HSS T3 2.5 µm XP, Waters, Borehamwood, UK*

Resin 1

A solid support was prewashed with dichloromethane (3x 10 mL) and subsequently reacted with Fmoc-Lys(Mtt)-OH (2.0 mmol), HOBt (2.0 mmol), DMAP (0.5 mmol), and DIC (2.0 mmol) in DMF (5 mL) and DCM (5 mL). The reaction mixture was shaken for 16 hours at lab temperature. Then, a resin was washed with DMF (10x 10 mL) and DCM (10x 10 mL).

Figure S1. LC-MS analysis of chemically cleaved peptide from Resin 1 (Rt = 2.00 min), Mtt protecting group (Rt = 5.16 min).

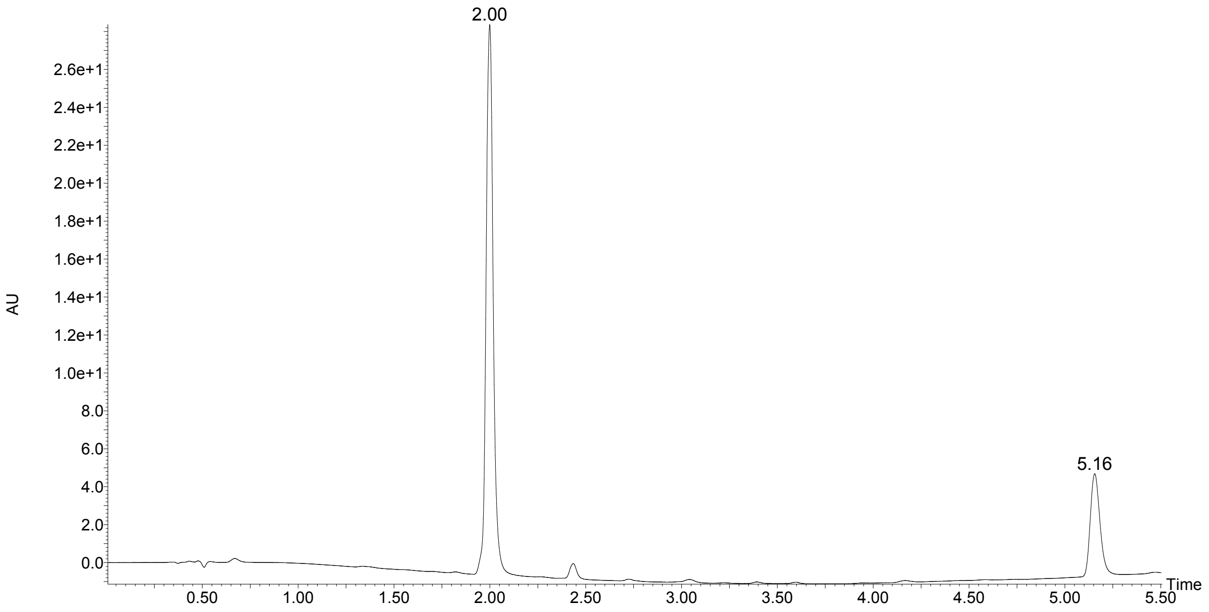

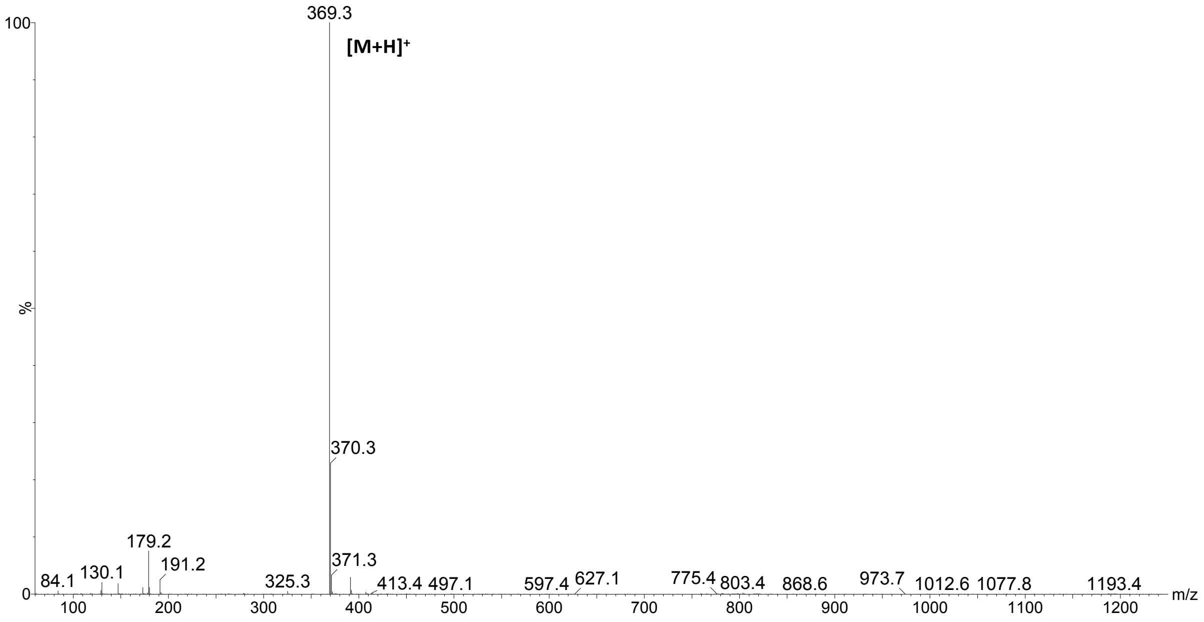

Resins 2–6

A resin was subjected to 50% piperidine in DMF for 30 minutes and subsequently washed with DMF (10x 10 mL) and DCM (10x 10 mL). Afterwards, it was reacted with a suitable *N*-Fmoc-protected amino acid or PEG spacer (2.0 mmol), HOBt (2.0 mmol), and DIC (2.0 mmol) in DMF (5 mL) and DCM (5 mL). The reaction mixture was shaken for 2 hours at lab temperature. Afterwards, a solid support was washed with DMF (10x 10 mL) and DCM (10x 10 mL).

Figure S2. LC-MS analysis of chemically cleaved peptide from Resin 6 (Rt = 2.70 min), Mtt protecting group (Rt = 5.13 min).

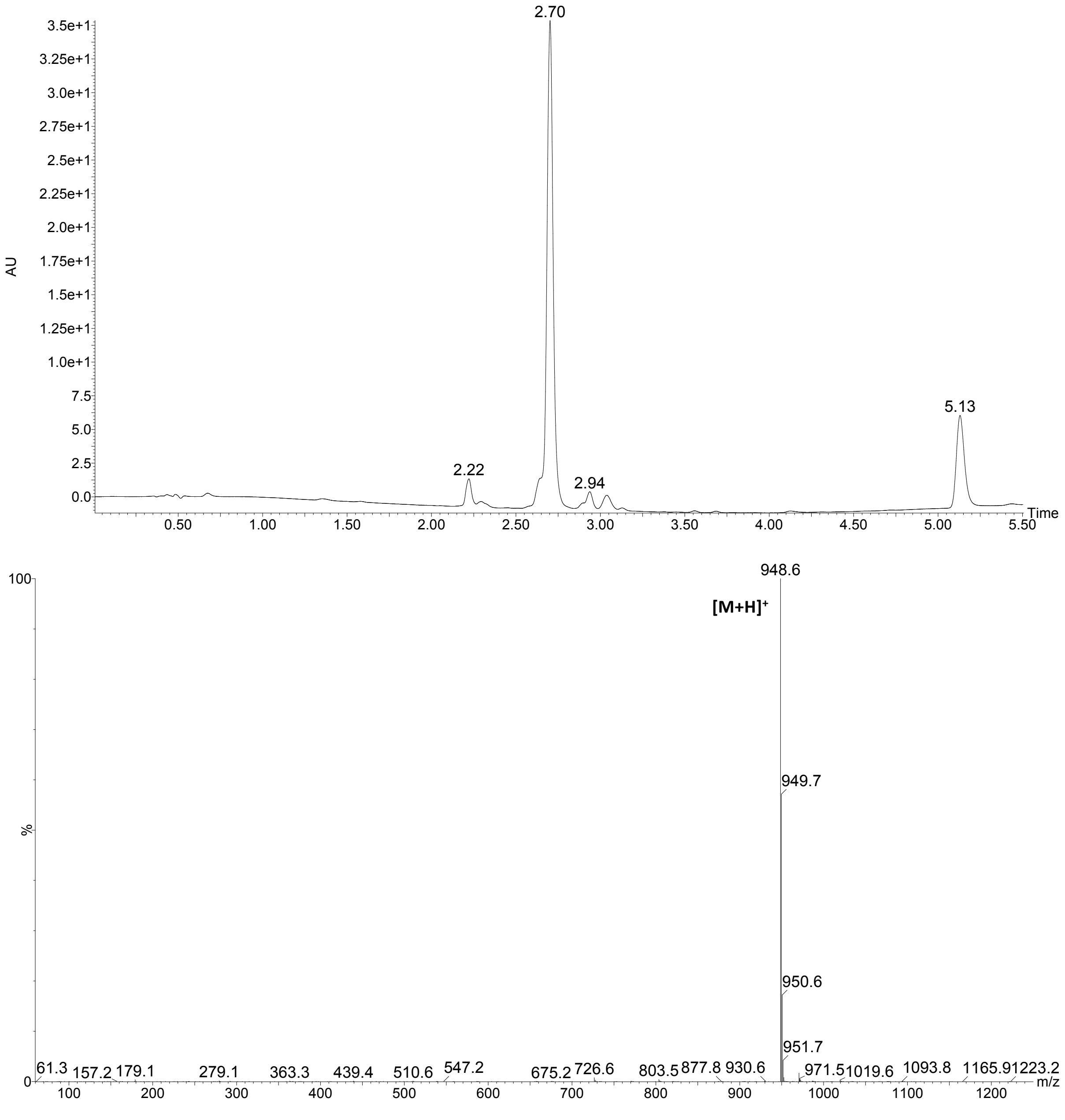

Resin 7

Resin 6 was subjected to 50% piperidine in DMF for 30 minutes and subsequently washed with DMF (10x 10 mL) and DCM (10x 10 mL). Afterwards, the resin was reacted with DEAC (2.0 mmol), HOBt (2.0 mmol), DMAP (2.0 mmol), and DIC (2.0 mmol) in DMF (2 mL), DCM (4 mL), and DMSO (4 mL). The reaction mixture was shaken for 16 hours at lab temperature. Then, solid support was washed with DMSO (5x 10 mL), DMF (10x 10 mL) and DCM (10x 10 mL).

Figure S3. LC-MS analysis of chemically cleaved peptide from Resin 7 (Rt = 2.42 min), Mtt protecting group (Rt = 5.07 min).

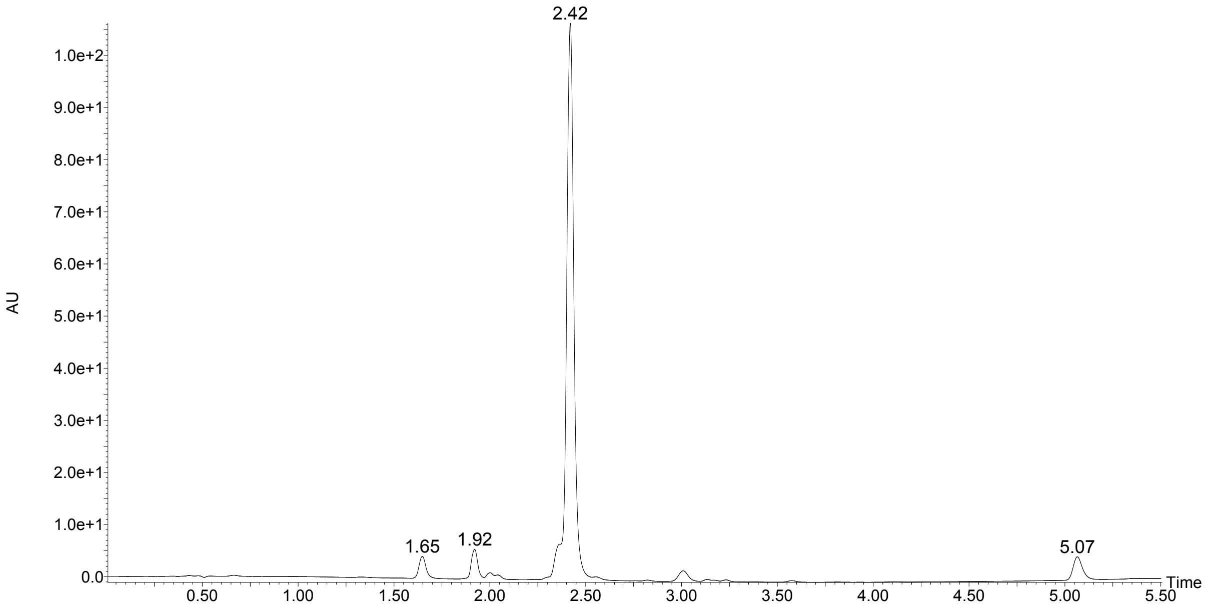

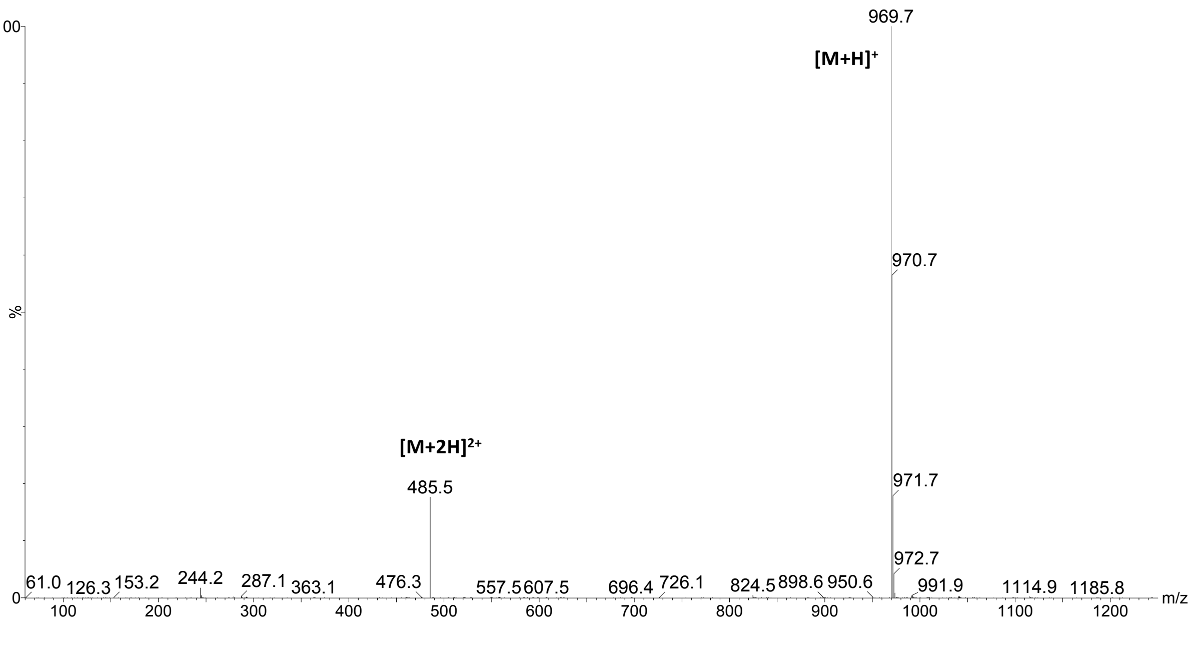

<u>Resin 8</u>

To Resin 7, 1,2-dichloroethane (13 mL), triethylsilane (4 mL), hexafluoroisopropanol (2 mL) and trifluoroethanol (1 mL) were added. The heterogeneous mixture was at 60 °C shaken for the time period of 6 hours. Then, a solid support was washed with DCM (10x 10 mL).

Figure S4. LC-MS analysis of chemically cleaved peptide from Resin 8 (Rt = 2.52 min).

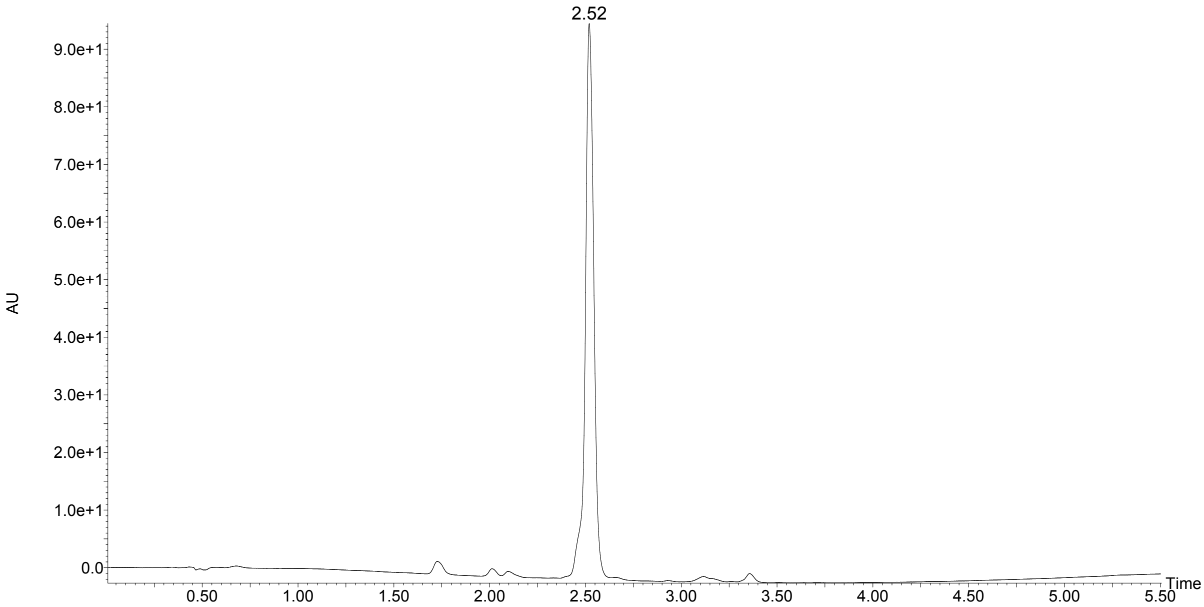

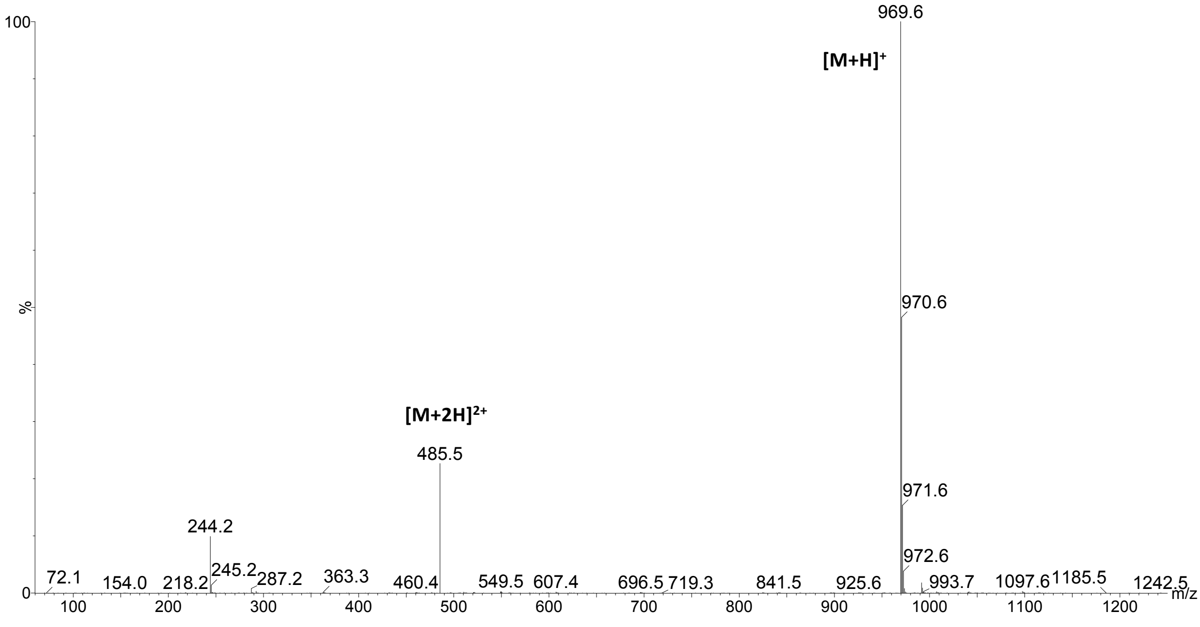

Resins 9–11

N-Fmoc-protected PEG spacer or amino acid (2.0 mmol), HOBt (2.0 mmol), and DIC (2.0 mmol) were reacted with a resin for the time period of 3 hours. Afterwards, a resin was washed with DMF (10x 10 mL) and DCM (10x 10 mL). Resins 9 and 10 were subjected to 50% piperidine in DMF for 30 minutes, and subsequently washed with DMF (10x 10 mL) and DCM (10x 10 mL), before their acylation with a suitable amino acid.

Figure S5. LC-MS analysis of chemically cleaved peptide from Resin 11 (Rt = 3.23 min), Mtt protecting group (Rt = 5.25 min).

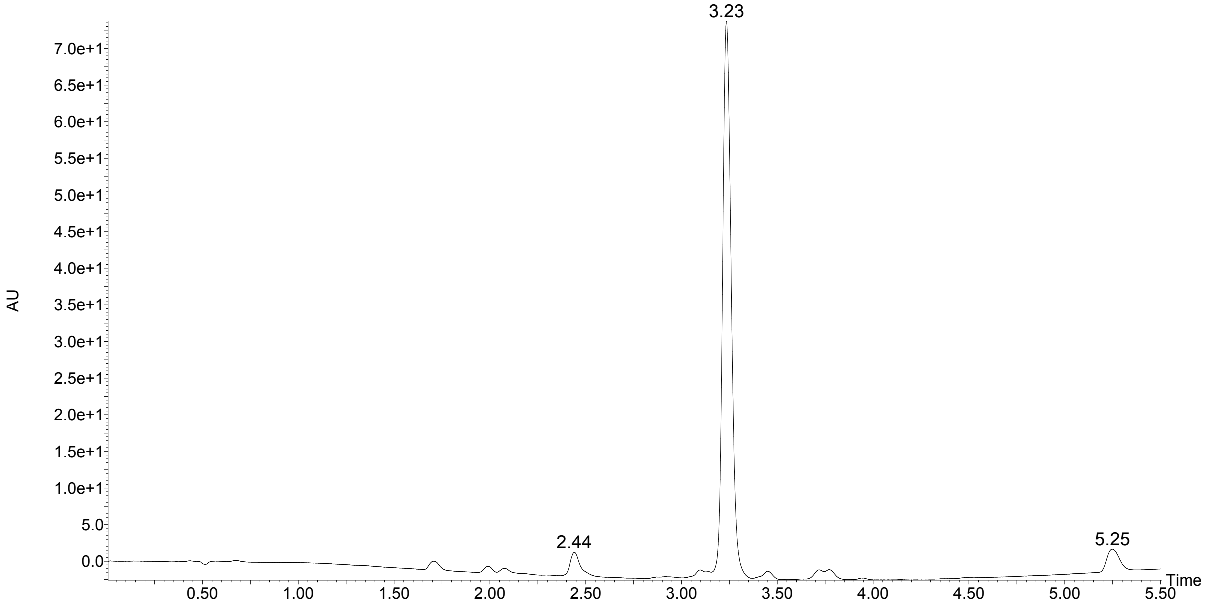

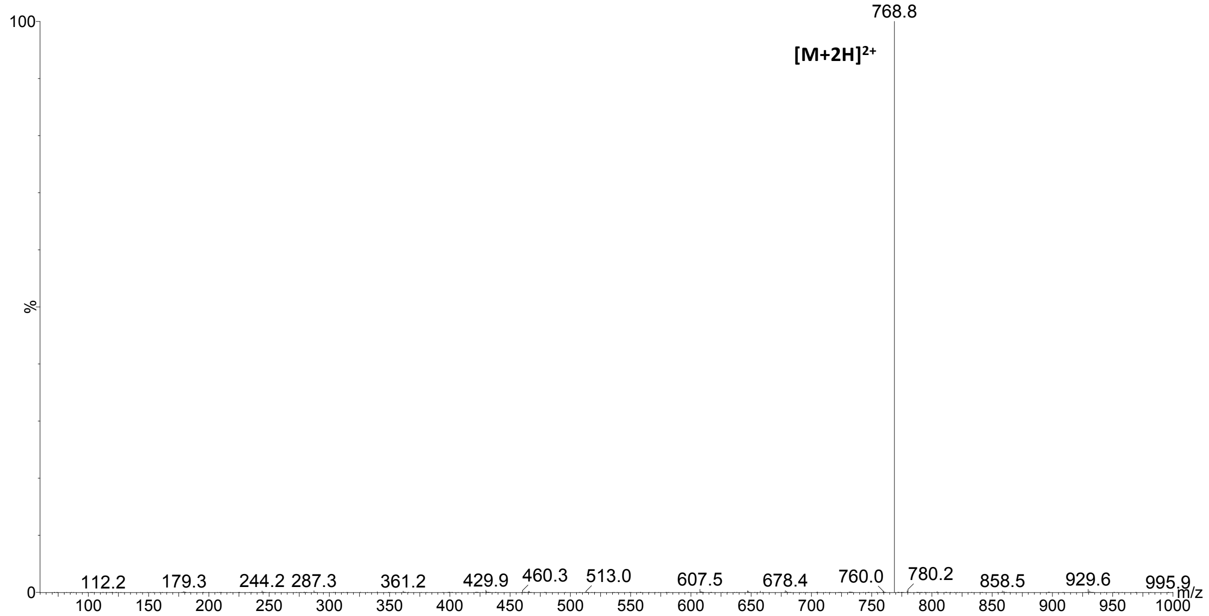

Resin 12

Resin 11 was subjected to 50% piperidine in DMF for 30 minutes and subsequently washed with DMF (10x 10 mL) and DCM (10x 10 mL). Afterwards, the resin was reacted with FL (2.0 mmol), HOBt (2.0 mmol), DMAP (2.0 mmol), and DIC (2.0 mmol) in DMF (2 mL), DCM (4 mL), and DMSO (4 mL). The reaction mixture was shaken for 16 hours at lab temperature. Then, solid support was washed with DMSO (5x 10 mL), DMF (10x 10 mL) and DCM (10x 10 mL).

Figure S6. LC-MS analysis of chemically cleaved peptide from Resin 12 (Rt = 2.44 min), Mtt protecting group (Rt = 5.24 min).

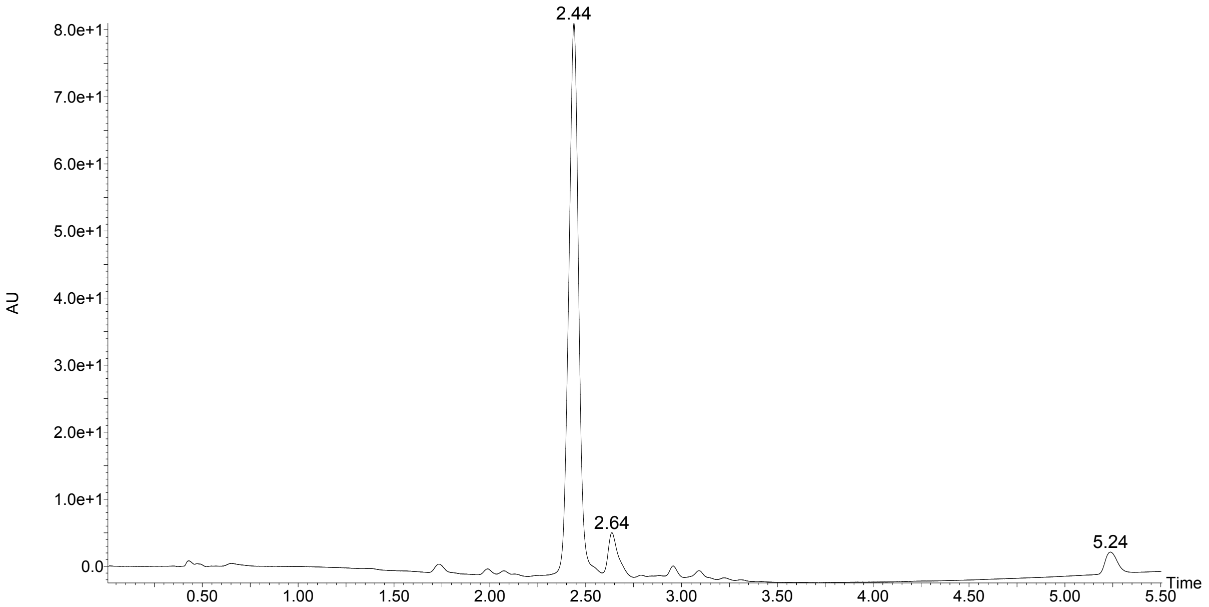

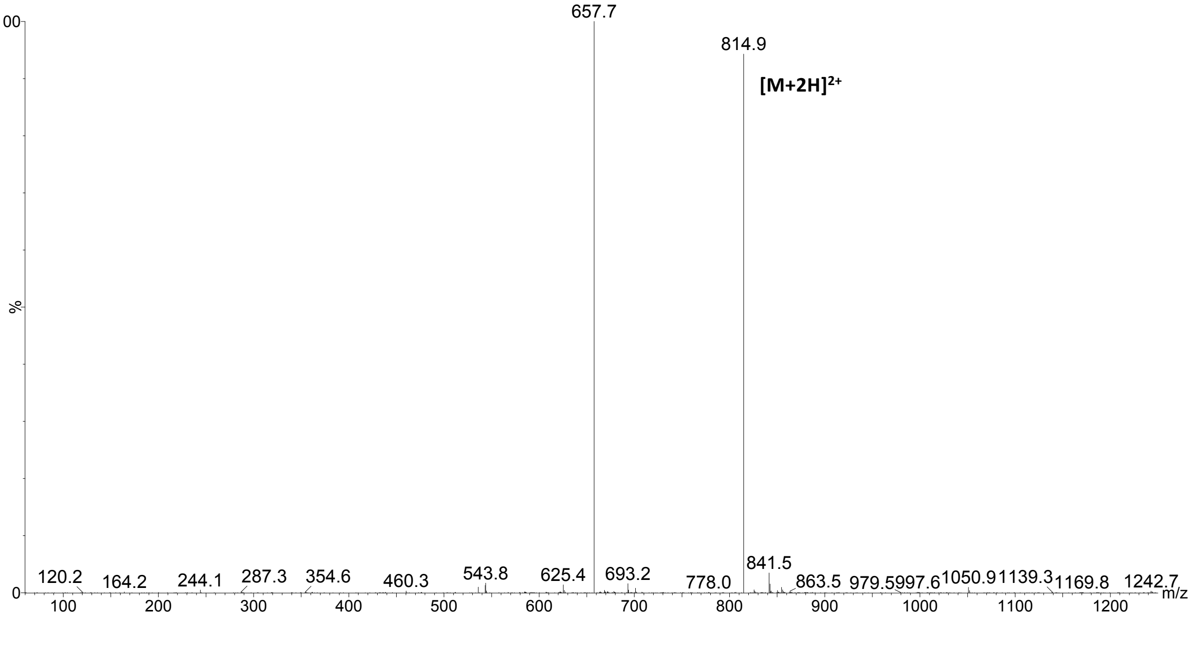

Resin 13

To Resin 12, 1,2-dichloroethane (13 mL), triethylsilane (4 mL), hexafluoroisopropanol (2 mL) and trifluoroethanol (1 mL) were added. The heterogeneous mixture was at 60 °C shaken for the time period of 6 hours. Then, a solid support was washed with DCM (10x 10 mL).

Figure S7. LC-MS analysis of chemically cleaved peptide from Resin 13 (Rt = 2.42 min).

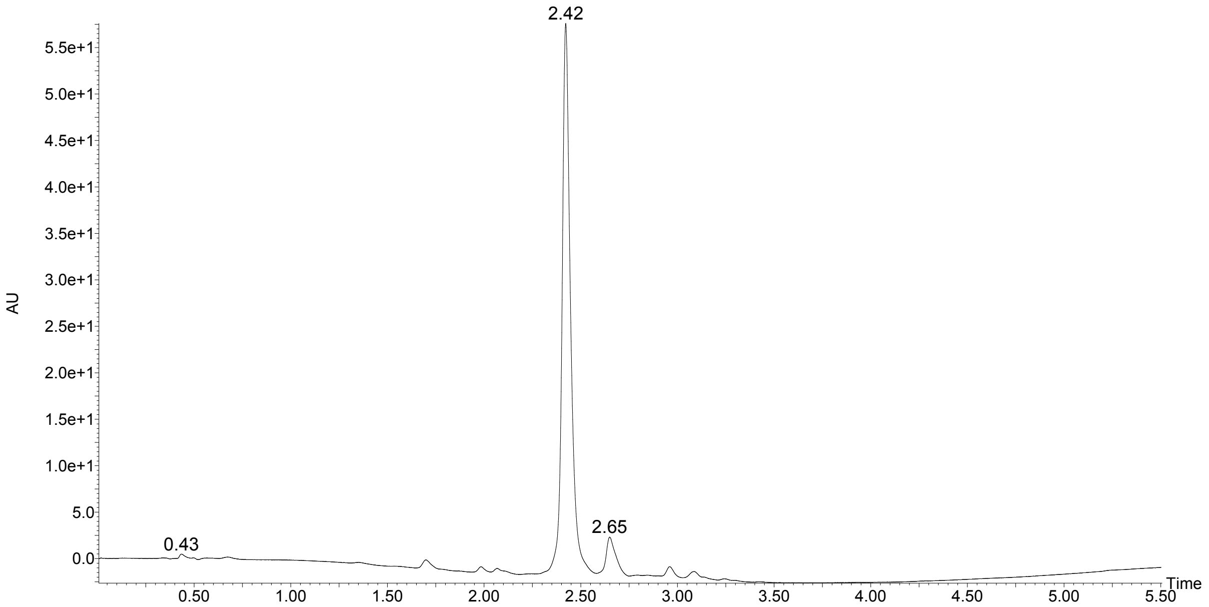

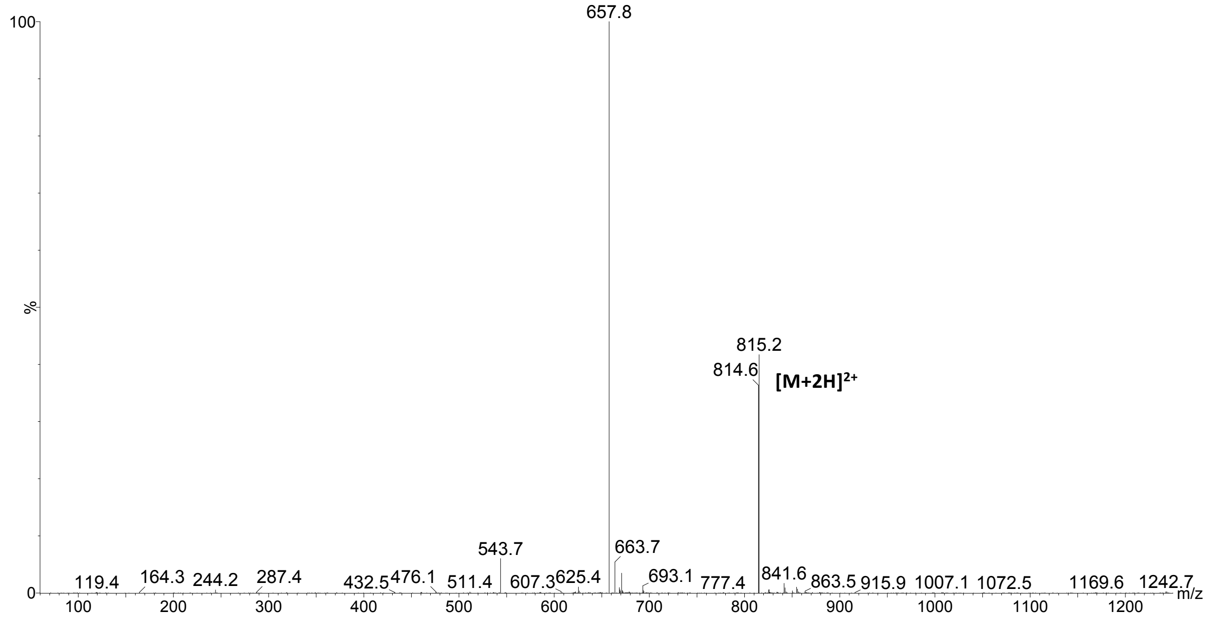

Resins 14 and 15

N-Fmoc-protected PEG spacer or sarcosine (2.0 mmol), HOBt (2.0 mmol), and DIC (2.0 mmol) were reacted with a resin for 16 hours and 3 hours, respectively. Then, a resin was washed with DMF (10x 10 mL) and DCM (10x 10 mL). Resin 14 was subjected to 50% piperidine in DMF for 30 minutes, and subsequently washed with DMF (10x 10 mL) and DCM (10x 10 mL), before its acylation with sarcosine.

Figure S8. LC-MS analysis of chemically cleaved peptide from Resin 15 (Rt = 3.02 min).

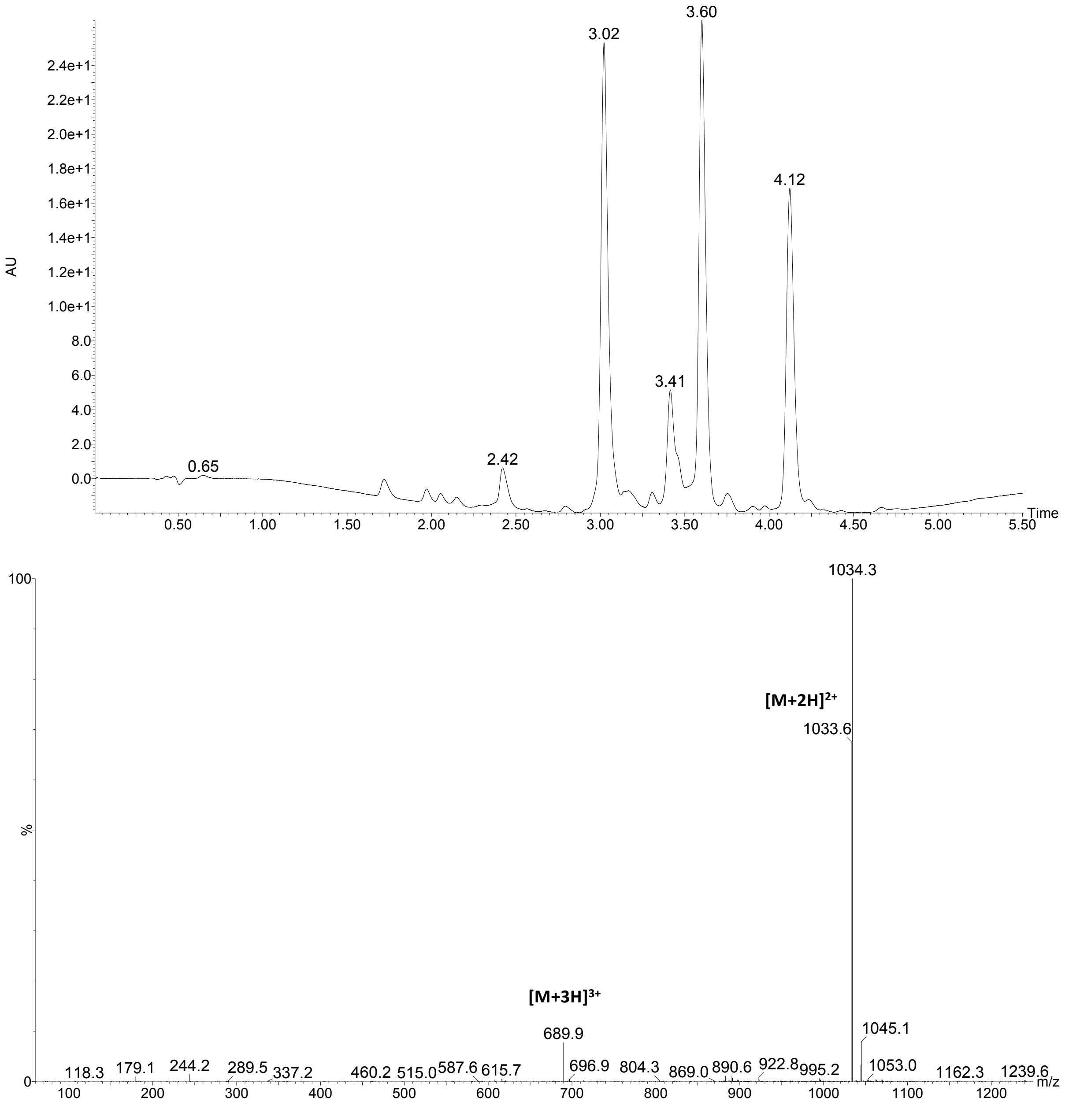

Resin 16

Resin 15 was subjected to 50% piperidine in DMF for 30 minutes and subsequently washed with DMF (10x 10 mL) and DCM (10x 10 mL). Afterwards, the resin was reacted with BC (2.0 mmol), HOBt (2.0 mmol), DMAP (2.0 mmol), and DIC (2.0 mmol) in DMF (2 mL), DCM (4 mL), and DMSO (4 mL). The reaction mixture was shaken for 16 hours at lab temperature. Then, solid support was washed with DMSO (5x 10 mL), DMF (10x 10 mL) and DCM (10x 10 mL).

Figure S9. LC-MS analysis of chemically cleaved peptide from Resin 16 (Rt = 3.42 min).

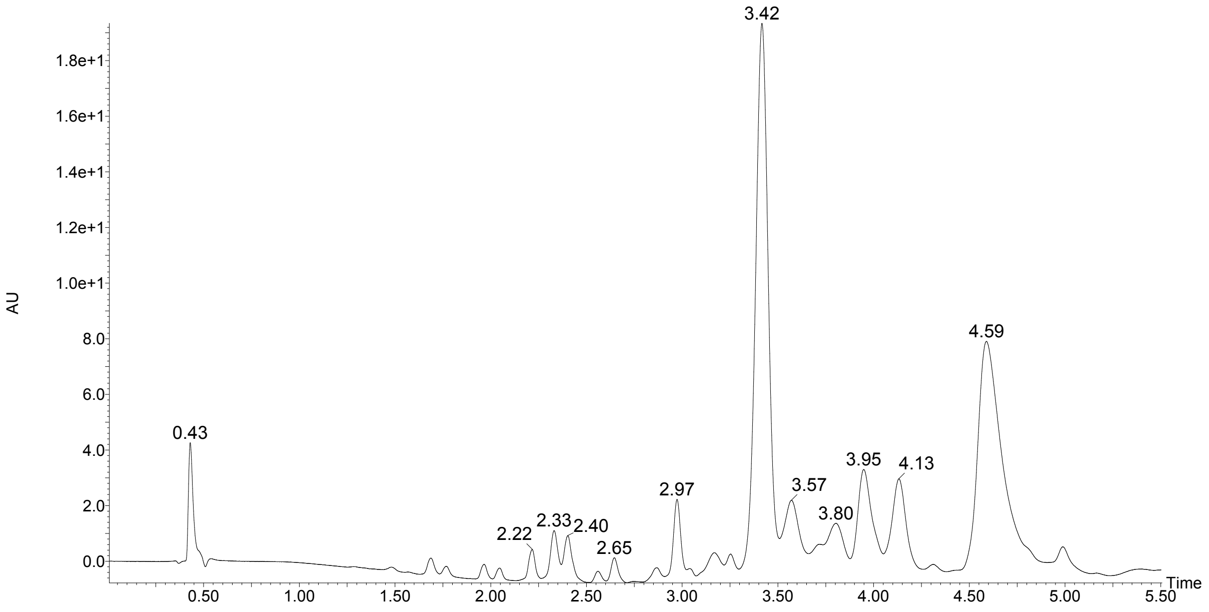

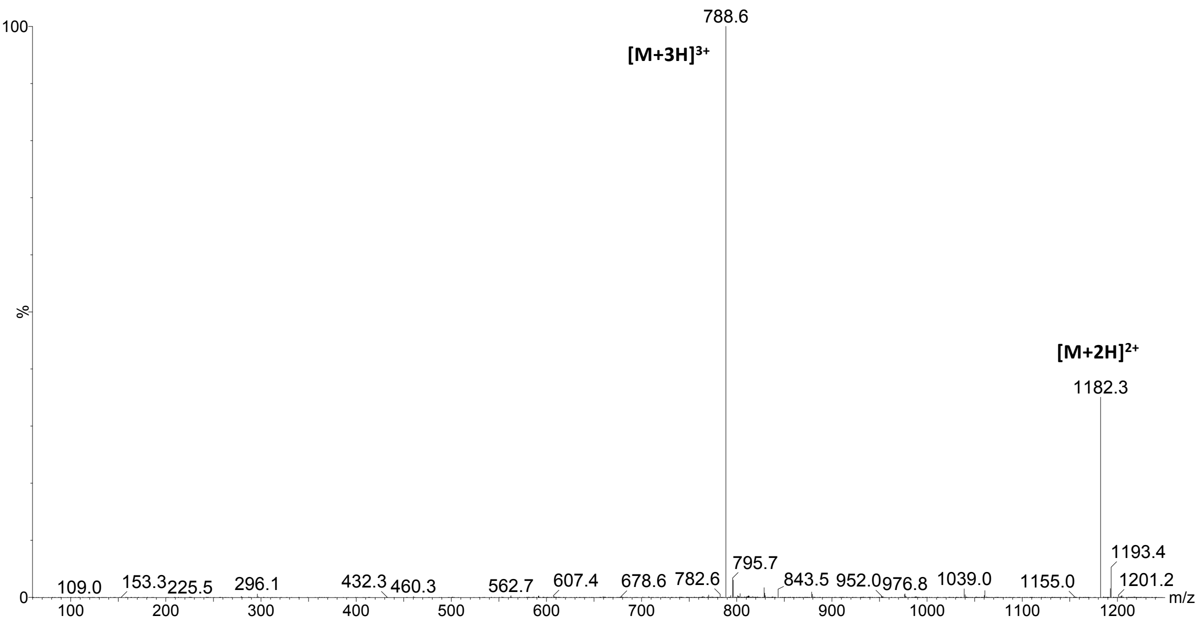

3. LC-MS analyses – Intact CP and C probes

Method: *Ammonium acetate (10 mM) in ultrapure water and acetonitrile (gradient 20–80% during the first 4.5 min)*
LC-MS column: *50 x 3.0 mm XSelect HSS T3 2.5 µm XP, Waters, Borehamwood, UK*

Figure S10. LC-MS analysis of isolated intact CP probe after freeze-drying (Rt = 3.29 min).

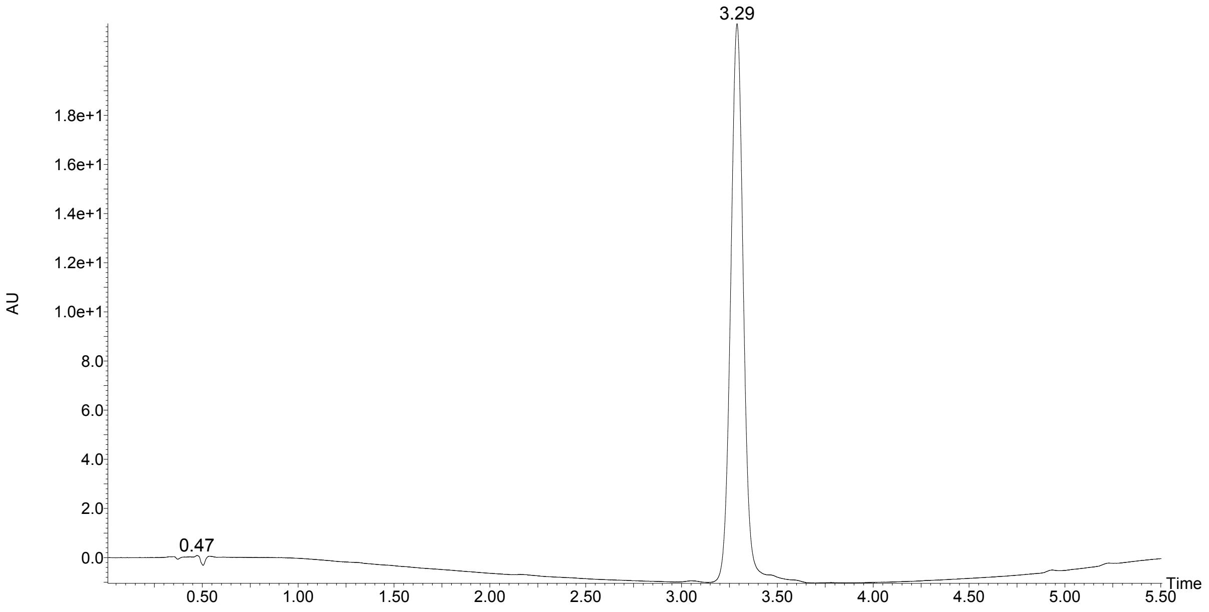

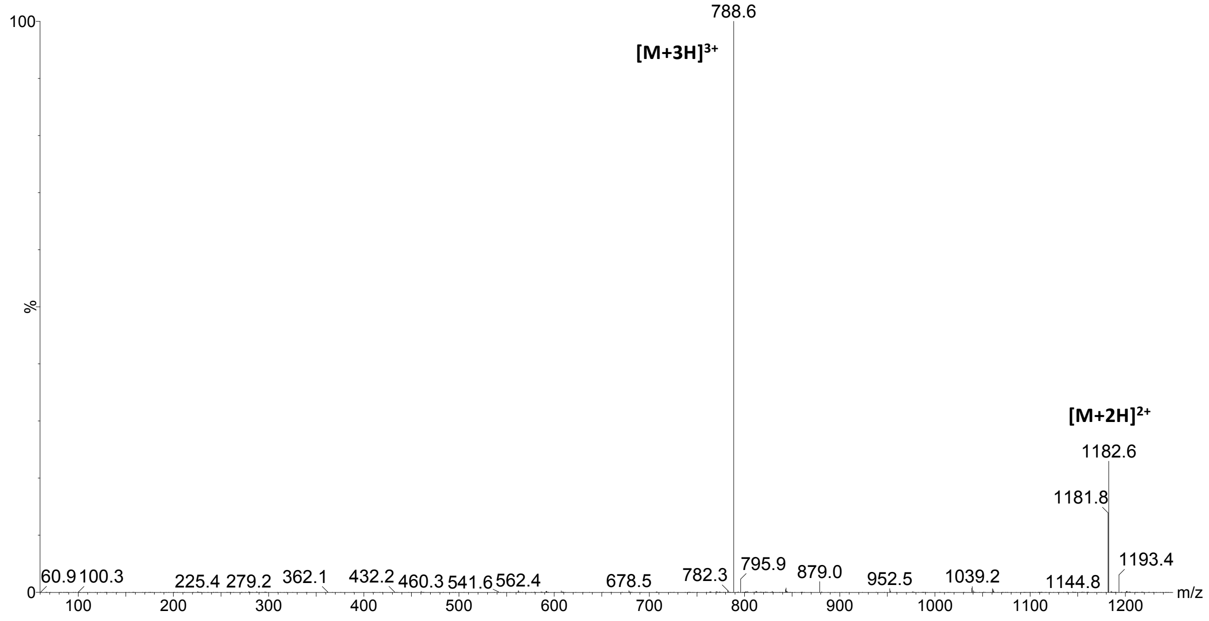

Figure S11. LC-MS analysis of isolated intact C probe after freeze-drying (Rt = 2.33 min).

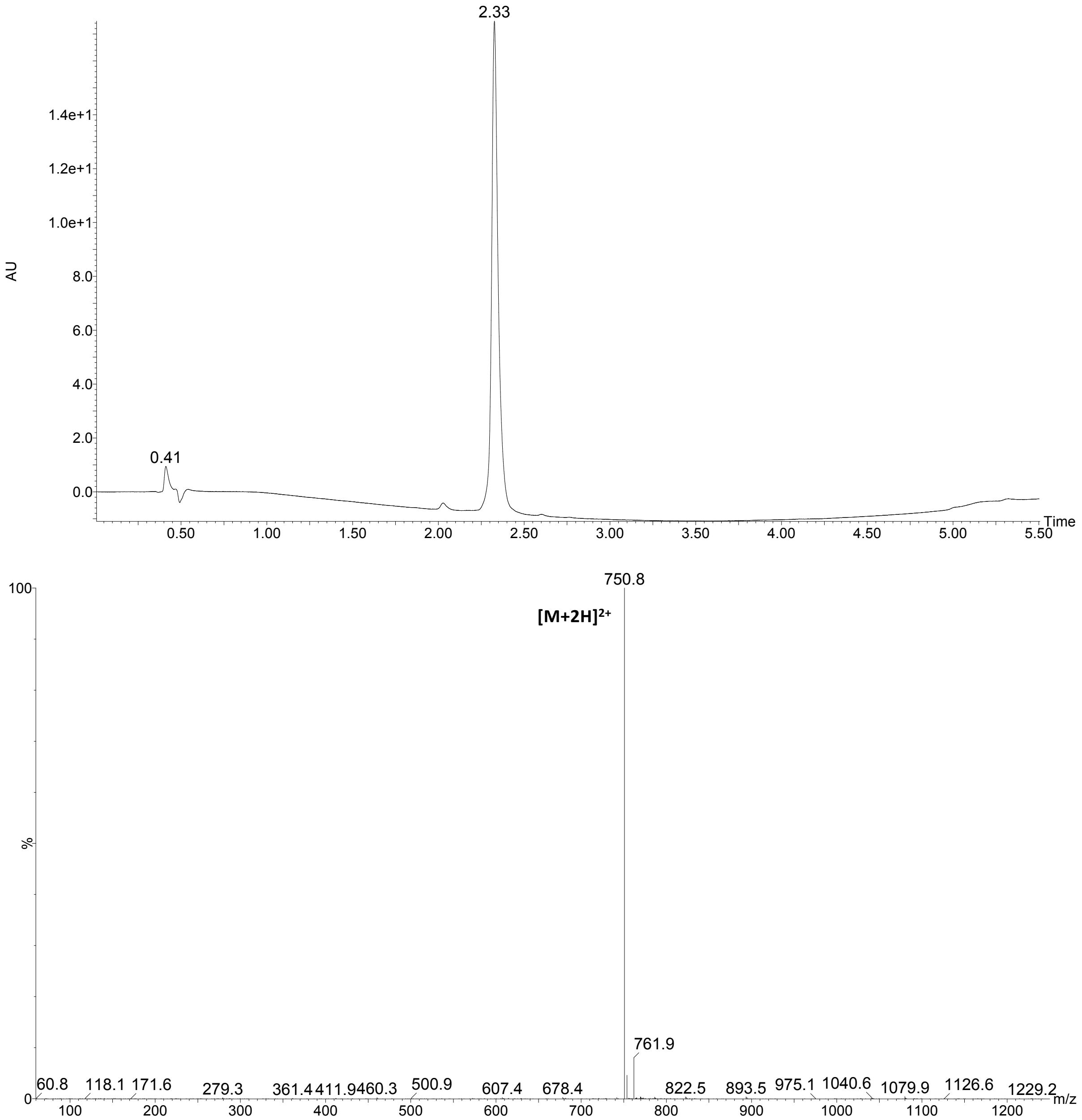

4. LC-MS analyses – Application

Method: Ammonium acetate (10 mM) in ultrapure water and acetonitrile (gradient 20–80% during the first 4.5 min)
LC-MS column: 50 x 3.0 mm XSelect HSS T3 2.5 µm XP, Waters, Borehamwood, UK

Figure S12. LC-MS analysis of CP probe treated with chymotrypsin (500 µg/mL; 60 min; 37 °C).

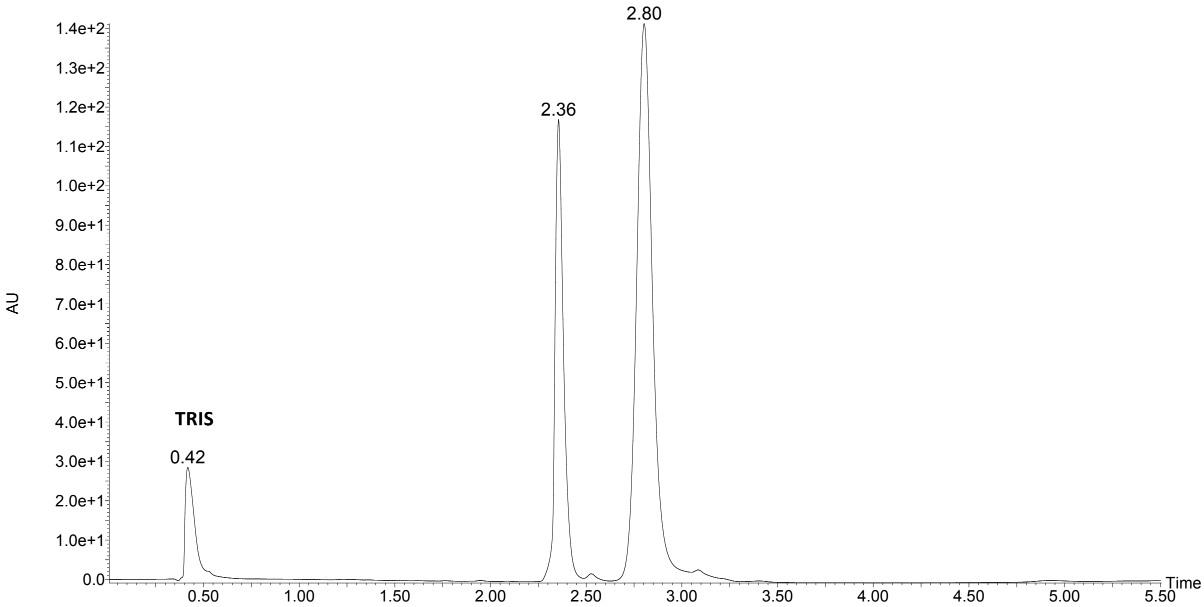

Fragment 1 (Rt = 2.36 min)

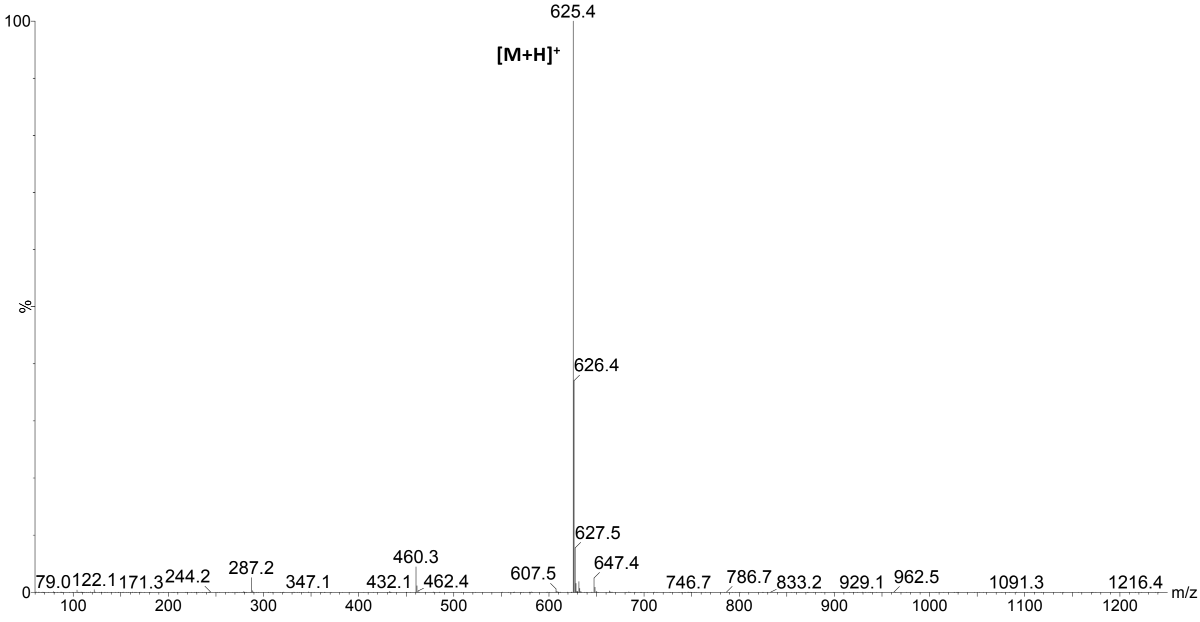

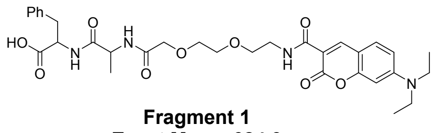

Fragment 1
Exact Mass: 624.3

Fragment 2 (Rt = 2.80 min)

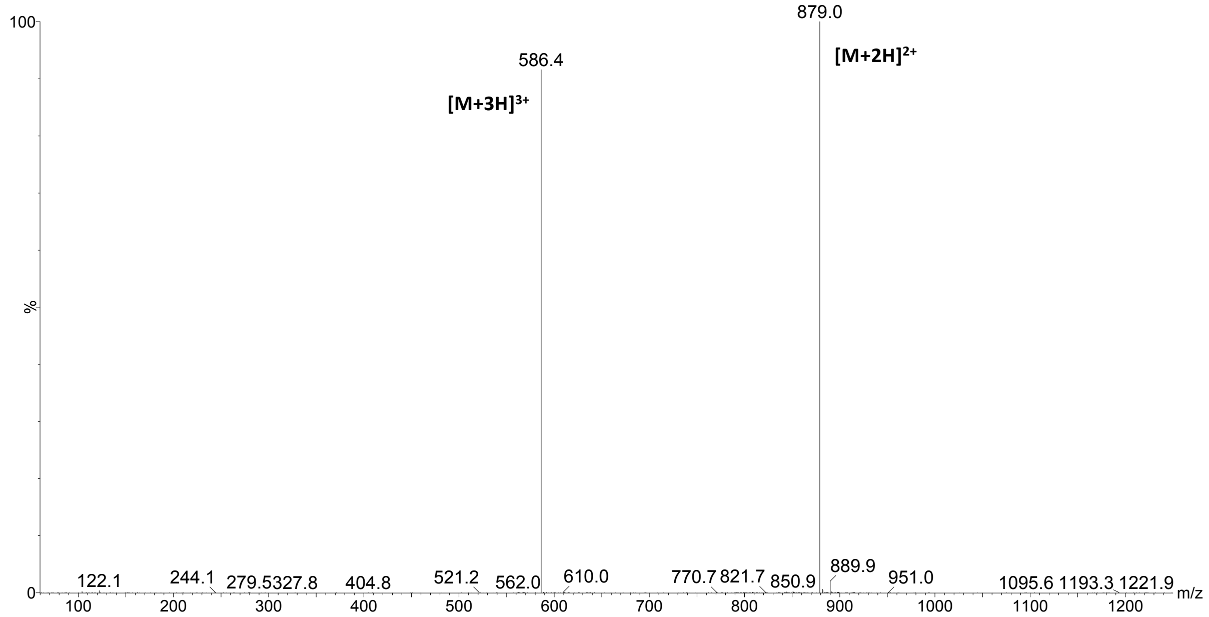

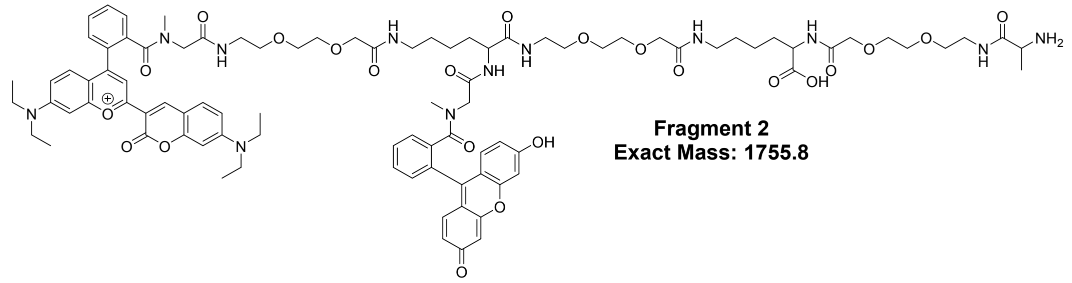

Fragment 2
Exact Mass: 1755.8

Figure S13. LC-MS analysis of CP probe treated with hydrogen peroxide (20 mM; 90 min; 37 °C).

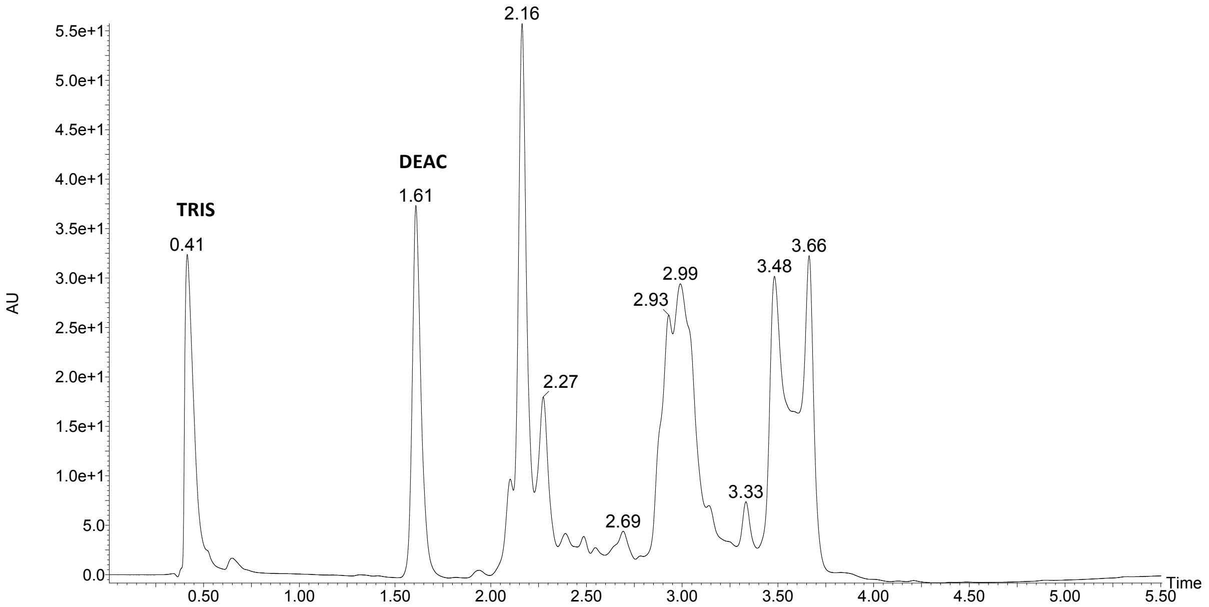

Compound I (Rt = 2.16 min)

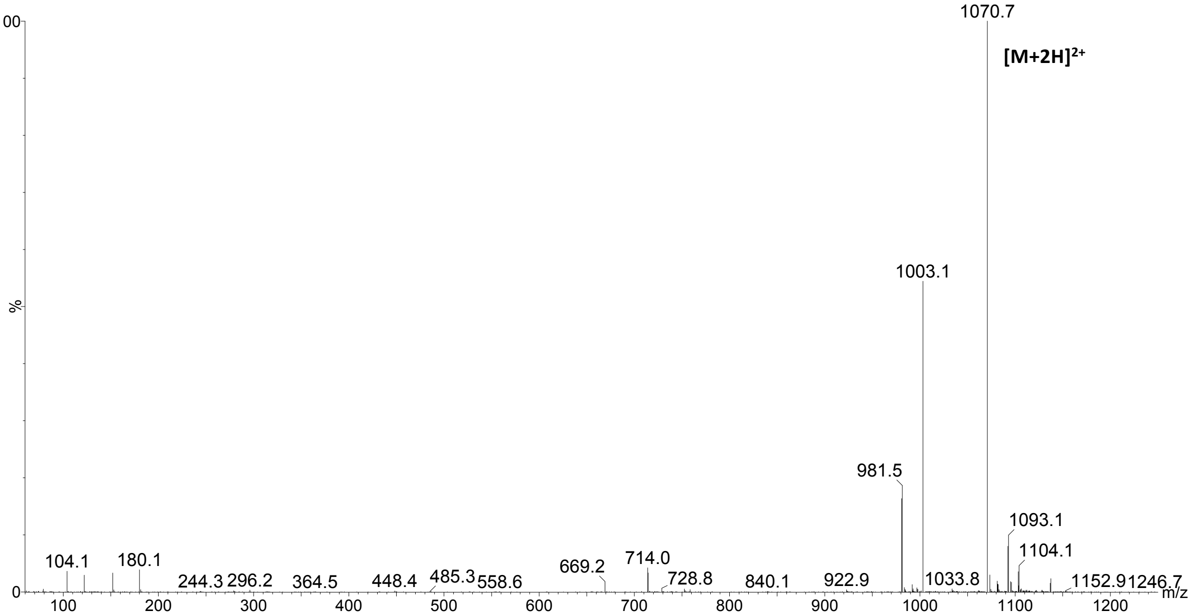

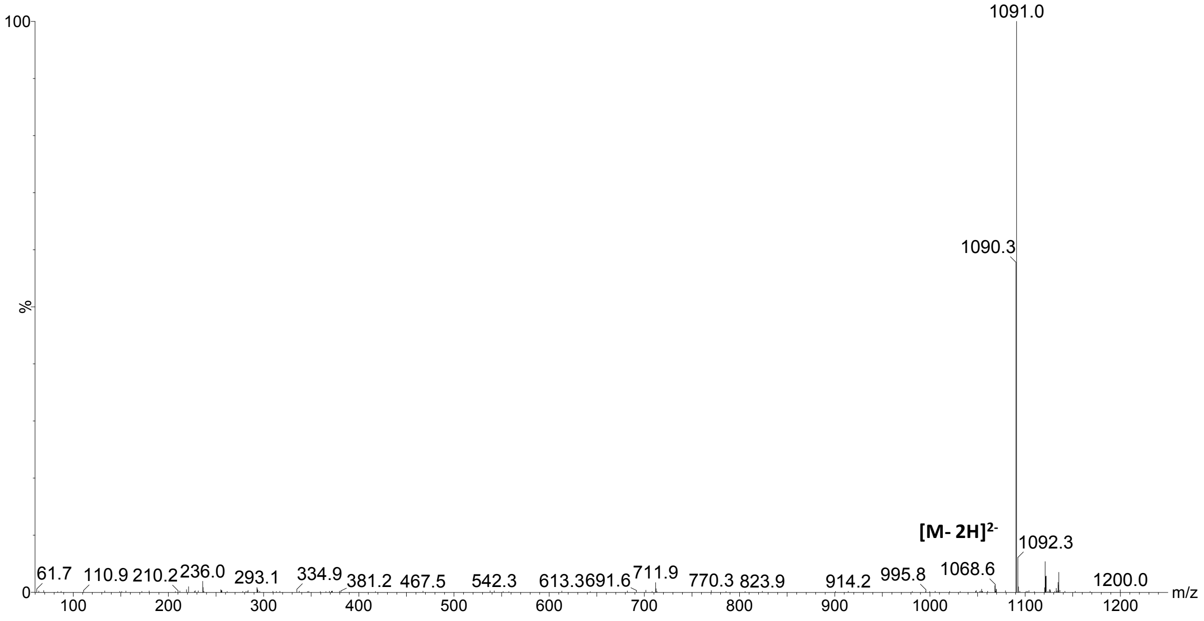

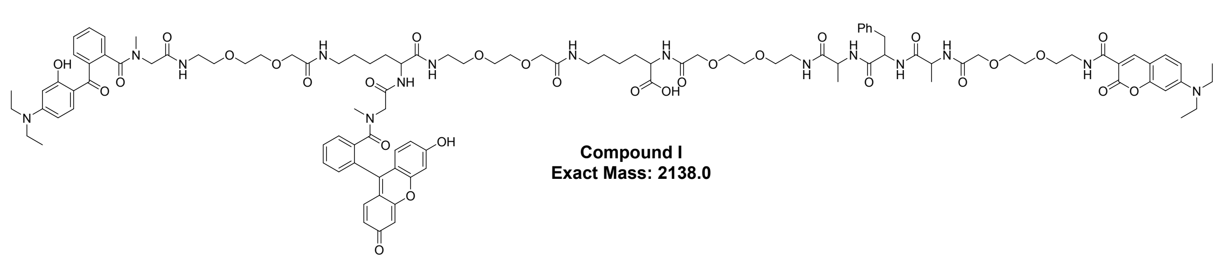

Compound I
Exact Mass: 2138.0

Compound II (Rt = 2.27 min)

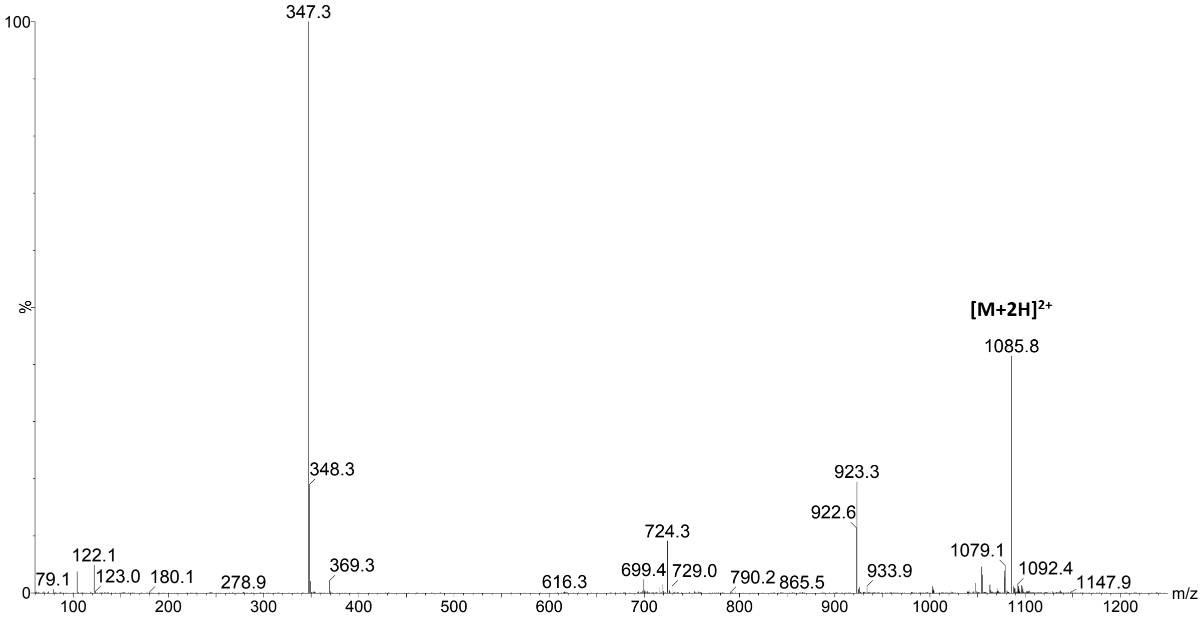

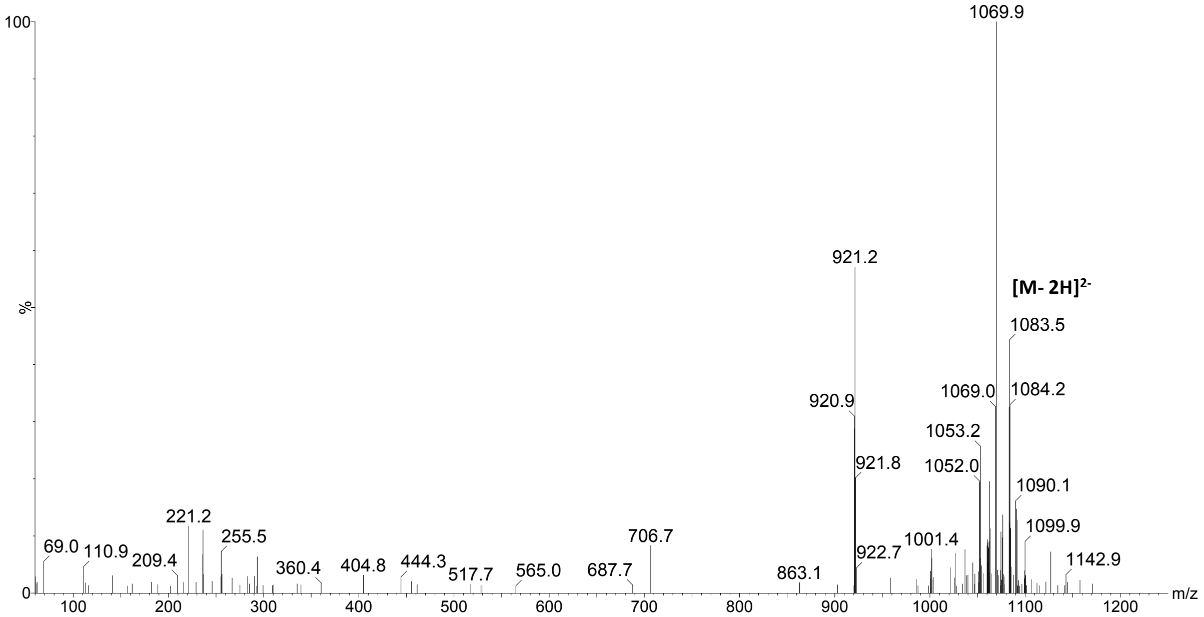

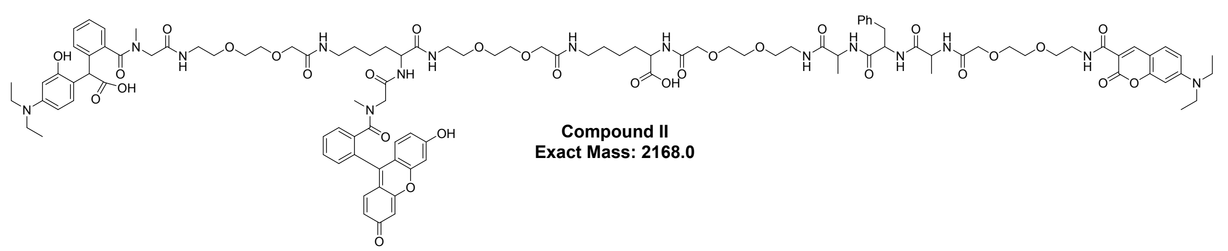

Compound II
Exact Mass: 2168.0

Compound III (Rt = 2.90–3.10 min)

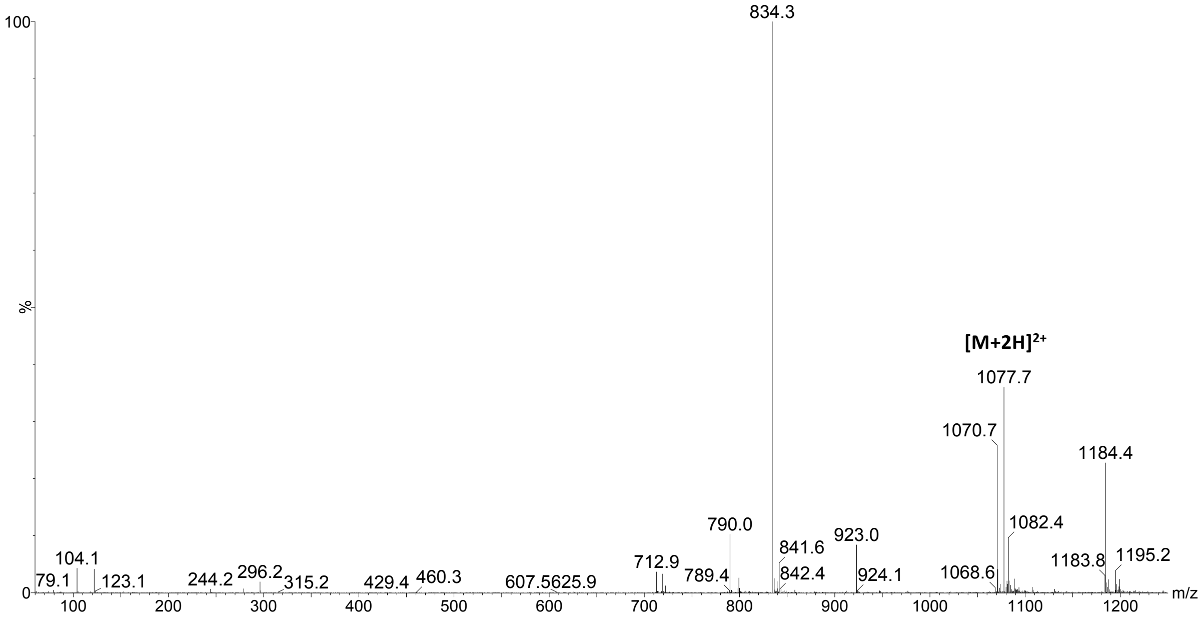

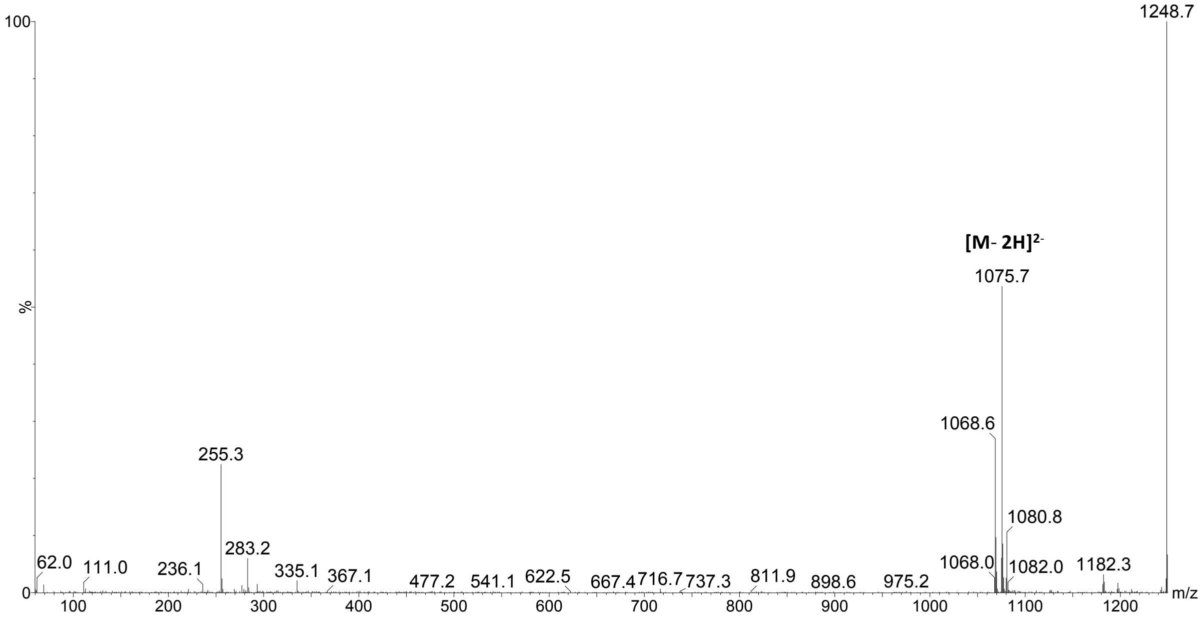

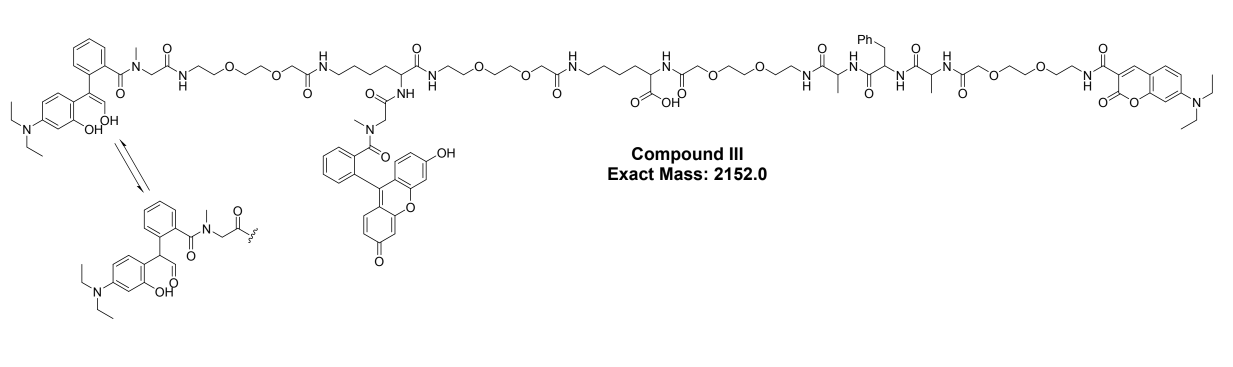

Compound IV (Rt = 3.40–3.70 min)

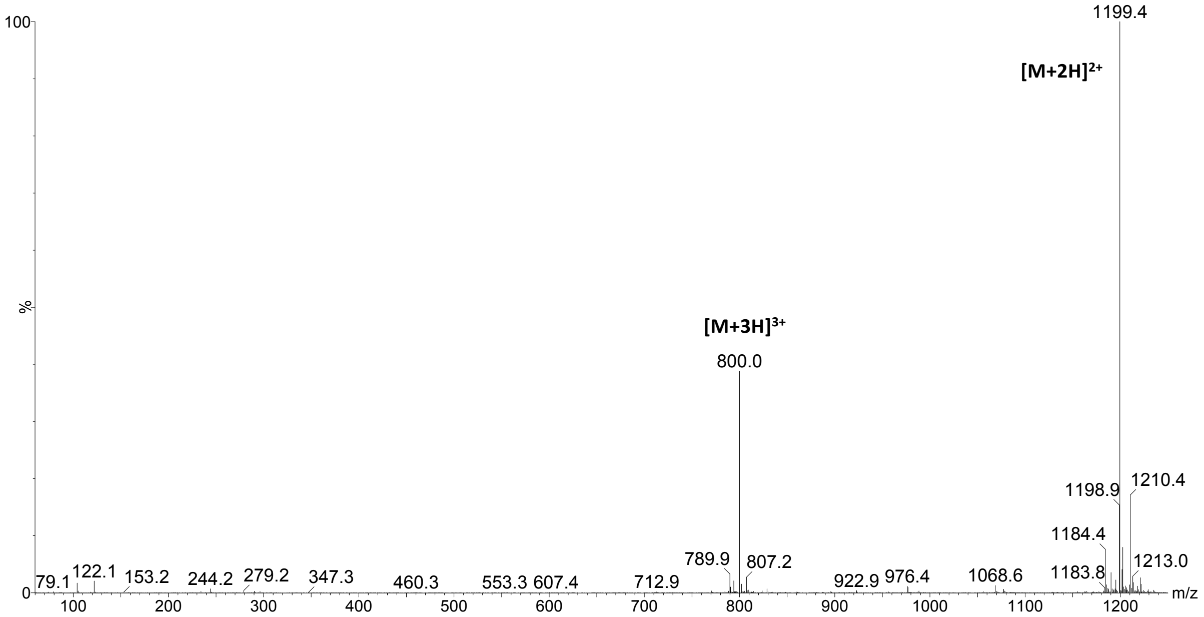

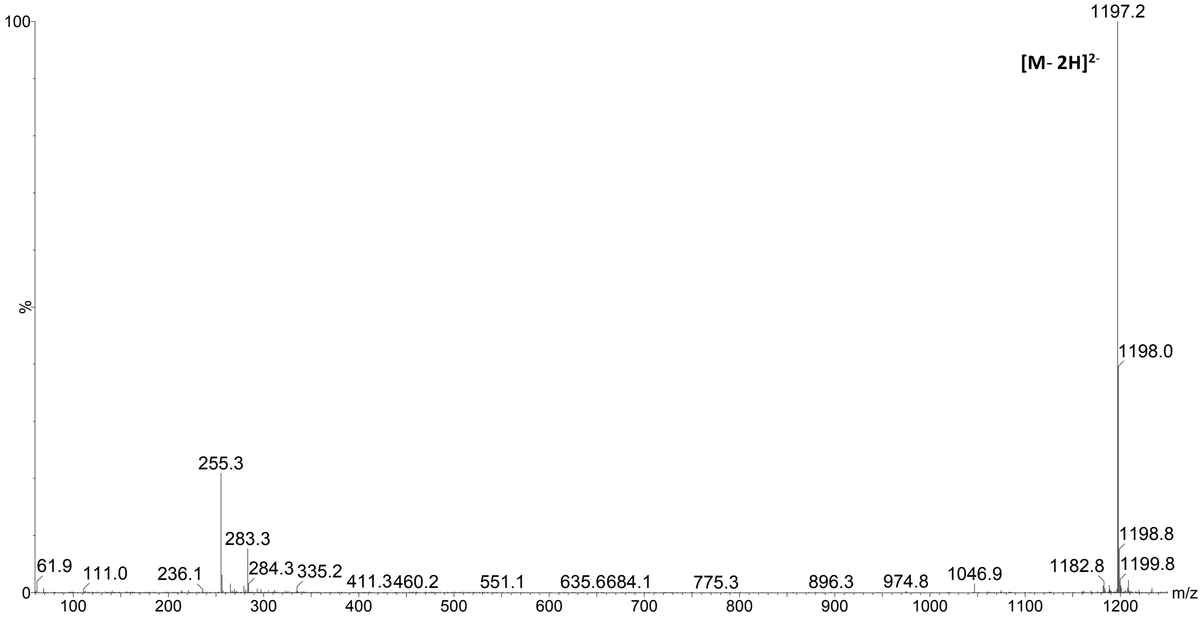

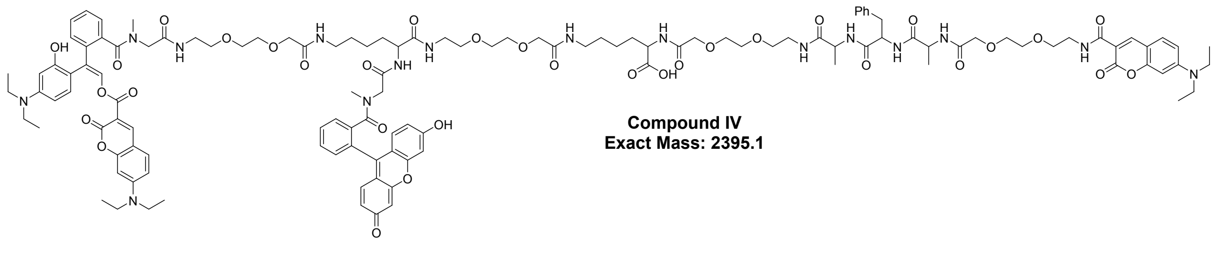

Compound IV
Exact Mass: 2395.1

Figure S14. LC-MS analysis of C probe treated with chymotrypsin (500 µg/mL; 60 min; 37 °C).

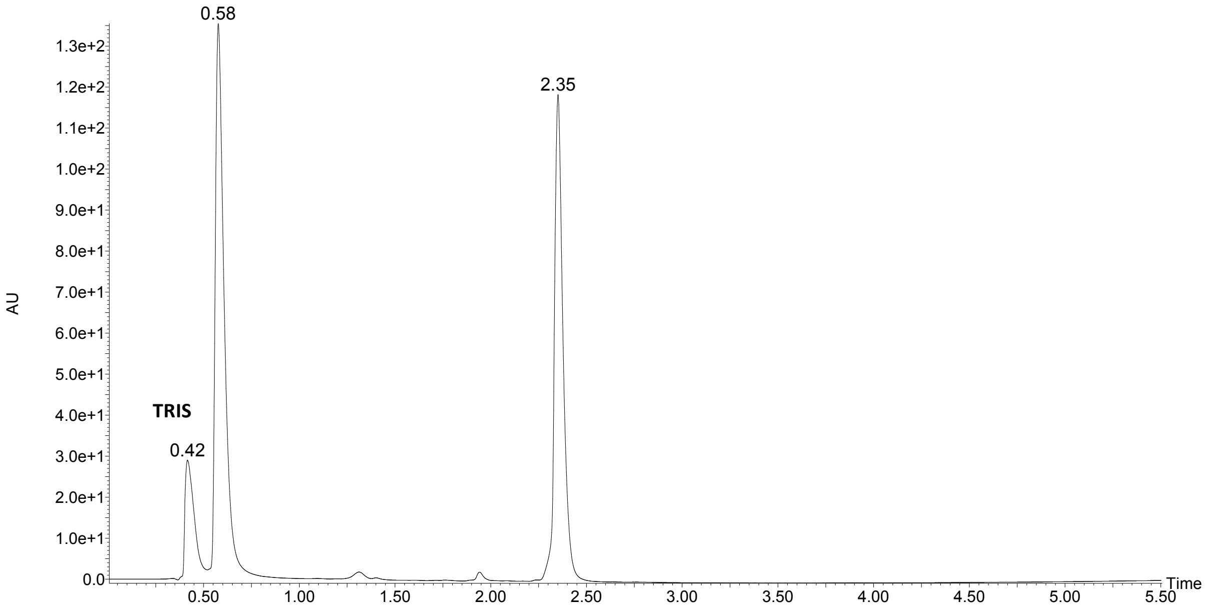

Fragment 1 (Rt = 0.58 min)

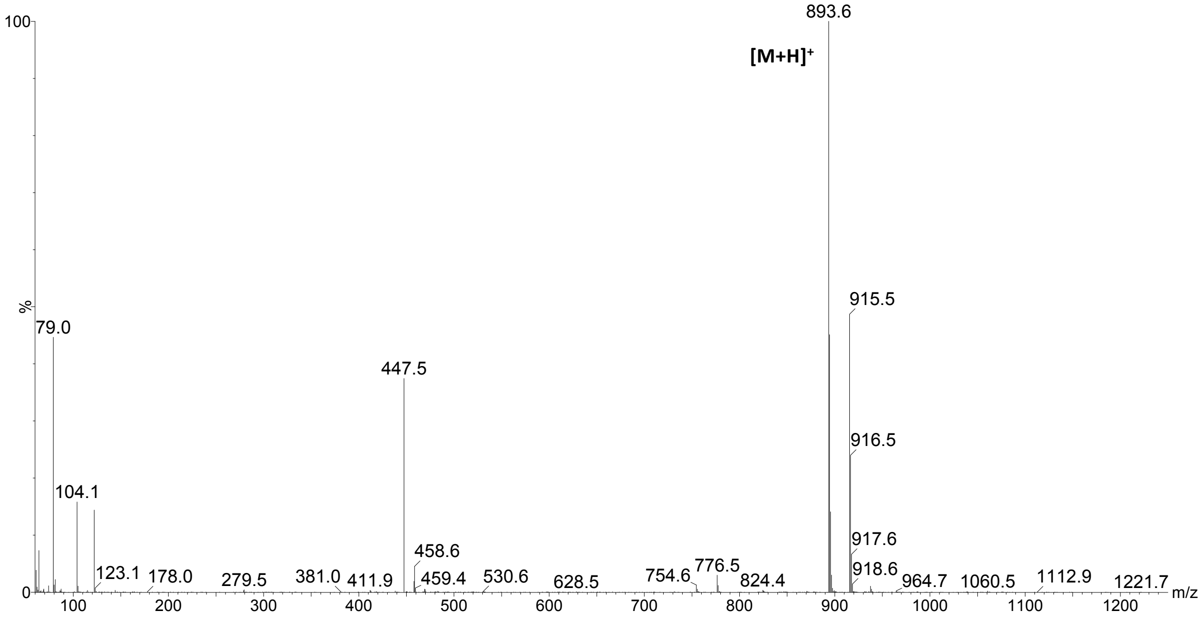

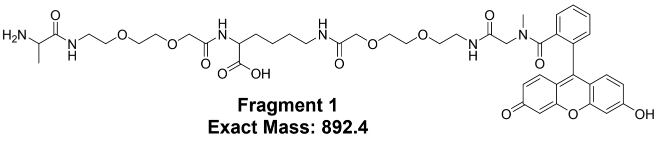

Fragment 1
Exact Mass: 892.4

Fragment 2 (Rt = 2.35 min)

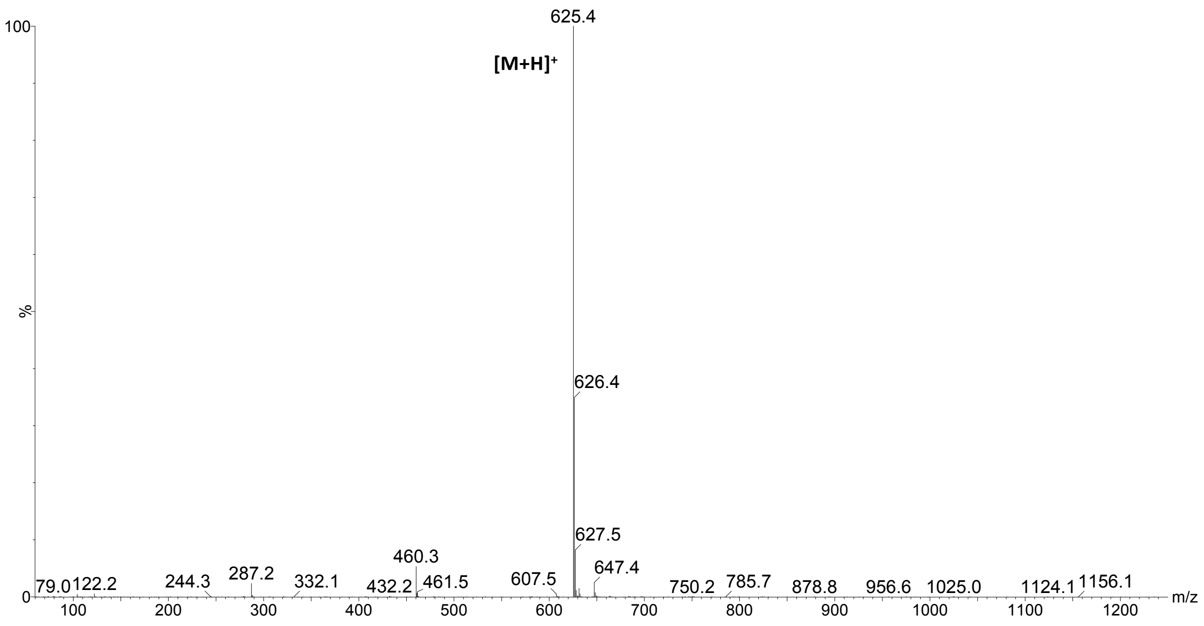

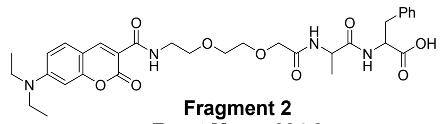

Fragment 2
Exact Mass: 624.3

Figure S15. LC-MS analysis of C probe treated with hydrogen peroxide (20 mM; 90 min; 37 °C).

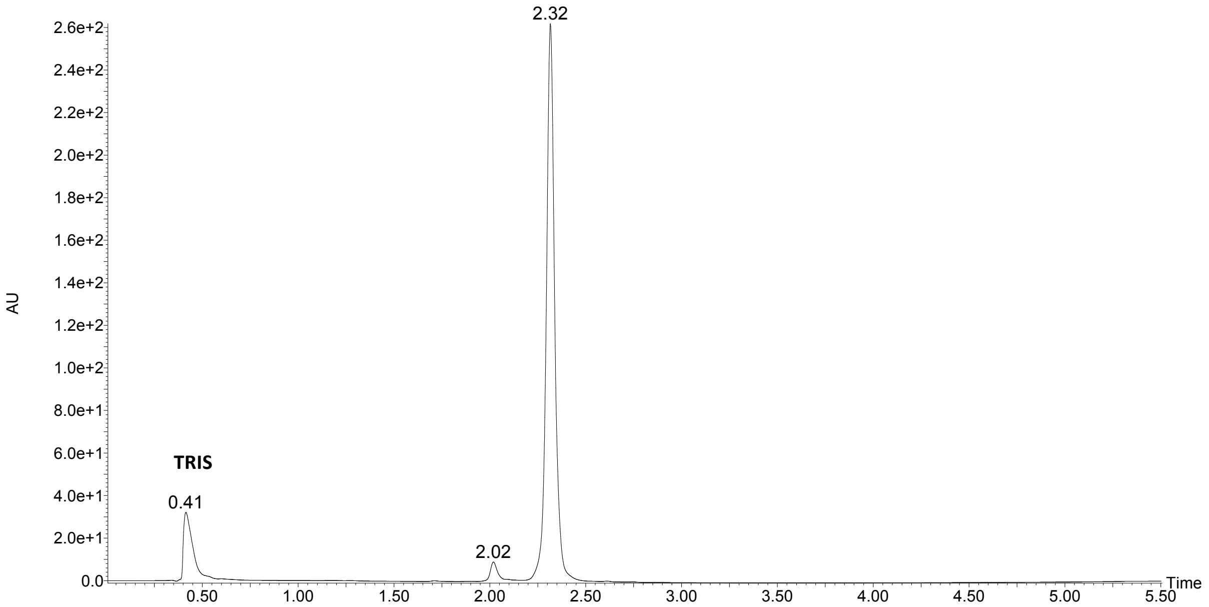

Intact probe C (Rt = 2.32 min)

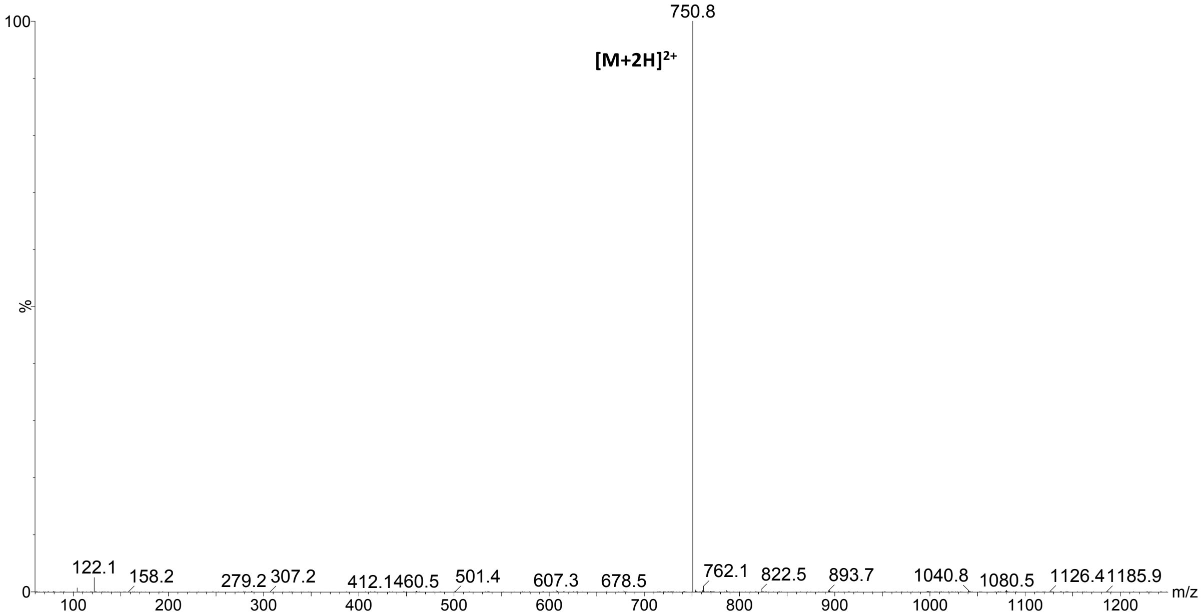

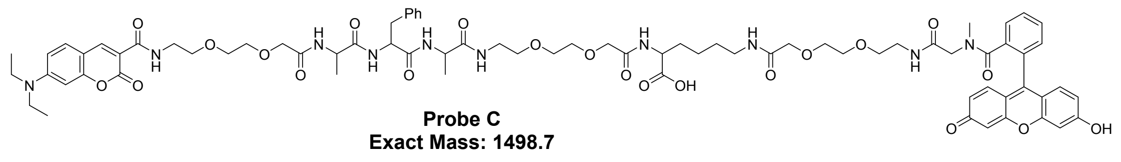

5. HRMS analyses

Figure S16. HRMS analysis of isolated intact CP probe.

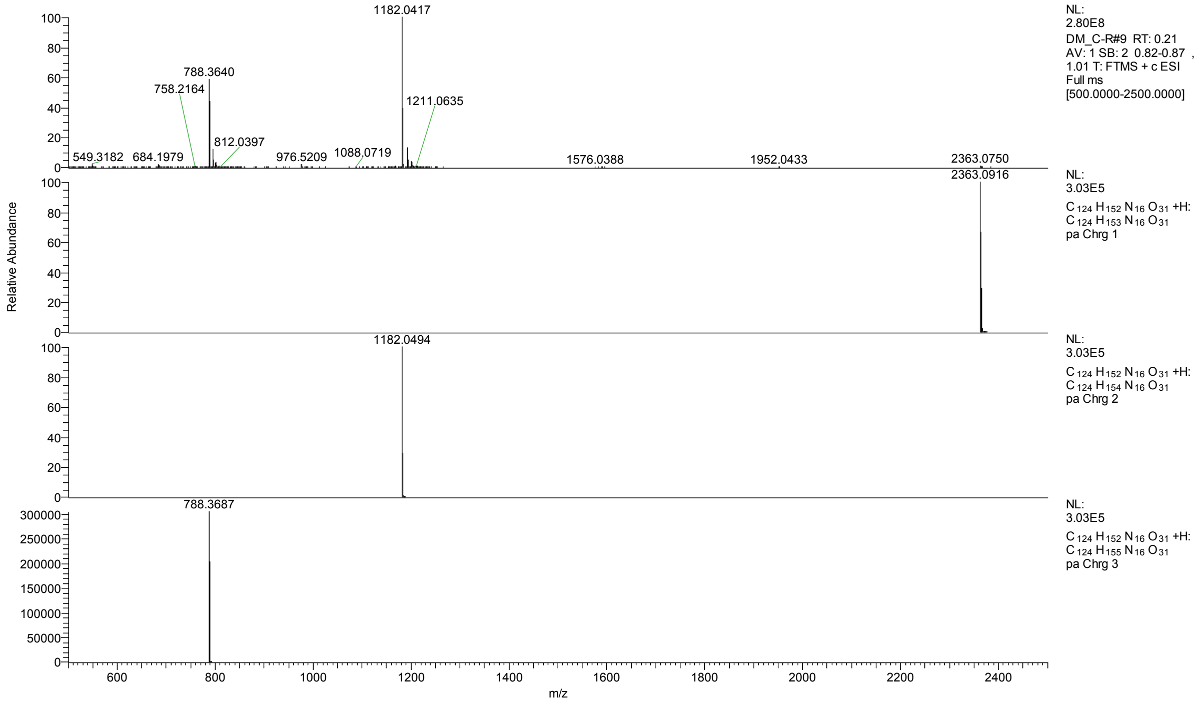

HRMS (ESI):

m/z calcd $C_{124}H_{153}N_{16}O_{31}^{+}$ for $[M+H]^{+}$ = 2363.0916, found $[M+H]^{+}$ = 2363.0750;

m/z calcd $C_{124}H_{153}N_{16}O_{31}^{+}$ for $[M+2H]^{2+}$ = 1182.0494, found $[M+2H]^{2+}$ = 1182.0417;

m/z calcd $C_{124}H_{153}N_{16}O_{31}^{+}$ for $[M+3H]^{3+}$ = 788.3687, found $[M+3H]^{3+}$ = 788.3640.

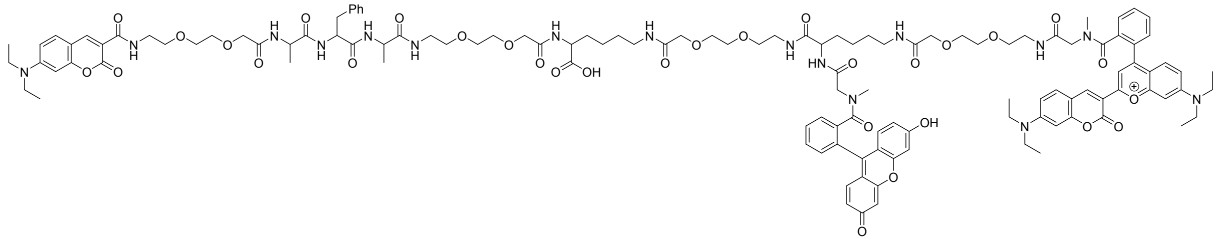

Figure S17. HRMS analysis of isolated intact C probe.

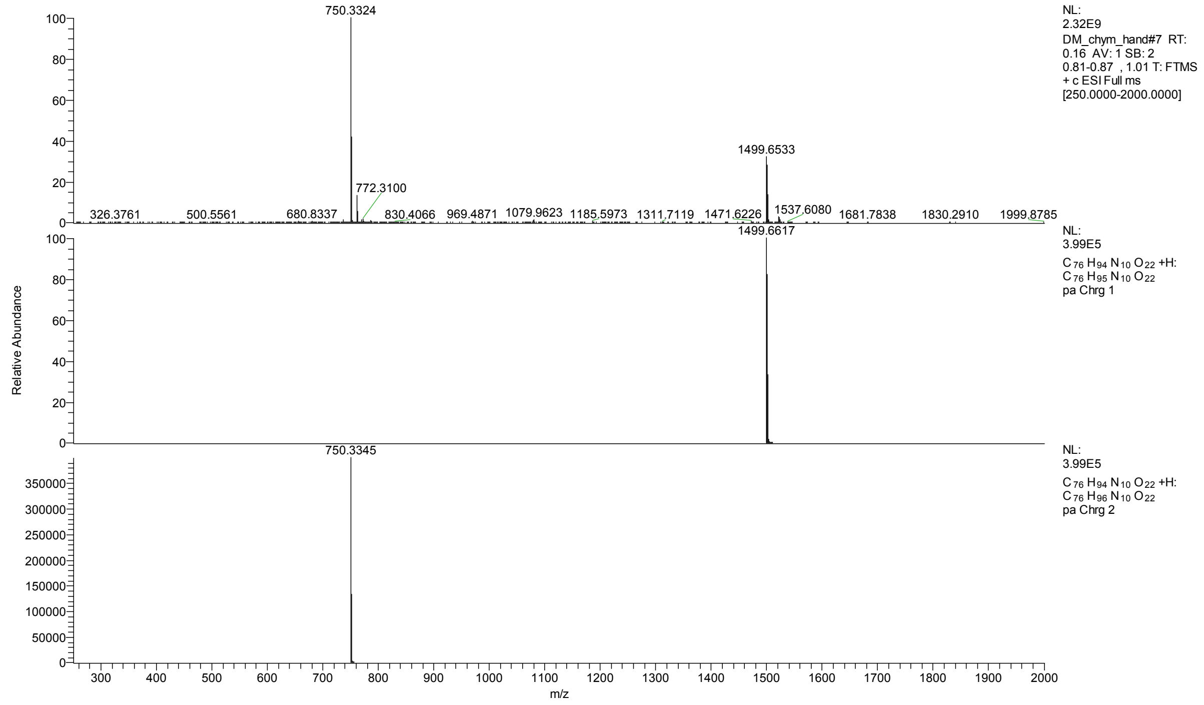

HRMS (ESI):
m/z calcd $C_{76}H_{94}N_{10}O_{22}$ for $[M+H]^{+}$ = 1499.6617, found $[M+H]^{+}$ = 1499.6533;
m/z calcd $C_{76}H_{94}N_{10}O_{22}$ for $[M+2H]^{2+}$ = 750.3345, found $[M+2H]^{2+}$ = 750.3324.

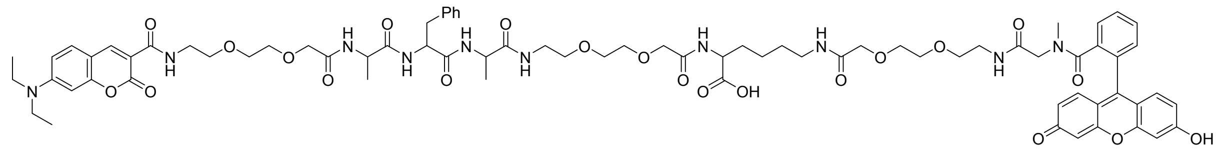

6. Fluorescence analyses

Table S1: Quantum yields of peptide-bound fluorophores – CP probe.

DEAC	FL	BC	Reference
<0.01	<0.01	<0.01	Fluorescein (0.1 M NaOH)
0.02	0.01	0.02	Rhodamine 6G (water)
0.01	<0.01	0.01	Rhodamine B (water)

Table S2. Time-dependent fluorescence response of CP probe – blank sample. The visualization of the corresponding numerical data can be found in the graph under the table.

Time [min]	DEAC Average	DEAC St. Dev.	FL Average	FL St. Dev.	BC Average	BC St. Dev.
0	11.38	0.05	9.70	0.10	8.53	0.11
5	11.46	0.23	10.11	0.08	8.37	0.15
10	11.53	0.02	10.36	0.06	8.54	0.10
15	11.64	0.09	10.54	0.11	8.60	0.11
20	11.67	0.17	11.02	0.32	8.44	0.11
25	11.80	0.22	11.09	0.20	8.33	0.12
30	11.83	0.33	11.28	0.20	8.53	0.14
35	11.78	0.21	11.56	0.22	8.46	0.08
40	12.00	0.14	11.69	0.20	8.51	0.12
45	12.10	0.29	12.04	0.43	8.45	0.10

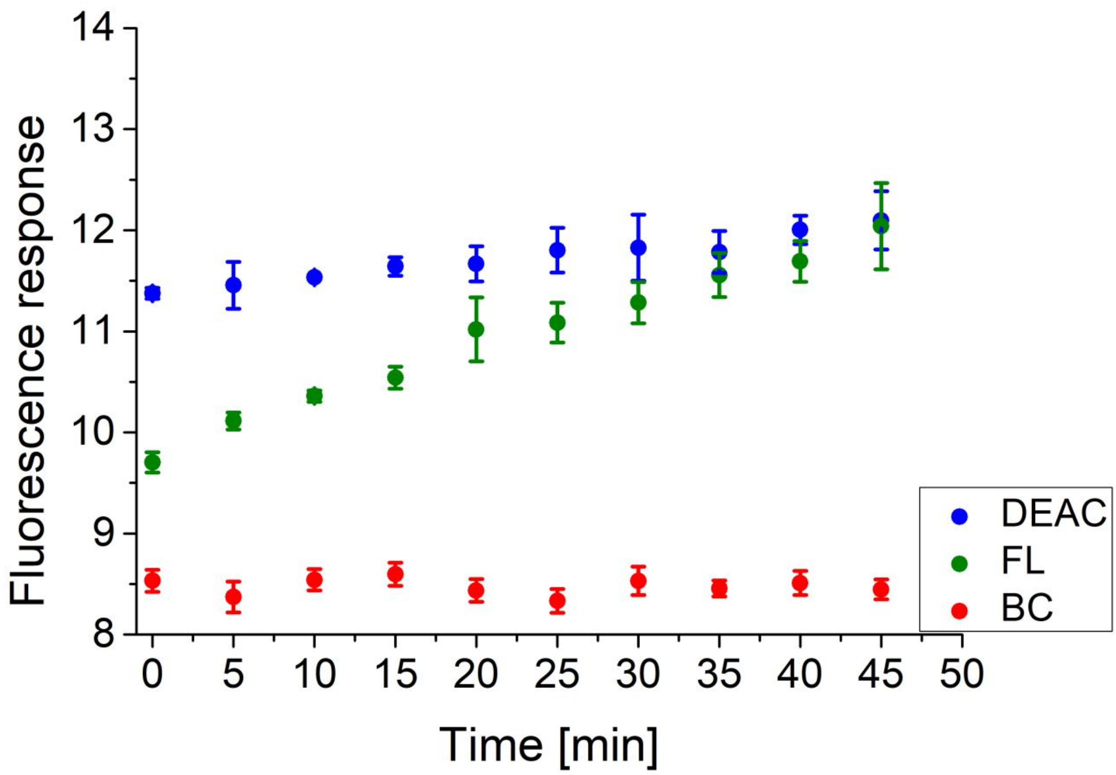

Preparation: 10 µM CP probe in DMSO (10 µL) and Tris buffer (970 µL) (0 min), then addition of ultrapure water (10 µL) and 1 mM HCl (10 µL) (0–45 min); Incubation: T=37 °C; Excitation: λ=425 nm; Emissions: λ_{DEAC}=477 nm, λ_{FL}=529 nm, λ_{BC}=722 nm; $Slit_{EXC}/Slit_{EMS}$=10/10 nm. All measurements were performed in three parallels. The corresponding average values and standard deviations are reported.

Table S3. Time-dependent fluorescence response of CP probe – detection of chymotrypsin (c=50 ng/mL). The visualization of the corresponding numerical data can be found in the graph under the table.

Time [min]	DEAC Average	DEAC St. Dev.	FL Average	FL St. Dev.	BC Average	BC St. Dev.
0	12.34	0.36	9.59	0.38	8.28	0.10
5	12.49	0.01	10.50	0.35	8.42	0.10
10	12.77	0.21	10.58	0.56	8.29	0.17
15	13.09	0.04	10.91	0.33	8.22	0.24
20	13.35	0.14	11.30	0.25	8.31	0.08
25	13.71	0.06	11.63	0.61	8.30	0.05
30	14.10	0.11	11.95	0.38	8.50	0.20
35	14.22	0.16	12.13	0.61	8.29	0.09
40	14.51	0.06	12.50	0.55	8.34	0.08
45	14.99	0.30	12.89	0.53	8.28	0.15

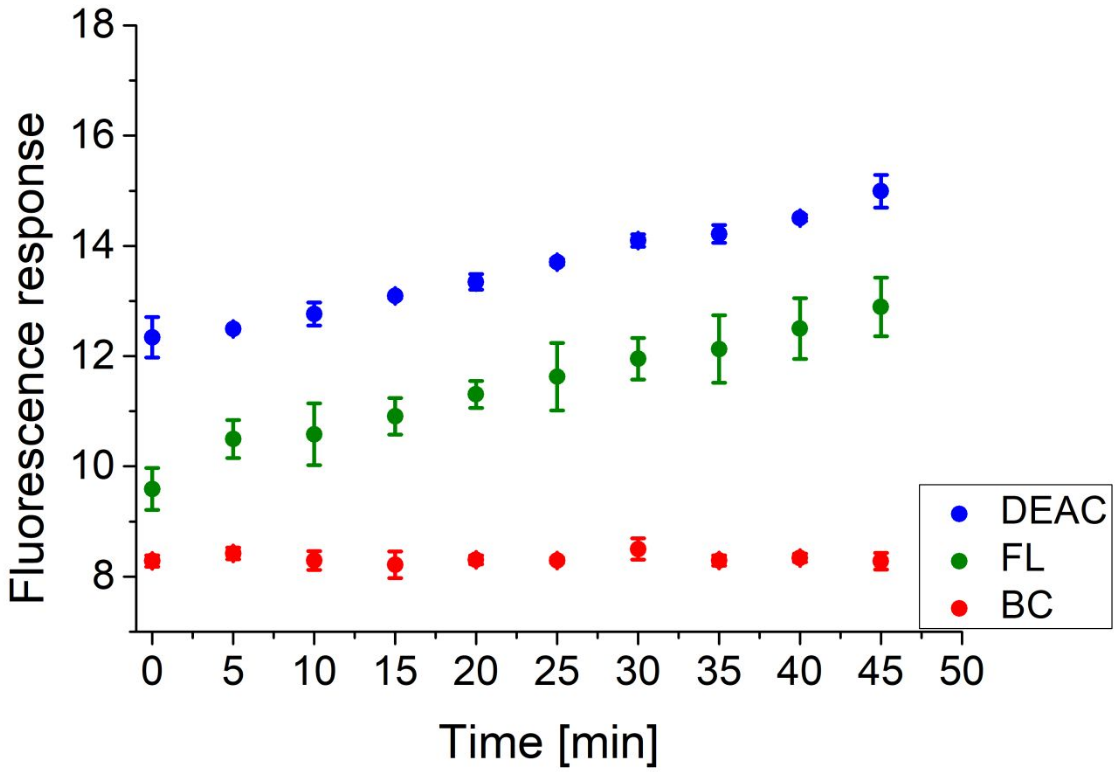

Preparation: 10 µM CP probe in DMSO (10 µL) and Tris buffer (970 µL) (0 min), then addition of ultrapure water (10 µL) and 1 mM HCl (10 µL) with chymotrypsin (50 ng) (0–45 min); Incubation: T=37 °C; Excitation: λ=425 nm; Emissions: λ_{DEAC}=477 nm, λ_{FL}=529 nm, λ_{BC}=722 nm; $Slit_{EXC}/Slit_{EMS}$=10/10 nm. All measurements were performed in three parallels. The corresponding average values and standard deviations are reported.

Table S4. Time-dependent fluorescence response of CP probe – detection of chymotrypsin (c=100 ng/mL). The visualization of the corresponding numerical data can be found in the graph under the table.

Time [min]	DEAC Average	DEAC St. Dev.	FL Average	FL St. Dev.	BC Average	BC St. Dev.
0	11.93	0.40	11.00	0.53	8.39	0.52
5	12.71	0.53	11.75	0.19	8.68	0.47
10	13.56	0.36	12.27	0.03	8.53	0.37
15	14.18	0.52	12.65	0.24	8.47	0.43
20	15.10	0.57	13.13	0.33	8.50	0.42
25	15.53	0.59	13.51	0.15	8.45	0.52
30	16.33	0.90	14.27	0.20	8.55	0.34
35	17.16	0.97	14.60	0.35	8.53	0.32
40	17.73	0.56	15.02	0.12	8.47	0.40
45	18.74	0.82	15.47	0.05	8.44	0.42

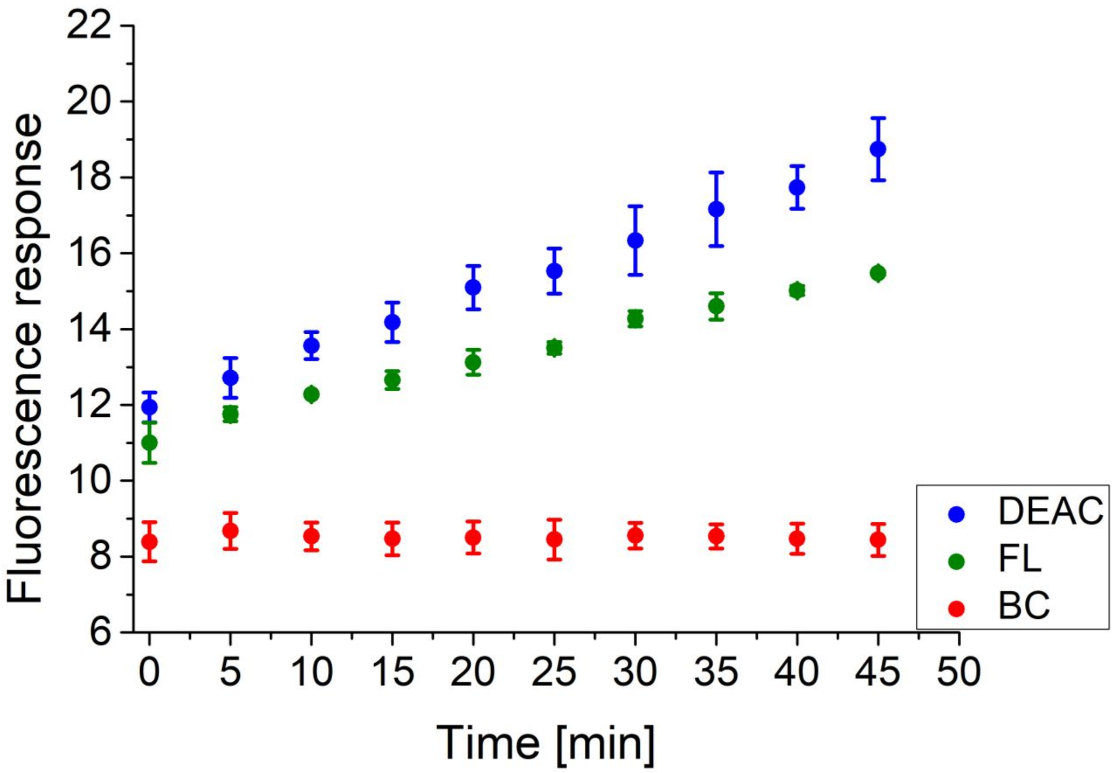

Preparation: 10 µM CP probe in DMSO (10 µL) and Tris buffer (970 µL) (0 min), then addition of ultrapure water (10 µL) and 1 mM HCl (10 µL) with chymotrypsin (100 ng) (0–45 min); Incubation: T=37 °C; Excitation: λ=425 nm; Emissions: $λ_{DEAC}$=477 nm, $λ_{FL}$=529 nm, $λ_{BC}$=722 nm; $Slit_{EXC}$/$Slit_{EMS}$=10/10 nm. All measurements were performed in three parallels. The corresponding average values and standard deviations are reported.

Table S5. Time-dependent fluorescence response of CP probe – detection of chymotrypsin (c=200 ng/mL). The visualization of the corresponding numerical data can be found in the graph under the table.

Time [min]	DEAC Average	DEAC St. Dev.	FL Average	FL St. Dev.	BC Average	BC St. Dev.
0	12.29	0.17	9.83	0.08	8.43	0.16
5	13.44	0.50	10.68	0.23	8.53	0.23
10	14.46	0.16	11.13	0.09	8.32	0.19
15	15.44	0.42	11.72	0.35	8.50	0.14
20	16.38	0.30	12.26	0.24	8.51	0.19
25	17.52	0.51	12.80	0.40	8.42	0.19
30	18.70	0.66	13.32	0.30	8.41	0.02
35	19.95	0.79	14.19	0.20	8.40	0.14
40	20.91	0.75	14.86	0.57	8.50	0.16
45	21.90	0.74	15.23	0.39	8.41	0.22

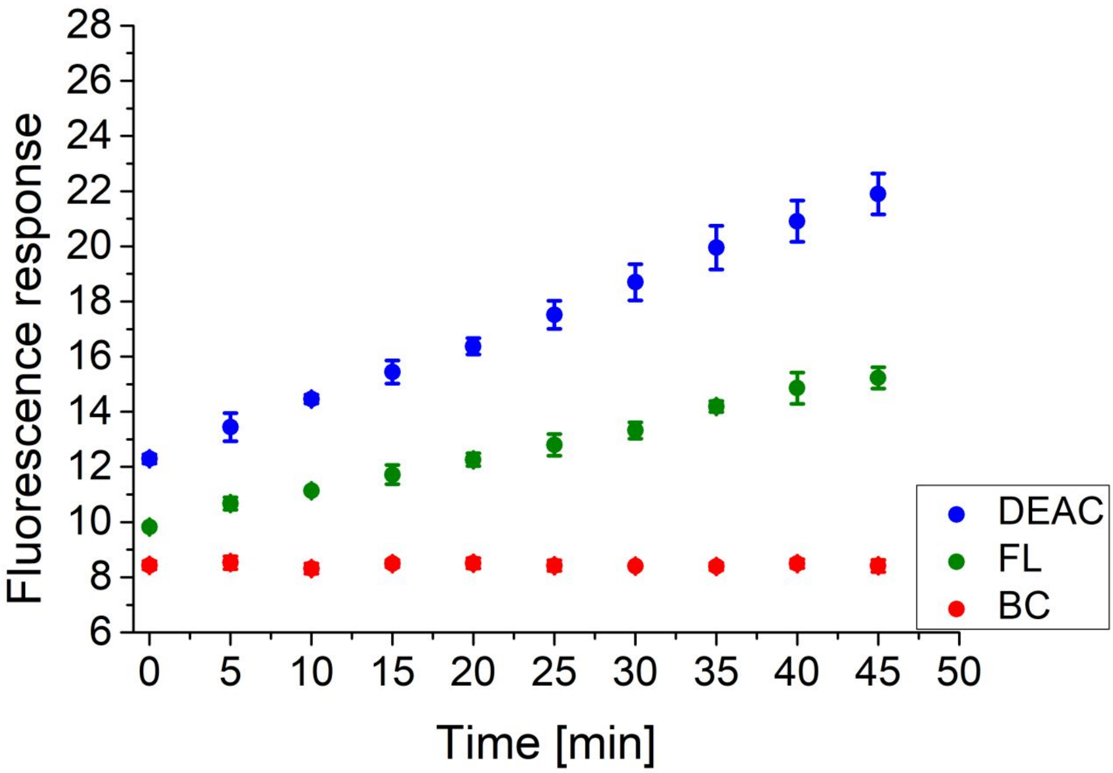

Preparation: 10 µM CP probe in DMSO (10 µL) and Tris buffer (970 µL) (0 min), then addition of ultrapure water (10 µL) and 1 mM HCl (10 µL) with chymotrypsin (200 ng) (0–45 min); Incubation: T=37 °C; Excitation: λ=425 nm; Emissions: λ_{DEAC}=477 nm, λ_{FL}=529 nm, λ_{BC}=722 nm; $Slit_{EXC}/Slit_{EMS}$=10/10 nm. All measurements were performed in three parallels. The corresponding average values and standard deviations are reported.

Table S6. Time-dependent fluorescence response of CP probe – detection of chymotrypsin (c=500 ng/mL). The visualization of the corresponding numerical data can be found in the graph under the table.

Time [min]	DEAC Average	DEAC St. Dev.	FL Average	FL St. Dev.	BC Average	BC St. Dev.
0	12.08	0.33	10.16	0.93	8.41	0.24
5	14.32	0.45	11.07	0.71	8.36	0.24
10	16.96	0.57	11.90	0.78	8.20	0.16
15	19.37	0.47	13.20	0.74	8.33	0.21
20	22.06	0.62	14.22	0.96	8.19	0.17
25	24.27	0.85	15.15	0.88	8.29	0.08
30	26.70	0.92	16.26	1.08	8.39	0.23
35	29.40	1.28	16.92	1.06	8.04	0.24
40	31.87	1.21	18.21	1.07	8.31	0.29
45	34.67	1.30	19.15	0.93	8.17	0.19

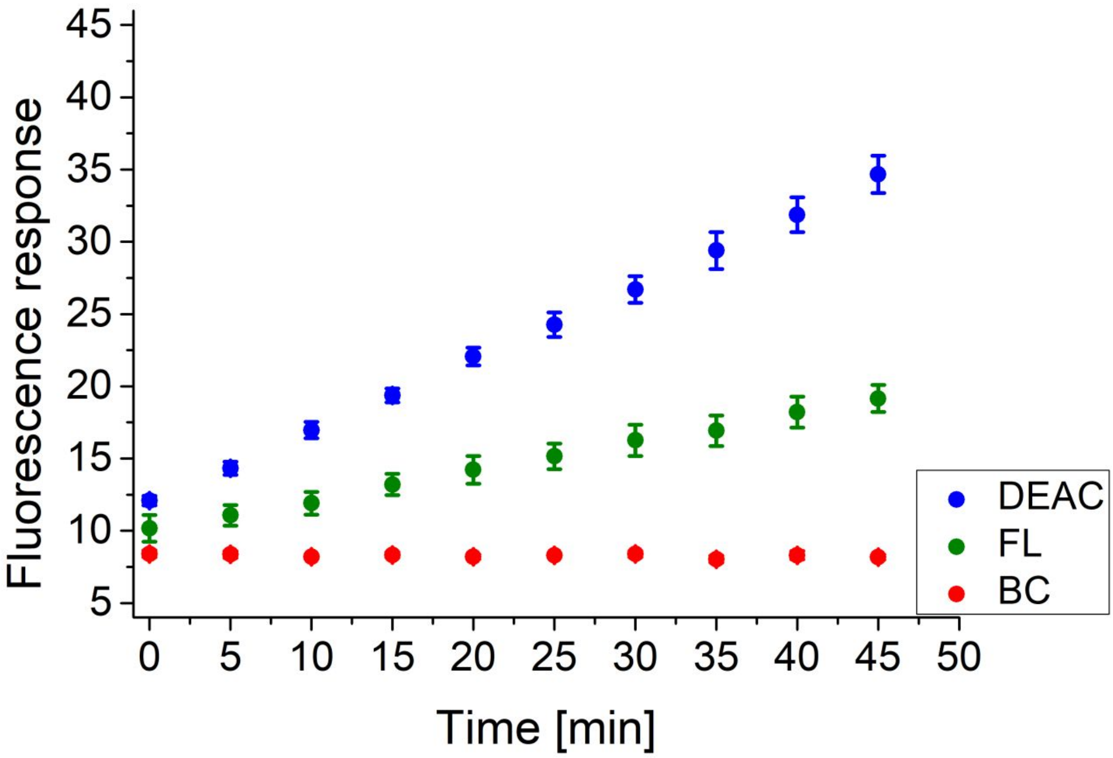

Preparation: 10 µM CP probe in DMSO (10 µL) and Tris buffer (970 µL) (0 min), then addition of ultrapure water (10 µL) and 1 mM HCl (10 µL) with chymotrypsin (500 ng) (0–45 min); Incubation: T=37 °C; Excitation: λ=425 nm; Emissions: λ_{DEAC}=477 nm, λ_{FL}=529 nm, λ_{BC}=722 nm; $Slit_{EXC}$/$Slit_{EMS}$=10/10 nm. All measurements were performed in three parallels. The corresponding average values and standard deviations are reported.

Table S7. Time-dependent fluorescence response of CP probe – detection of chymotrypsin (c=1 µg/mL). The visualization of the corresponding numerical data can be found in the graph under the table.

Time [min]	DEAC Average	DEAC St. Dev.	FL Average	FL St. Dev.	BC Average	BC St. Dev.
0	12.11	0.09	9.68	0.30	8.48	0.21
5	16.98	0.59	11.77	0.46	8.36	0.10
10	21.91	0.65	13.53	0.43	8.30	0.23
15	27.10	1.04	15.31	0.66	8.39	0.27
20	32.07	1.24	17.23	0.74	8.31	0.28
25	37.21	1.58	18.87	0.66	8.24	0.25
30	41.88	1.83	20.67	0.87	8.17	0.00
35	46.96	2.01	22.70	1.02	8.30	0.19
40	52.00	2.45	24.44	1.06	8.14	0.26
45	57.02	2.26	26.21	1.05	8.16	0.34

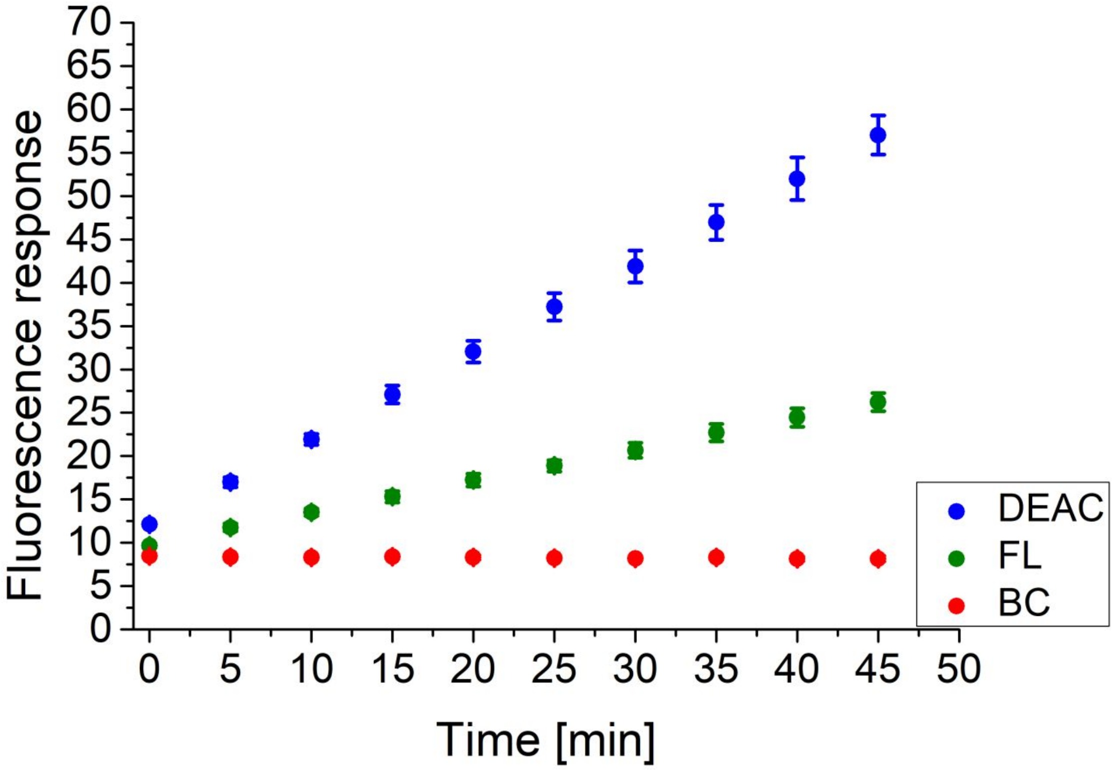

Preparation: 10 µM CP probe in DMSO (10 µL) and Tris buffer (970 µL) (0 min), then addition of ultrapure water (10 µL) and 1 mM HCl (10 µL) with chymotrypsin (1 µg) (0–45 min); Incubation: T=37 °C; Excitation: λ=425 nm; Emissions: λ_{DEAC}=477 nm, λ_{FL}=529 nm, λ_{BC}=722 nm; $Slit_{EXC}/Slit_{EMS}$=10/10 nm. All measurements were performed in three parallels. The corresponding average values and standard deviations are reported.

Table S8. Time-dependent fluorescence response of CP probe – detection of hydrogen peroxide (c=10 µM). The visualization of the corresponding numerical data can be found in the graph under the table.

Time [min]	DEAC Average	DEAC St. Dev.	FL Average	FL St. Dev.	BC Average	BC St. Dev.
0	12.35	0.03	9.80	0.20	8.71	0.17
5	12.53	0.19	10.37	0.08	8.60	0.16
10	12.68	0.13	11.04	0.17	8.78	0.14
15	12.43	0.17	11.23	0.10	8.62	0.04
20	12.72	0.08	12.03	0.11	8.66	0.09
25	13.01	0.40	12.42	0.27	8.74	0.17
30	12.74	0.21	12.81	0.26	8.74	0.11
35	12.89	0.08	13.50	0.18	8.58	0.14
40	13.12	0.15	14.02	0.32	8.64	0.09
45	13.10	0.06	14.43	0.21	8.66	0.08

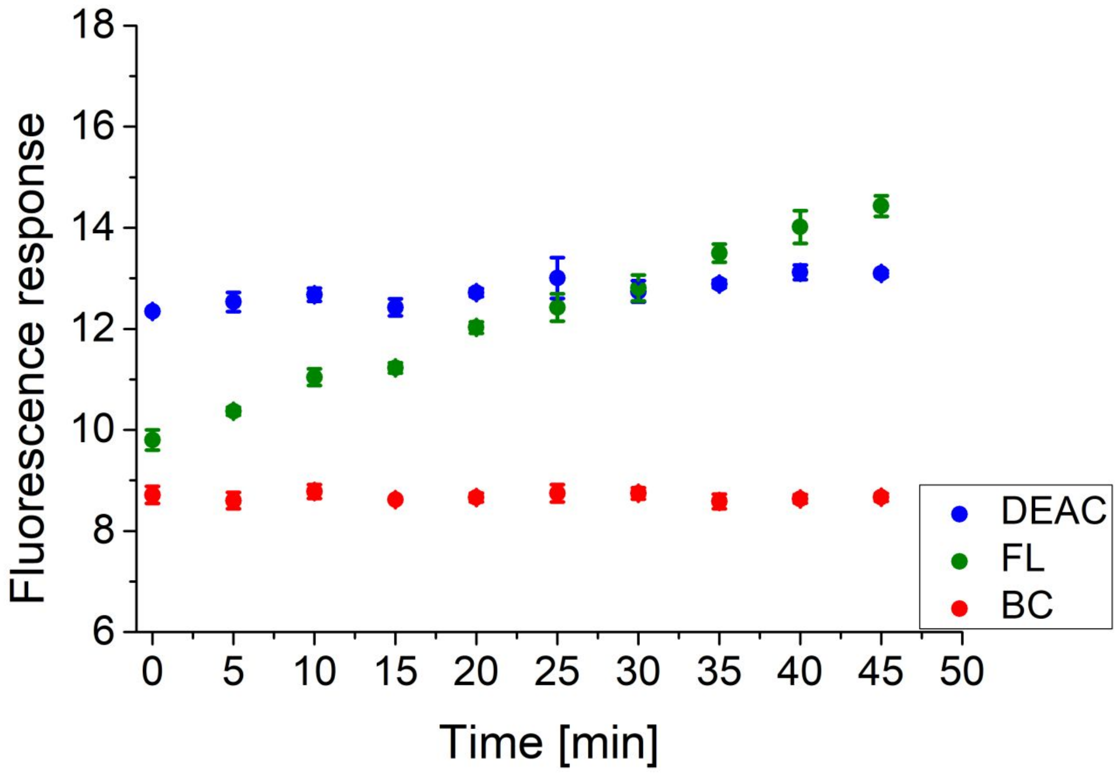

Preparation: 10 µM CP probe in DMSO (10 µL) and Tris buffer (970 µL) (0 min), then addition of ultrapure water (10 µL) with H_2O_2 (1 mM) and 1 mM HCl (10 µL) (0–45 min); Incubation: T=37 °C; Excitation: λ=425 nm; Emissions: λ_{DEAC}=477 nm, λ_{FL}=529 nm, λ_{BC}=722 nm; $Slit_{EXC}/Slit_{EMS}$=10/10 nm. All measurements were performed in three parallels. The corresponding average values and standard deviations are reported.

Table S9. Time-dependent fluorescence response of CP probe – detection of hydrogen peroxide (c=20 µM). The visualization of the corresponding numerical data can be found in the graph under the table.

Time [min]	DEAC Average	DEAC St. Dev.	FL Average	FL St. Dev.	BC Average	BC St. Dev.
0	12.22	0.02	9.89	0.16	8.75	0.06
5	12.66	0.03	10.99	0.31	8.64	0.06
10	12.62	0.16	11.70	0.32	8.74	0.15
15	12.85	0.19	12.38	0.28	8.66	0.05
20	13.02	0.29	13.02	0.10	8.75	0.06
25	13.13	0.18	14.04	0.05	8.61	0.15
30	13.21	0.25	14.78	0.14	8.56	0.13
35	13.41	0.25	15.68	0.35	8.54	0.03
40	13.65	0.25	16.45	0.19	8.67	0.10
45	13.81	0.24	17.27	0.21	8.65	0.14

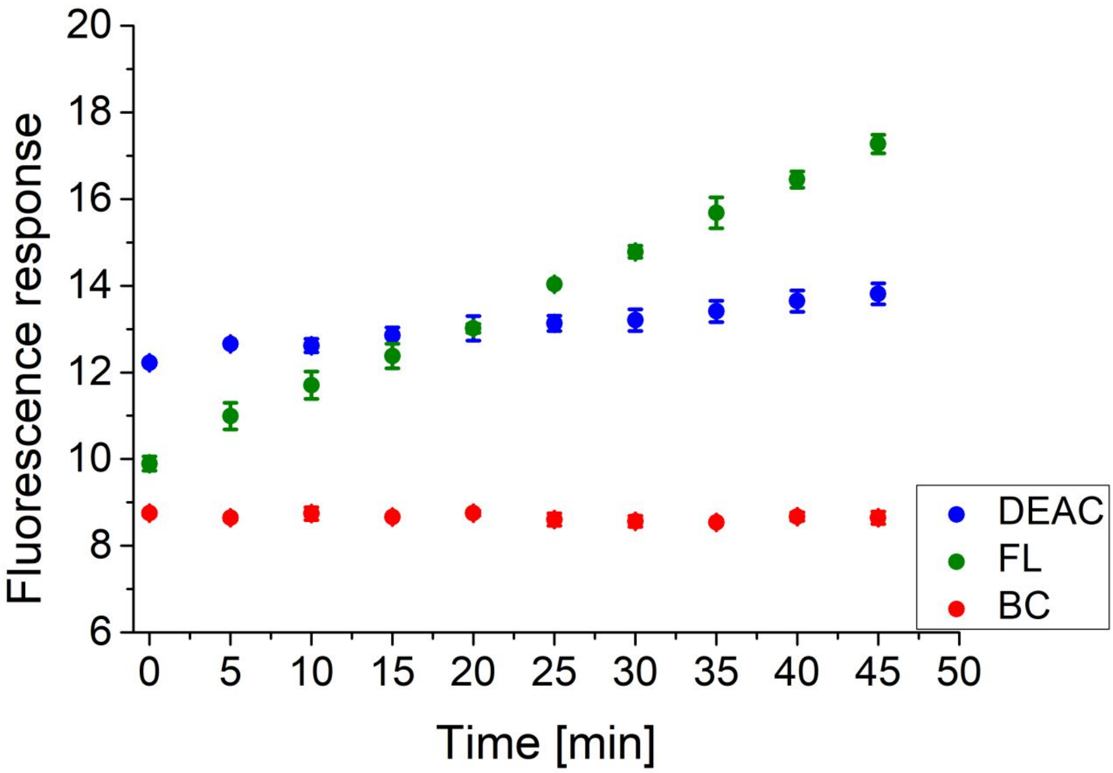

Preparation: 10 µM CP probe in DMSO (10 µL) and Tris buffer (970 µL) (0 min), then addition of ultrapure water (10 µL) with H_2O_2 (2 mM) and 1 mM HCl (10 µL) (0–45 min); Incubation: T=37 °C; Excitation: λ=425 nm; Emissions: λ_{DEAC}=477 nm, λ_{FL}=529 nm, λ_{BC}=722 nm; $Slit_{EXC}/Slit_{EMS}$=10/10 nm. All measurements were performed in three parallels. The corresponding average values and standard deviations are reported.

Table S10. Time-dependent fluorescence response of CP probe – detection of hydrogen peroxide (c=50 µM). The visualization of the corresponding numerical data can be found in the graph under the table.

Time [min]	DEAC Average	DEAC St. Dev.	FL Average	FL St. Dev.	BC Average	BC St. Dev.
0	11.82	0.36	9.96	0.28	8.86	0.38
5	12.11	0.27	11.30	0.46	8.88	0.31
10	12.38	0.36	12.58	0.48	9.01	0.34
15	12.66	0.40	14.24	0.46	8.83	0.14
20	12.90	0.12	15.80	0.47	8.74	0.34
25	13.08	0.35	17.34	0.60	8.77	0.22
30	13.63	0.27	19.16	0.54	8.69	0.23
35	13.71	0.16	20.86	0.52	8.68	0.15
40	14.09	0.37	22.81	0.78	8.60	0.29
45	14.47	0.41	24.58	0.90	8.56	0.12

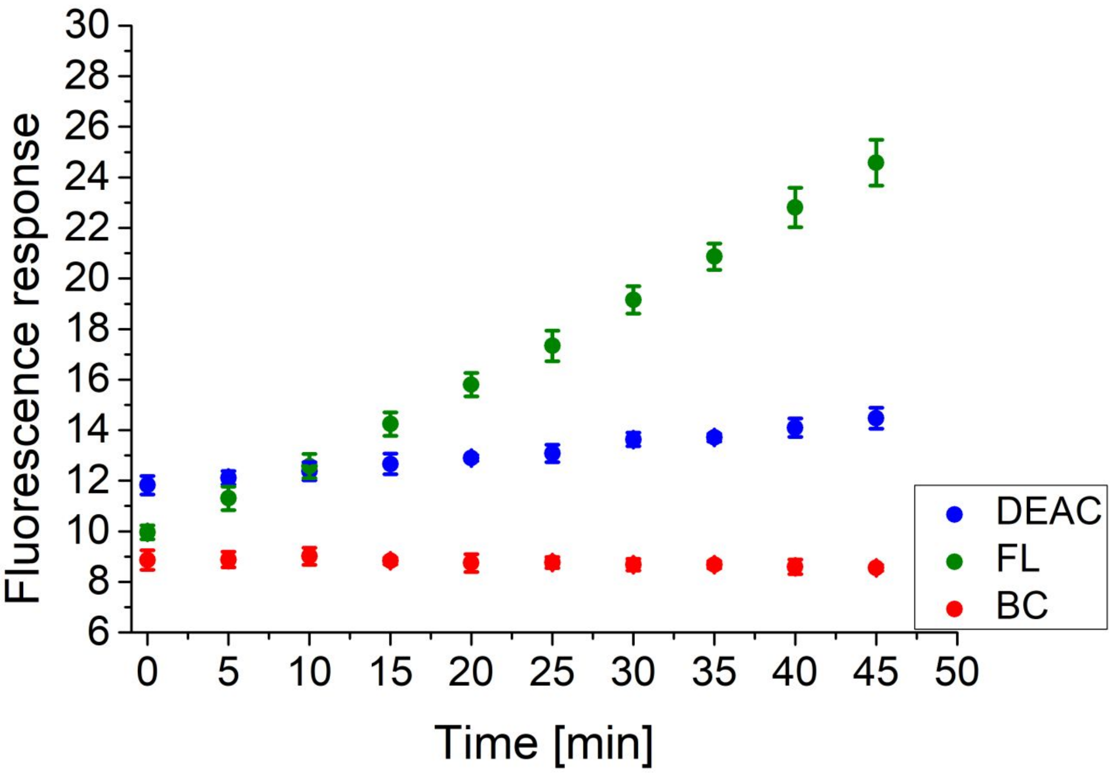

Preparation: 10 µM CP probe in DMSO (10 µL) and Tris buffer (970 µL) (0 min), then addition of ultrapure water (10 µL) with H_2O_2 (5 mM) and 1 mM HCl (10 µL) (0–45 min); Incubation: T=37 °C; Excitation: λ=425 nm; Emissions: λ_{DEAC}=477 nm, λ_{FL}=529 nm, λ_{BC}=722 nm; $Slit_{EXC}/Slit_{EMS}$=10/10 nm. All measurements were performed in three parallels. The corresponding average values and standard deviations are reported.

Table S11. Time-dependent fluorescence response of CP probe – detection of hydrogen peroxide (c=100 μM). The visualization of the corresponding numerical data can be found in the graph under the table.

Time [min]	DEAC Average	DEAC St. Dev.	FL Average	FL St. Dev.	BC Average	BC St. Dev.
0	11.68	0.15	10.02	0.01	8.70	0.32
5	12.25	0.38	12.33	0.27	8.61	0.35
10	12.62	0.40	14.96	0.04	8.42	0.35
15	13.12	0.28	17.77	0.29	8.45	0.35
20	13.78	0.29	20.58	0.40	8.41	0.33
25	14.21	0.35	23.48	0.55	8.35	0.28
30	14.87	0.16	26.83	0.39	8.41	0.26
35	15.50	0.19	30.87	0.85	8.34	0.14
40	15.82	0.56	33.96	0.77	8.17	0.27
45	16.37	0.32	37.82	1.12	8.04	0.34

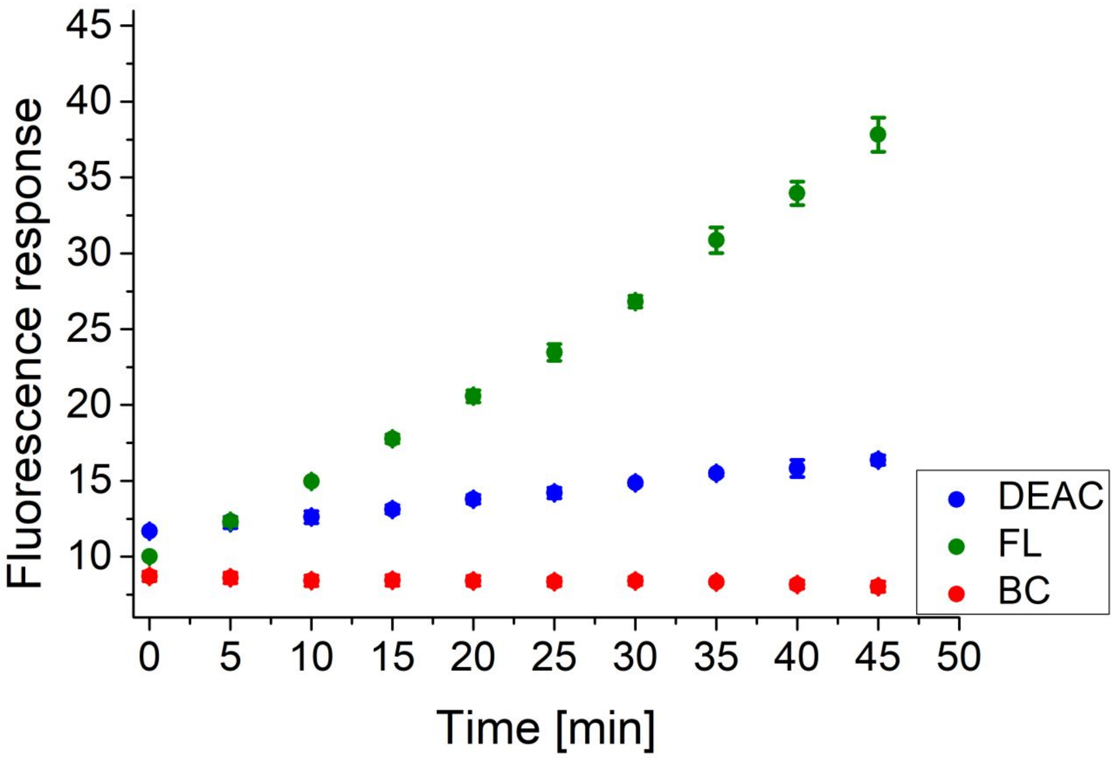

Preparation: 10 μM CP probe in DMSO (10 μL) and Tris buffer (970 μL) (0 min), then addition of ultrapure water (10 μL) with H_2O_2 (10 mM) and 1 mM HCl (10 μL) (0–45 min); Incubation: T=37 °C; Excitation: λ=425 nm; Emissions: λ_{DEAC}=477 nm, λ_{FL}=529 nm, λ_{BC}=722 nm; $Slit_{EXC}/Slit_{EMS}$=10/10 nm. All measurements were performed in three parallels. The corresponding average values and standard deviations are reported.

Table S12. Time-dependent fluorescence response of CP probe – detection of hydrogen peroxide (c=200 μM). The visualization of the corresponding numerical data can be found in the graph under the table.

Time [min]	DEAC Average	DEAC St. Dev.	FL Average	FL St. Dev.	BC Average	BC St. Dev.
0	11.45	0.23	9.32	0.07	8.61	0.36
5	12.33	0.15	13.86	0.09	8.40	0.14
10	13.19	0.32	18.56	0.09	8.26	0.15
15	14.11	0.15	23.82	0.14	8.31	0.26
20	15.08	0.39	29.35	0.18	8.22	0.16
25	16.09	0.24	35.47	0.28	8.10	0.26
30	17.27	0.42	41.64	0.32	7.89	0.21
35	18.00	0.44	47.90	0.64	8.01	0.10
40	19.10	0.27	55.46	0.88	7.71	0.21
45	20.11	0.43	62.40	0.97	7.53	0.13

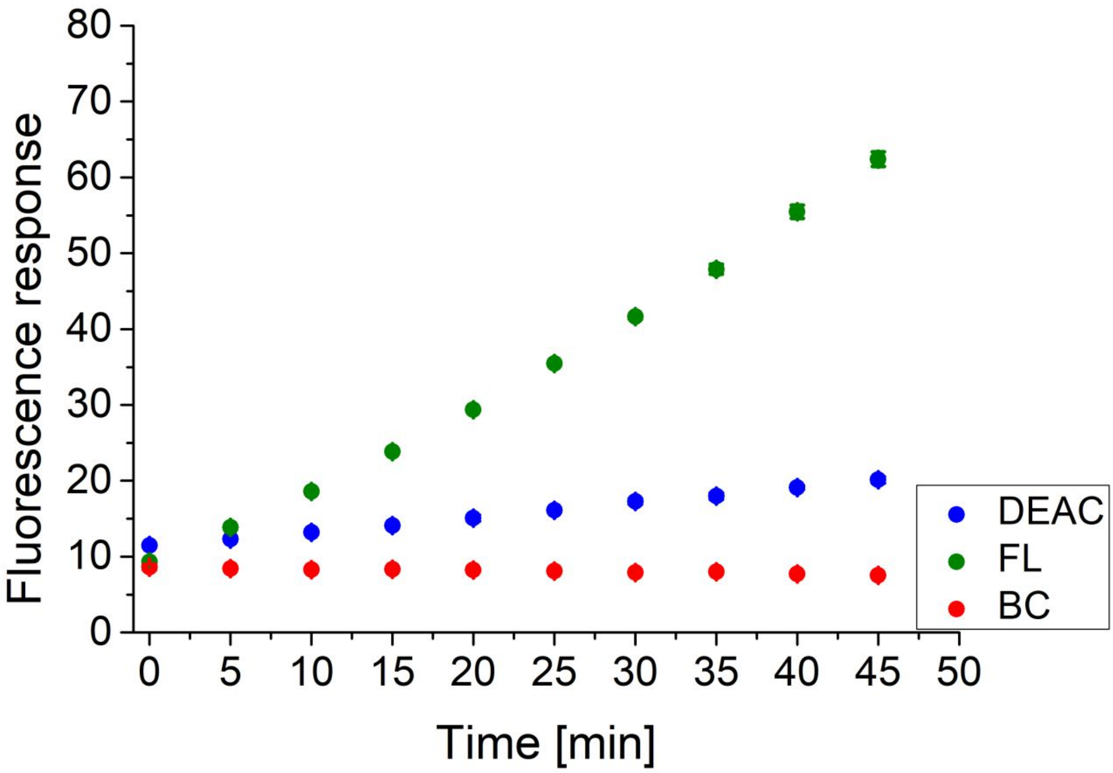

Preparation: 10 μM CP probe in DMSO (10 μL) and Tris buffer (970 μL) (0 min), then addition of ultrapure water (10 μL) with H_2O_2 (20 mM) and 1 mM HCl (10 μL) (0–45 min); Incubation: T=37 °C; Excitation: λ=425 nm; Emissions: λ_{DEAC}=477 nm, λ_{FL}=529 nm, λ_{BC}=722 nm; $Slit_{EXC}/Slit_{EMS}$=10/10 nm. All measurements were performed in three parallels. The corresponding average values and standard deviations are reported.

Table S13. Time-dependent fluorescence response of CP probe – detection of chymotrypsin (100 ng/mL) and hydrogen peroxide (c=20 µM). The visualization of the corresponding numerical data can be found in the graph under the table.

Time [min]	DEAC Average	DEAC St. Dev.	FL Average	FL St. Dev.	BC Average	BC St. Dev.
0	11.72	0.13	9.68	0.09	8.80	0.09
5	12.22	0.16	10.61	0.12	8.59	0.07
10	12.82	0.12	11.37	0.03	8.51	0.23
15	13.75	0.24	12.26	0.13	8.35	0.07
20	14.41	0.24	13.21	0.10	8.45	0.09
25	15.09	0.14	14.23	0.18	8.30	0.04
30	15.76	0.35	15.04	0.15	8.41	0.10
35	16.43	0.37	16.06	0.09	8.35	0.19
40	17.05	0.56	17.36	0.12	8.50	0.03
45	17.72	0.32	18.27	0.14	8.47	0.06

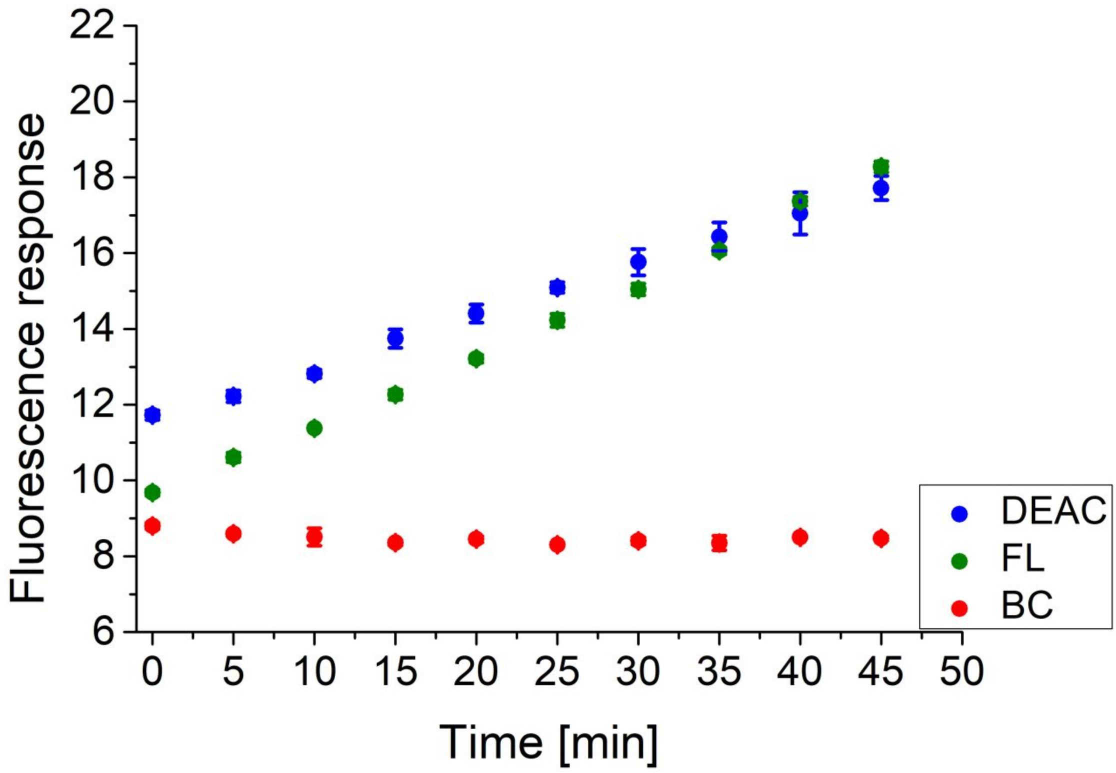

Preparation: 10 µM CP probe in DMSO (10 µL) and Tris buffer (970 µL) (0 min), then addition of ultrapure water (10 µL) with H_2O_2 (2 mM) and 1 mM HCl (10 µL) with chymotrypsin (100 ng) (0–45 min); Incubation: T=37 °C; Excitation: λ=425 nm; Emissions: λ_{DEAC}=477 nm, λ_{FL}=529 nm, λ_{BC}=722 nm; $Slit_{EXC}/Slit_{EMS}$=10/10 nm. All measurements were performed in three parallels. The corresponding average values and standard deviations are reported.

Table S14. Time-dependent fluorescence response of CP probe – detection of chymotrypsin (100 ng/mL) and hydrogen peroxide (c=50 µM). The visualization of the corresponding numerical data can be found in the graph under the table.

Time [min]	DEAC Average	DEAC St. Dev.	FL Average	FL St. Dev.	BC Average	BC St. Dev.
0	11.38	0.12	9.75	0.10	8.64	0.06
5	12.17	0.15	11.08	0.30	8.44	0.01
10	12.94	0.13	12.62	0.43	8.60	0.06
15	13.82	0.05	14.31	0.42	8.43	0.12
20	14.49	0.15	15.88	0.44	8.41	0.08
25	15.15	0.30	17.81	0.58	8.49	0.07
30	16.24	0.25	19.51	0.46	8.45	0.04
35	17.05	0.23	21.04	0.35	8.43	0.20
40	17.94	0.36	23.13	0.59	8.37	0.07
45	18.71	0.11	25.57	0.73	8.38	0.08

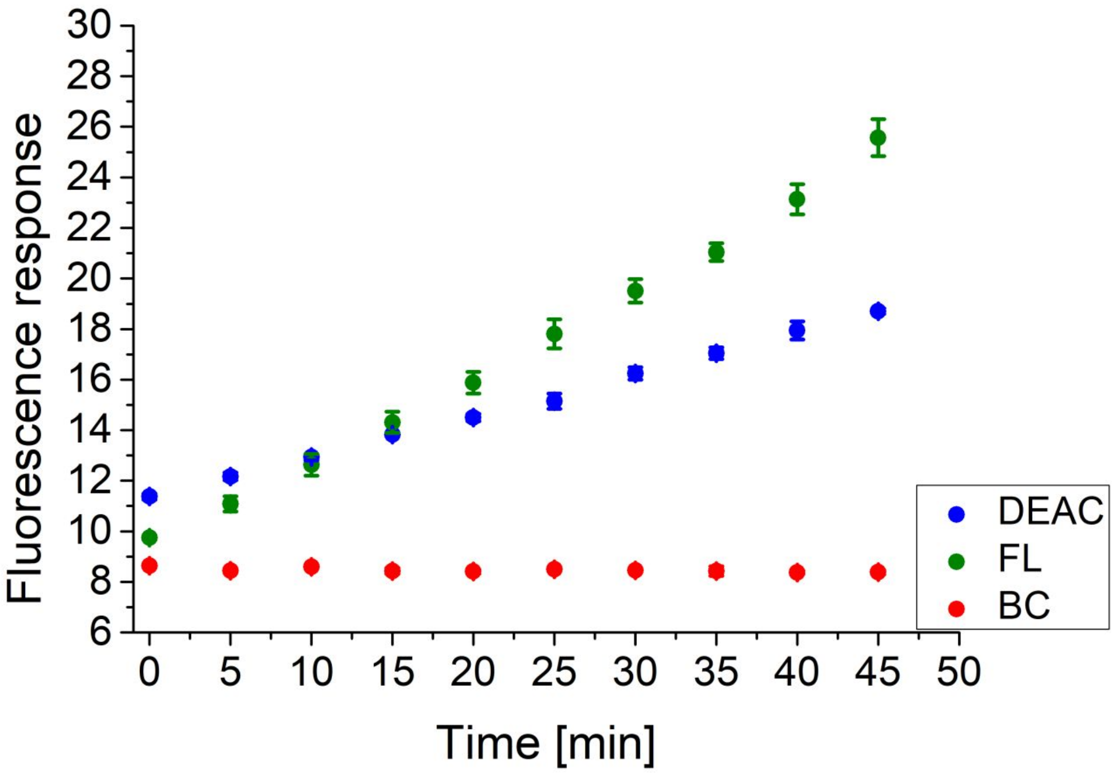

Preparation: 10 µM CP probe in DMSO (10 µL) and Tris buffer (970 µL) (0 min), then addition of ultrapure water (10 µL) with H_2O_2 (5 mM) and 1 mM HCl (10 µL) with chymotrypsin (100 ng) (0–45 min); Incubation: T=37 °C; Excitation: λ=425 nm; Emissions: λ_{DEAC}=477 nm, λ_{FL}=529 nm, λ_{BC}=722 nm; $Slit_{EXC}/Slit_{EMS}$=10/10 nm. All measurements were performed in three parallels. The corresponding average values and standard deviations are reported.

Table S15. Time-dependent fluorescence response of CP probe – detection of chymotrypsin (100 ng/mL) and hydrogen peroxide (c=100 µM). The visualization of the corresponding numerical data can be found in the graph under the table.

Time [min]	DEAC Average	DEAC St. Dev.	FL Average	FL St. Dev.	BC Average	BC St. Dev.
0	11.51	0.06	10.04	0.23	8.40	0.14
5	12.45	0.23	12.43	0.45	8.37	0.03
10	13.29	0.28	15.24	0.58	8.29	0.07
15	14.50	0.47	17.91	0.84	8.18	0.13
20	15.37	0.41	21.01	0.75	8.17	0.18
25	16.54	0.50	24.20	0.88	7.99	0.18
30	17.61	0.50	27.54	0.95	8.01	0.15
35	18.74	0.67	31.16	1.08	7.91	0.15
40	19.73	0.57	34.91	0.90	7.97	0.07
45	20.71	0.61	38.43	1.44	7.87	0.03

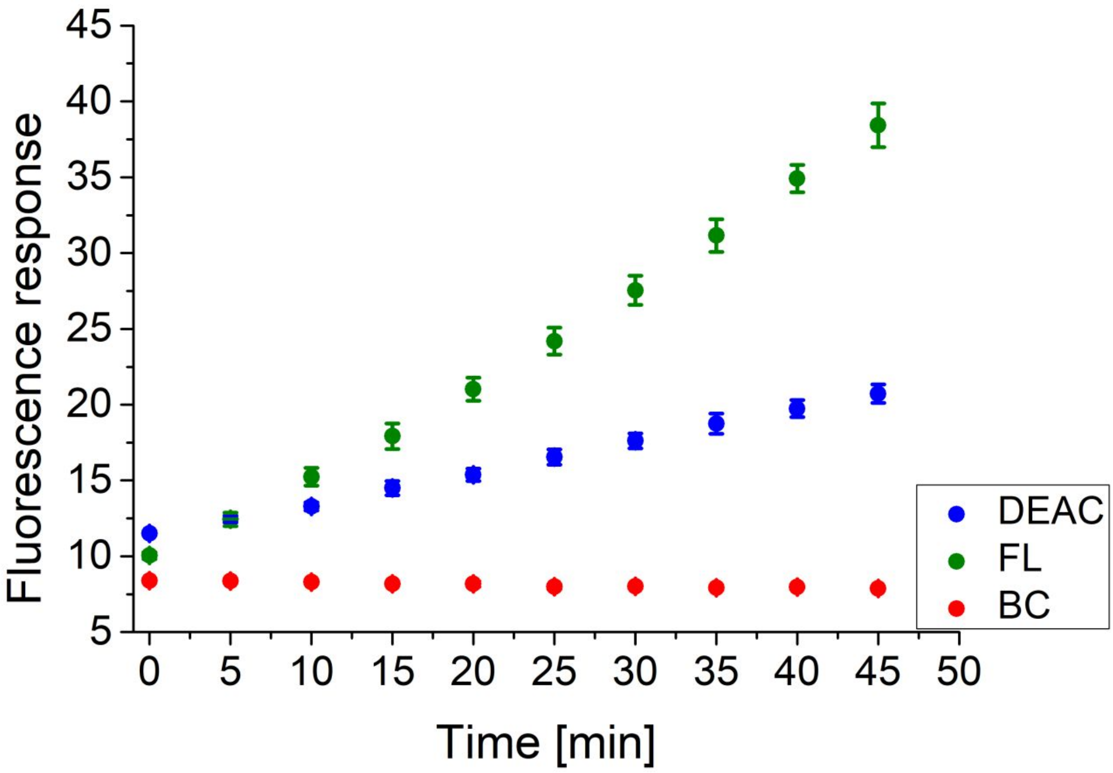

Preparation: 10 µM CP probe in DMSO (10 µL) and Tris buffer (970 µL) (0 min), then addition of ultrapure water (10 µL) with H_2O_2 (10 mM) and 1 mM HCl (10 µL) with chymotrypsin (100 ng) (0–45 min); Incubation: T=37 °C; Excitation: λ=425 nm; Emissions: λ_{DEAC}=477 nm, λ_{FL}=529 nm, λ_{BC}=722 nm; $Slit_{EXC}/Slit_{EMS}$=10/10 nm. All measurements were performed in three parallels. The corresponding average values and standard deviations are reported.

Table S16. Time-dependent fluorescence response of CP probe – detection of chymotrypsin (100 ng/mL) and hydrogen peroxide (c=200 µM). The visualization of the corresponding numerical data can be found in the graph under the table.

Time [min]	DEAC Average	DEAC St. Dev.	FL Average	FL St. Dev.	BC Average	BC St. Dev.
0	11.30	0.23	9.95	0.29	8.72	0.17
5	12.71	0.41	14.13	0.75	8.50	0.48
10	14.24	0.41	19.09	0.82	8.23	0.33
15	15.47	0.39	24.21	1.12	8.31	0.35
20	17.16	0.46	29.77	1.11	8.08	0.24
25	18.39	0.50	35.81	1.54	8.07	0.28
30	19.85	0.57	42.00	1.77	7.82	0.30
35	21.76	0.56	48.29	1.91	7.74	0.24
40	23.09	0.63	55.00	2.44	7.71	0.27
45	24.72	0.63	61.62	2.45	7.53	0.31

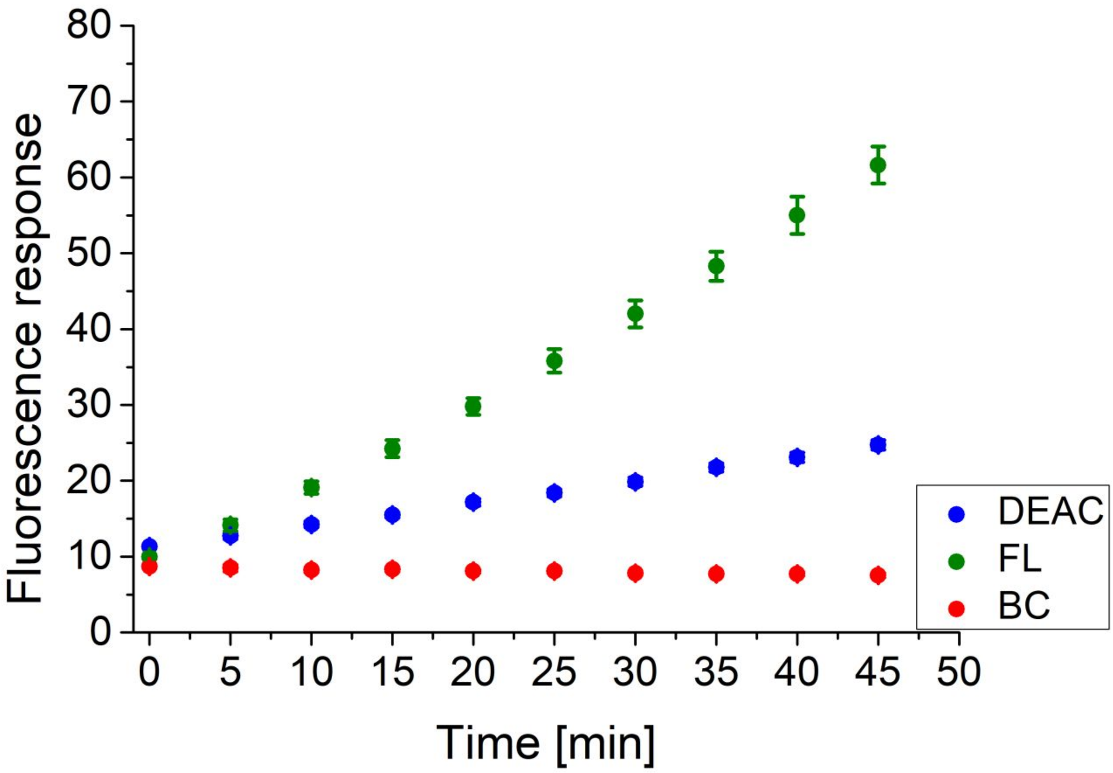

Preparation: 10 µM CP probe in DMSO (10 µL) and Tris buffer (970 µL) (0 min), then addition of ultrapure water (10 µL) with H_2O_2 (20 mM) and 1 mM HCl (10 µL) with chymotrypsin (100 ng) (0–45 min); Incubation: T=37 °C; Excitation: λ=425 nm; Emissions: λ_{DEAC}=477 nm, λ_{FL}=529 nm, λ_{BC}=722 nm; $Slit_{EXC}/Slit_{EMS}$=10/10 nm. All measurements were performed in three parallels. The corresponding average values and standard deviations are reported.

Table S17. Time-dependent fluorescence response of CP probe – detection of chymotrypsin (200 ng/mL) and hydrogen peroxide (c=20 µM). The visualization of the corresponding numerical data can be found in the graph under the table.

Time [min]	DEAC Average	DEAC St. Dev.	FL Average	FL St. Dev.	BC Average	BC St. Dev.
0	11.54	0.10	10.24	0.01	8.73	0.18
5	12.82	0.12	11.38	0.07	8.65	0.07
10	14.28	0.29	12.34	0.14	8.68	0.02
15	15.54	0.47	13.27	0.12	8.64	0.23
20	16.56	0.38	14.63	0.23	8.50	0.11
25	18.05	0.27	15.60	0.34	8.55	0.07
30	19.14	0.45	16.77	0.17	8.54	0.05
35	20.39	0.44	17.62	0.13	8.41	0.21
40	21.85	0.57	19.18	0.26	8.48	0.04
45	23.08	0.73	20.16	0.15	8.52	0.04

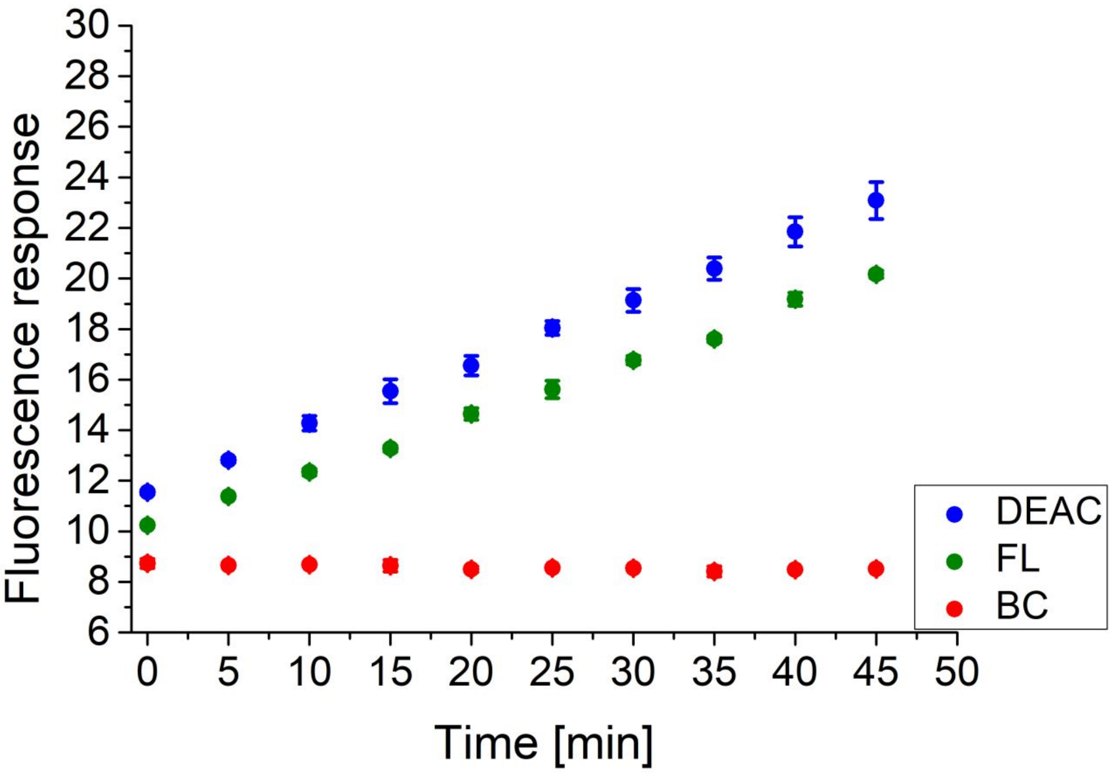

Preparation: 10 µM CP probe in DMSO (10 µL) and Tris buffer (970 µL) (0 min), then addition of ultrapure water (10 µL) with H_2O_2 (2 mM) and 1 mM HCl (10 µL) with chymotrypsin (200 ng) (0–45 min); Incubation: T=37 °C; Excitation: λ=425 nm; Emissions: λ_{DEAC}=477 nm, λ_{FL}=529 nm, λ_{BC}=722 nm; $Slit_{EXC}/Slit_{EMS}$=10/10 nm. All measurements were performed in three parallels. The corresponding average values and standard deviations are reported.

Table S18. Time-dependent fluorescence response of CP probe – detection of chymotrypsin (200 ng/mL) and hydrogen peroxide (c=50 µM). The visualization of the corresponding numerical data can be found in the graph under the table.

Time [min]	DEAC Average	DEAC St. Dev.	FL Average	FL St. Dev.	BC Average	BC St. Dev.
0	11.10	0.41	9.45	0.15	8.80	0.17
5	12.64	0.35	11.23	0.19	8.54	0.09
10	13.88	0.36	12.78	0.21	8.57	0.10
15	15.27	0.17	14.54	0.07	8.52	0.01
20	16.99	0.42	16.24	0.22	8.38	0.09
25	18.39	0.29	18.32	0.32	8.46	0.05
30	19.63	0.36	20.15	0.27	8.35	0.12
35	21.05	0.45	22.48	0.36	8.36	0.17
40	22.40	0.53	24.70	0.51	8.18	0.15
45	24.57	0.60	26.91	0.50	8.28	0.03

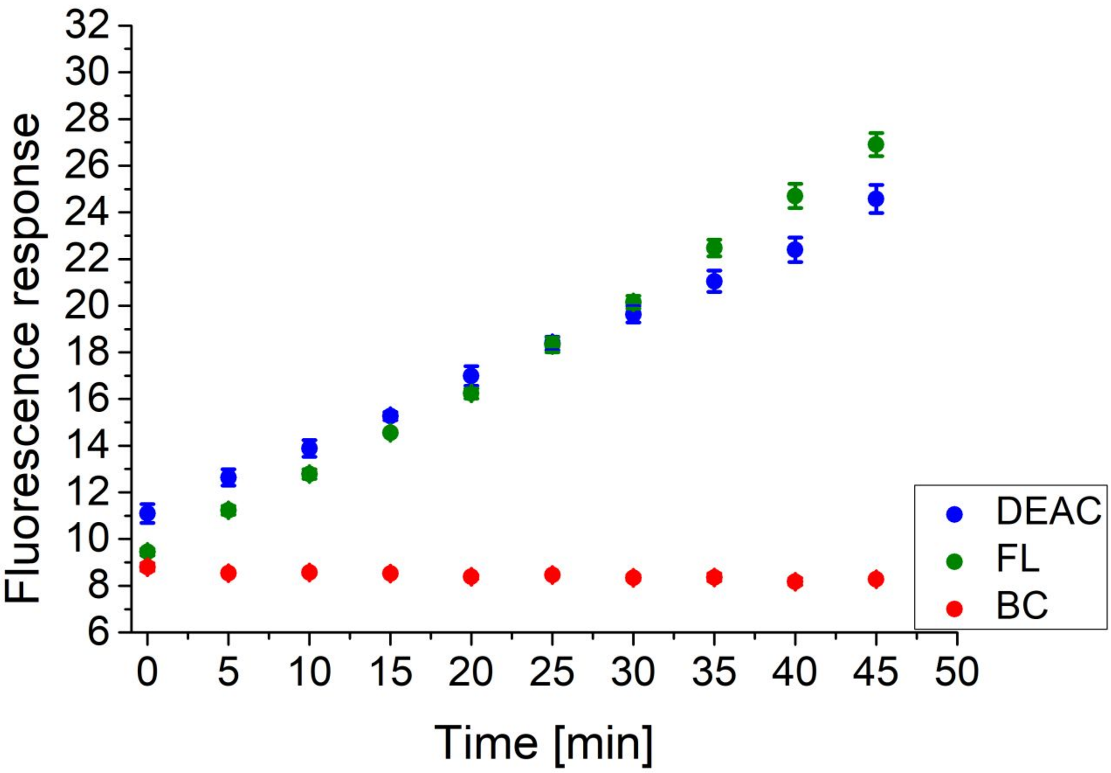

Preparation: 10 µM CP probe in DMSO (10 µL) and Tris buffer (970 µL) (0 min), then addition of ultrapure water (10 µL) with H_2O_2 (5 mM) and 1 mM HCl (10 µL) with chymotrypsin (200 ng) (0–45 min); Incubation: T=37 °C; Excitation: λ=425 nm; Emissions: λ_{DEAC}=477 nm, λ_{FL}=529 nm, λ_{BC}=722 nm; $Slit_{EXC}/Slit_{EMS}$=10/10 nm. All measurements were performed in three parallels. The corresponding average values and standard deviations are reported.

Table S19. Time-dependent fluorescence response of CP probe – detection of chymotrypsin (200 ng/mL) and hydrogen peroxide (c=100 µM). The visualization of the corresponding numerical data can be found in the graph under the table.

Time [min]	DEAC Average	DEAC St. Dev.	FL Average	FL St. Dev.	BC Average	BC St. Dev.
0	11.24	0.36	9.59	0.34	8.67	0.21
5	12.67	0.22	12.18	0.52	8.48	0.14
10	14.29	0.23	15.25	0.47	8.46	0.20
15	15.84	0.32	17.96	0.58	8.43	0.14
20	17.39	0.27	21.36	0.60	8.38	0.10
25	19.27	0.18	24.79	0.68	8.24	0.22
30	20.90	0.43	28.42	0.75	8.14	0.19
35	22.39	0.37	32.41	0.62	8.12	0.13
40	24.40	0.33	36.41	0.81	8.03	0.14
45	26.05	0.53	40.14	0.95	8.06	0.20

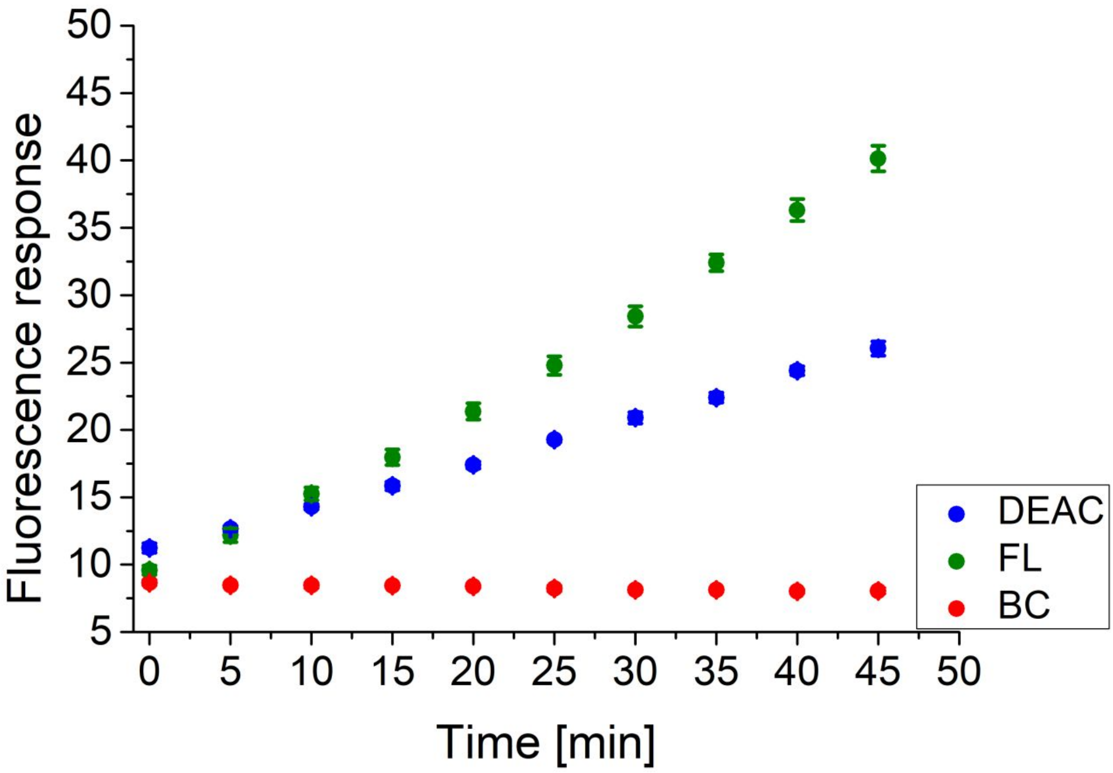

Preparation: 10 µM CP probe in DMSO (10 µL) and Tris buffer (970 µL) (0 min), then addition of ultrapure water (10 µL) with H_2O_2 (10 mM) and 1 mM HCl (10 µL) with chymotrypsin (200 ng) (0–45 min); Incubation: T=37 °C; Excitation: λ=425 nm; Emissions: λ_{DEAC}=477 nm, λ_{FL}=529 nm, λ_{BC}=722 nm; $Slit_{EXC}/Slit_{EMS}$=10/10 nm. All measurements were performed in three parallels. The corresponding average values and standard deviations are reported.

Table S20. Time-dependent fluorescence response of CP probe – detection of chymotrypsin (200 ng/mL) and hydrogen peroxide (c=200 µM). The visualization of the corresponding numerical data can be found in the graph under the table.

Time [min]	DEAC Average	DEAC St. Dev.	FL Average	FL St. Dev.	BC Average	BC St. Dev.
0	11.34	0.16	9.33	0.20	8.69	0.21
5	13.17	0.46	14.03	0.34	8.49	0.16
10	15.15	0.14	19.29	0.59	8.42	0.15
15	17.32	0.51	25.10	0.76	8.32	0.22
20	19.37	0.41	31.37	0.57	8.18	0.06
25	21.51	0.43	38.15	1.32	8.12	0.21
30	23.90	0.29	44.68	1.17	7.89	0.15
35	25.90	0.61	52.08	1.36	7.85	0.08
40	28.07	0.41	59.50	1.70	7.73	0.05
45	30.18	0.46	67.27	1.49	7.56	0.10

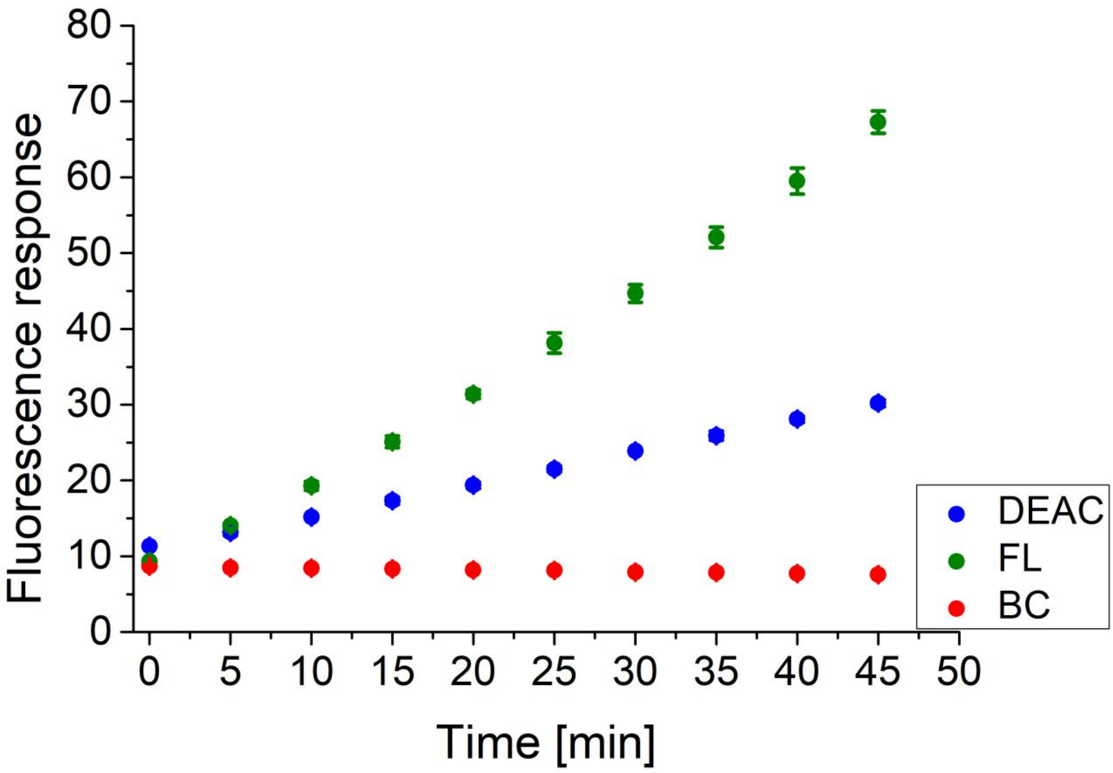

Preparation: 10 µM CP probe in DMSO (10 µL) and Tris buffer (970 µL) (0 min), then addition of ultrapure water (10 µL) with H_2O_2 (20 mM) and 1 mM HCl (10 µL) with chymotrypsin (200 ng) (0–45 min); Incubation: T=37 °C; Excitation: λ=425 nm; Emissions: λ_{DEAC}=477 nm, λ_{FL}=529 nm, λ_{BC}=722 nm; $Slit_{EXC}/Slit_{EMS}$=10/10 nm. All measurements were performed in three parallels. The corresponding average values and standard deviations are reported.

Table S21. Time-dependent fluorescence response of CP probe – detection of chymotrypsin (500 ng/mL) and hydrogen peroxide (c=20 µM). The visualization of the corresponding numerical data can be found in the graph under the table.

Time [min]	DEAC Average	DEAC St. Dev.	FL Average	FL St. Dev.	BC Average	BC St. Dev.
0	11.26	0.22	9.40	0.30	8.26	0.08
5	13.97	0.31	11.14	0.38	8.34	0.15
10	16.71	0.17	12.64	0.33	8.23	0.27
15	19.64	0.12	14.29	0.53	8.13	0.24
20	22.53	0.32	15.95	0.33	8.22	0.20
25	25.30	0.62	17.45	0.60	8.11	0.15
30	28.01	0.46	19.33	0.41	8.15	0.15
35	31.01	0.29	20.78	0.68	8.15	0.26
40	33.63	0.75	22.57	0.65	8.15	0.22
45	36.94	0.80	24.18	0.66	8.05	0.15

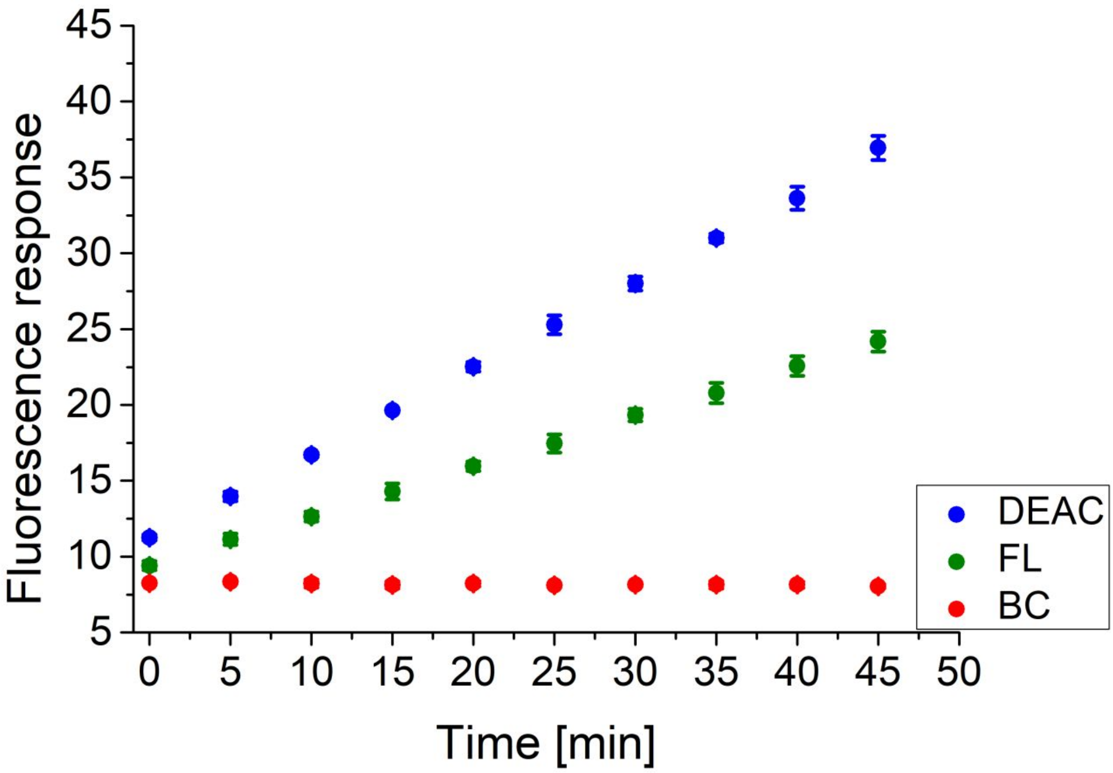

Preparation: 10 µM CP probe in DMSO (10 µL) and Tris buffer (970 µL) (0 min), then addition of ultrapure water (10 µL) with H_2O_2 (2 mM) and 1 mM HCl (10 µL) with chymotrypsin (500 ng) (0–45 min); Incubation: T=37 °C; Excitation: λ=425 nm; Emissions: λ_{DEAC}=477 nm, λ_{FL}=529 nm, λ_{BC}=722 nm; $Slit_{EXC}/Slit_{EMS}$=10/10 nm. All measurements were performed in three parallels. The corresponding average values and standard deviations are reported.

Table S22. Time-dependent fluorescence response of CP probe – detection of chymotrypsin (500 ng/mL) and hydrogen peroxide (c=50 μM). The visualization of the corresponding numerical data can be found in the graph under the table.

Time [min]	DEAC Average	DEAC St. Dev.	FL Average	FL St. Dev.	BC Average	BC St. Dev.
0	11.47	0.40	9.97	0.26	8.59	0.18
5	14.22	0.32	12.08	0.34	8.60	0.18
10	17.38	0.56	14.27	0.48	8.46	0.16
15	20.41	0.71	16.38	0.64	8.40	0.19
20	23.36	0.81	19.24	0.71	8.61	0.26
25	26.79	0.57	21.52	0.55	8.40	0.19
30	29.80	0.69	24.28	0.82	8.42	0.27
35	32.71	0.84	26.90	0.87	8.23	0.23
40	35.63	0.82	29.50	0.87	8.23	0.23
45	38.96	1.30	32.49	0.90	8.09	0.15

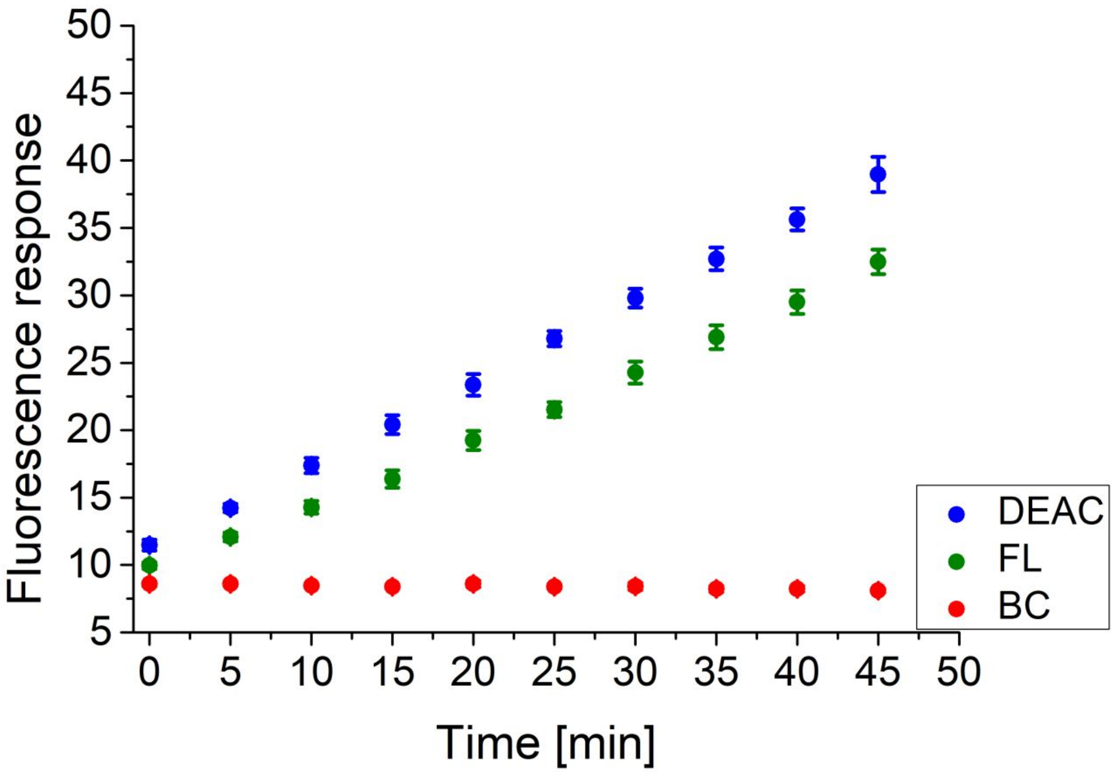

Preparation: 10 μM CP probe in DMSO (10 μL) and Tris buffer (970 μL) (0 min), then addition of ultrapure water (10 μL) with H_2O_2 (5 mM) and 1 mM HCl (10 μL) with chymotrypsin (500 ng) (0–45 min); Incubation: T=37 °C; Excitation: λ=425 nm; Emissions: λ_{DEAC}=477 nm, λ_{FL}=529 nm, λ_{BC}=722 nm; $Slit_{EXC}$/$Slit_{EMS}$=10/10 nm. All measurements were performed in three parallels. The corresponding average values and standard deviations are reported.

Table S23. Time-dependent fluorescence response of CP probe – detection of chymotrypsin (500 ng/mL) and hydrogen peroxide (c=100 μM). The visualization of the corresponding numerical data can be found in the graph under the table.

Time [min]	DEAC Average	DEAC St. Dev.	FL Average	FL St. Dev.	BC Average	BC St. Dev.
0	11.29	0.20	9.49	0.25	8.58	0.14
5	14.37	0.23	12.71	0.32	8.32	0.22
10	17.89	0.56	16.35	0.36	8.29	0.25
15	21.13	0.34	19.87	0.45	8.21	0.16
20	23.90	0.42	23.94	0.34	8.20	0.28
25	27.05	0.80	27.83	0.37	7.98	0.11
30	30.58	0.64	31.66	0.87	7.89	0.18
35	33.58	0.87	36.19	0.58	7.78	0.23
40	36.84	0.75	40.40	0.43	7.75	0.17
45	40.14	1.07	45.11	0.51	7.75	0.22

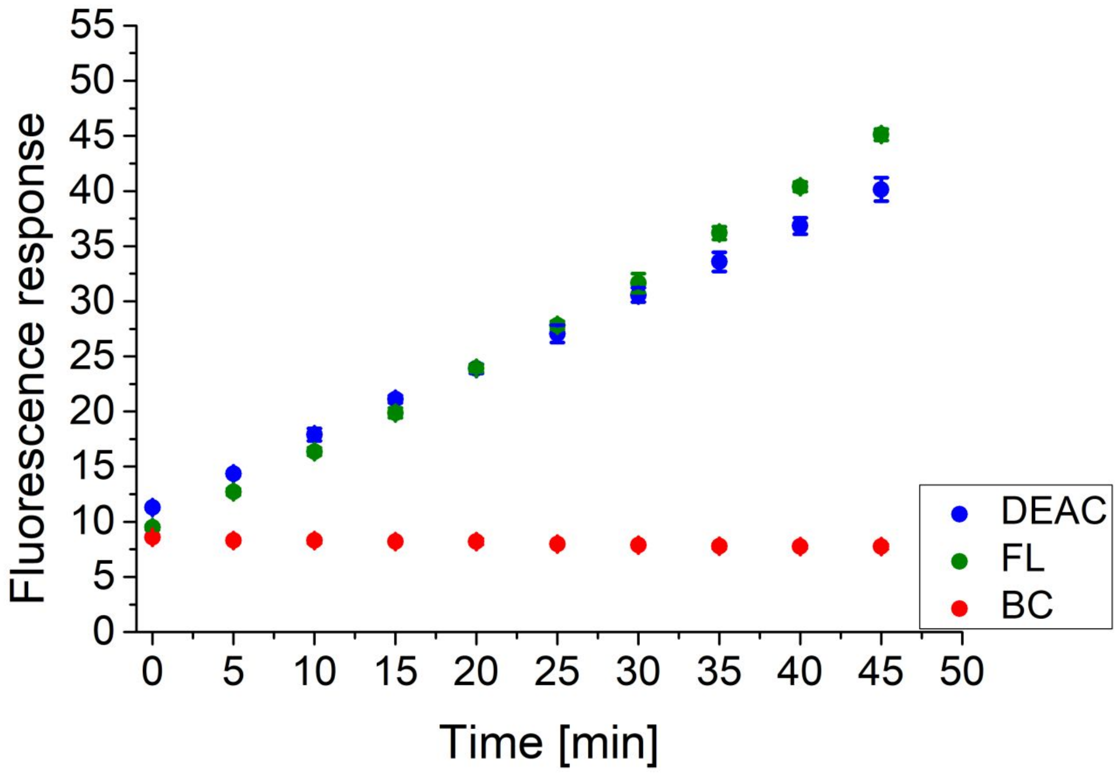

Preparation: 10 μM CP probe in DMSO (10 μL) and Tris buffer (970 μL) (0 min), then addition of ultrapure water (10 μL) with H_2O_2 (10 mM) and 1 mM HCl (10 μL) with chymotrypsin (500 ng) (0–45 min); Incubation: T=37 °C; Excitation: λ=425 nm; Emissions: λ_{DEAC}=477 nm, λ_{FL}=529 nm, λ_{BC}=722 nm; $Slit_{EXC}/Slit_{EMS}$=10/10 nm. All measurements were performed in three parallels. The corresponding average values and standard deviations are reported.

Table S24. Time-dependent fluorescence response of CP probe – detection of chymotrypsin (500 ng/mL) and hydrogen peroxide (c=200 µM). The visualization of the corresponding numerical data can be found in the graph under the table.

Time [min]	DEAC Average	DEAC St. Dev.	FL Average	FL St. Dev.	BC Average	BC St. Dev.
0	11.41	0.04	9.62	0.18	8.46	0.18
5	15.14	0.48	15.23	0.23	8.27	0.07
10	18.56	0.39	20.85	0.56	8.12	0.14
15	22.50	0.44	27.03	0.66	8.02	0.14
20	26.54	0.68	33.94	0.41	7.77	0.14
25	30.21	0.51	40.86	0.83	7.78	0.12
30	34.06	0.51	48.34	0.89	7.67	0.15
35	38.00	0.86	56.02	1.05	7.41	0.22
40	42.11	0.53	64.04	1.31	7.47	0.21
45	46.16	0.47	71.88	1.29	7.25	0.19

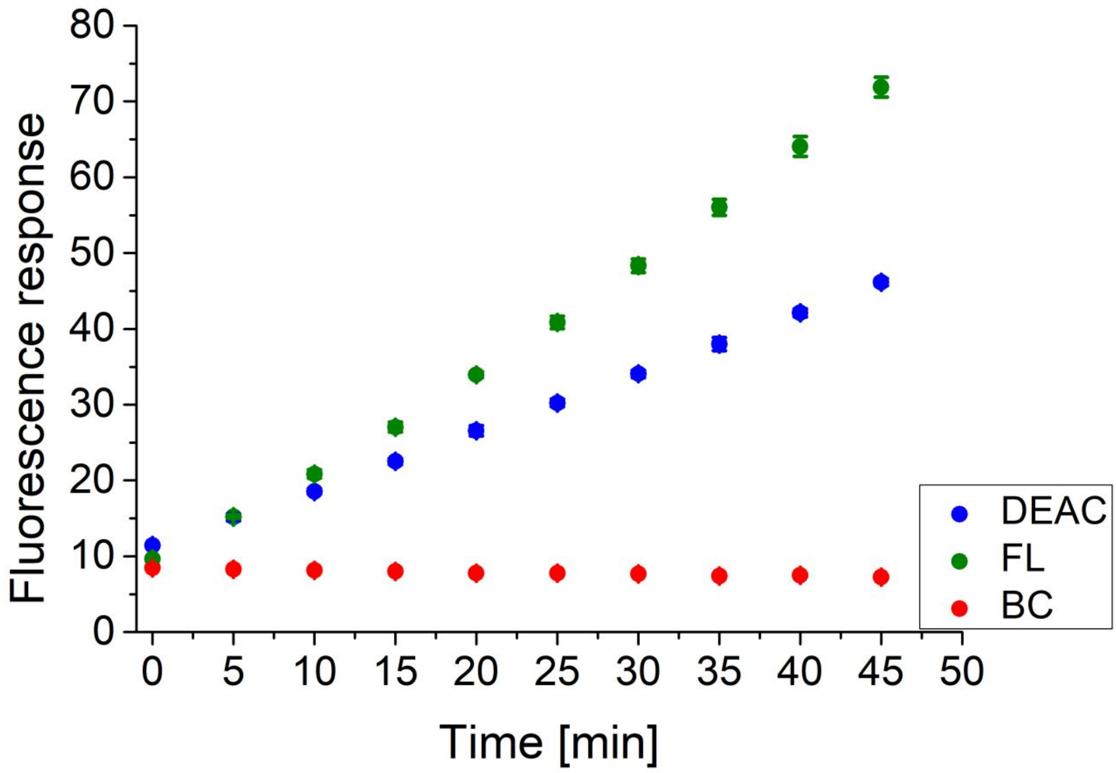

Preparation: 10 µM CP probe in DMSO (10 µL) and Tris buffer (970 µL) (0 min), then addition of ultrapure water (10 µL) with H_2O_2 (20 mM) and 1 mM HCl (10 µL) with chymotrypsin (500 ng) (0–45 min); Incubation: T=37 °C; Excitation: λ=425 nm; Emissions: λ_{DEAC}=477 nm, λ_{FL}=529 nm, λ_{BC}=722 nm; $Slit_{EXC}/Slit_{EMS}$=10/10 nm. All measurements were performed in three parallels. The corresponding average values and standard deviations are reported.

Table S25. Time-dependent fluorescence response of CP probe – detection of chymotrypsin (c=1 µg/mL) and hydrogen peroxide (c=20 µM). The visualization of the corresponding numerical data can be found in the graph under the table.

Time [min]	DEAC Average	DEAC St. Dev.	FL Average	FL St. Dev.	BC Average	BC St. Dev.
0	11.33	0.32	9.15	0.30	8.58	0.29
5	17.46	0.48	11.94	0.32	8.65	0.30
10	23.11	0.46	14.29	0.25	8.60	0.22
15	28.30	0.27	16.74	0.39	8.33	0.33
20	33.79	0.56	19.23	0.24	8.22	0.31
25	39.20	0.28	21.37	0.31	8.28	0.33
30	44.49	0.21	24.10	0.42	8.04	0.27
35	50.06	0.60	26.43	0.24	8.21	0.34
40	55.03	0.72	28.96	0.31	8.09	0.36
45	60.33	0.71	31.70	0.55	8.20	0.19

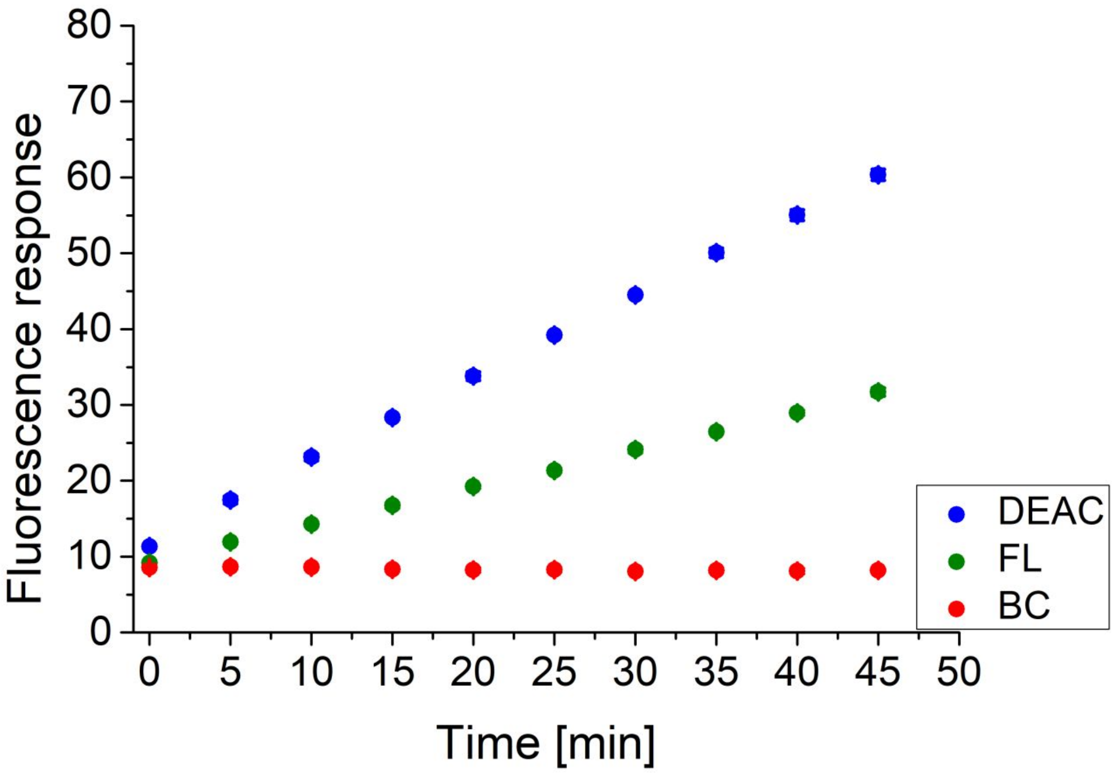

Preparation: 10 µM CP probe in DMSO (10 µL) and Tris buffer (970 µL) (0 min), then addition of ultrapure water (10 µL) with H_2O_2 (2 mM) and 1 mM HCl (10 µL) with chymotrypsin (1 µg) (0–45 min); Incubation: T=37 °C; Excitation: λ=425 nm; Emissions: λ_{DEAC}=477 nm, λ_{FL}=529 nm, λ_{BC}=722 nm; $Slit_{EXC}$/$Slit_{EMS}$=10/10 nm. All measurements were performed in three parallels. The corresponding average values and standard deviations are reported.

Table S26. Time-dependent fluorescence response of CP probe – detection of chymotrypsin (c=1 µg/mL) and hydrogen peroxide (c=50 µM). The visualization of the corresponding numerical data can be found in the graph under the table.

Time [min]	DEAC Average	DEAC St. Dev.	FL Average	FL St. Dev.	BC Average	BC St. Dev.
0	11.62	0.34	9.68	0.26	8.88	0.21
5	17.56	0.61	13.02	0.26	8.73	0.07
10	23.18	0.80	15.91	0.26	8.61	0.10
15	28.89	0.98	19.50	0.39	8.55	0.09
20	34.64	1.30	22.63	0.40	8.52	0.06
25	40.43	1.82	26.18	0.77	8.50	0.09
30	46.15	2.38	29.98	1.18	8.33	0.07
35	51.83	2.62	33.33	1.06	8.19	0.11
40	57.67	3.05	36.98	1.37	8.20	0.14
45	62.56	2.95	40.43	1.23	8.15	0.14

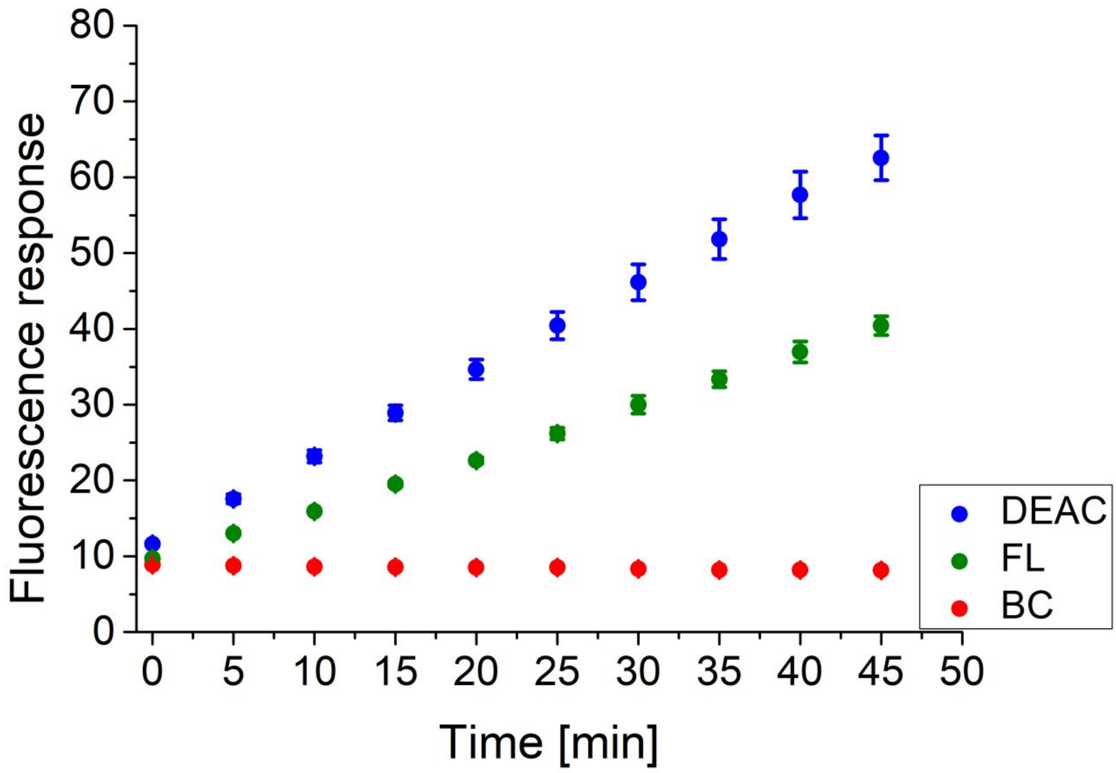

Preparation: 10 µM CP probe in DMSO (10 µL) and Tris buffer (970 µL) (0 min), then addition of ultrapure water (10 µL) with H_2O_2 (5 mM) and 1 mM HCl (10 µL) with chymotrypsin (1 µg) (0–45 min); Incubation: T=37 °C; Excitation: λ=425 nm; Emissions: λ_{DEAC}=477 nm, λ_{FL}=529 nm, λ_{BC}=722 nm; $Slit_{EXC}/Slit_{EMS}$=10/10 nm. All measurements were performed in three parallels. The corresponding average values and standard deviations are reported.

Table S27. Time-dependent fluorescence response of CP probe – detection of chymotrypsin (c=1 µg/mL) and hydrogen peroxide (c=100 µM). The visualization of the corresponding numerical data can be found in the graph under the table.

Time [min]	DEAC Average	DEAC St. Dev.	FL Average	FL St. Dev.	BC Average	BC St. Dev.
0	11.29	0.20	9.89	0.32	8.37	0.12
5	17.21	0.42	13.77	0.57	8.32	0.16
10	22.67	0.78	17.87	0.95	8.13	0.27
15	28.47	1.24	21.96	1.82	8.15	0.21
20	34.13	1.35	26.96	1.54	7.98	0.04
25	40.41	1.53	32.39	1.75	7.97	0.10
30	45.53	1.97	37.00	2.03	7.83	0.18
35	51.84	2.33	42.17	2.11	7.76	0.15
40	57.13	2.01	47.65	2.23	7.57	0.11
45	63.14	2.18	52.72	2.36	7.59	0.24

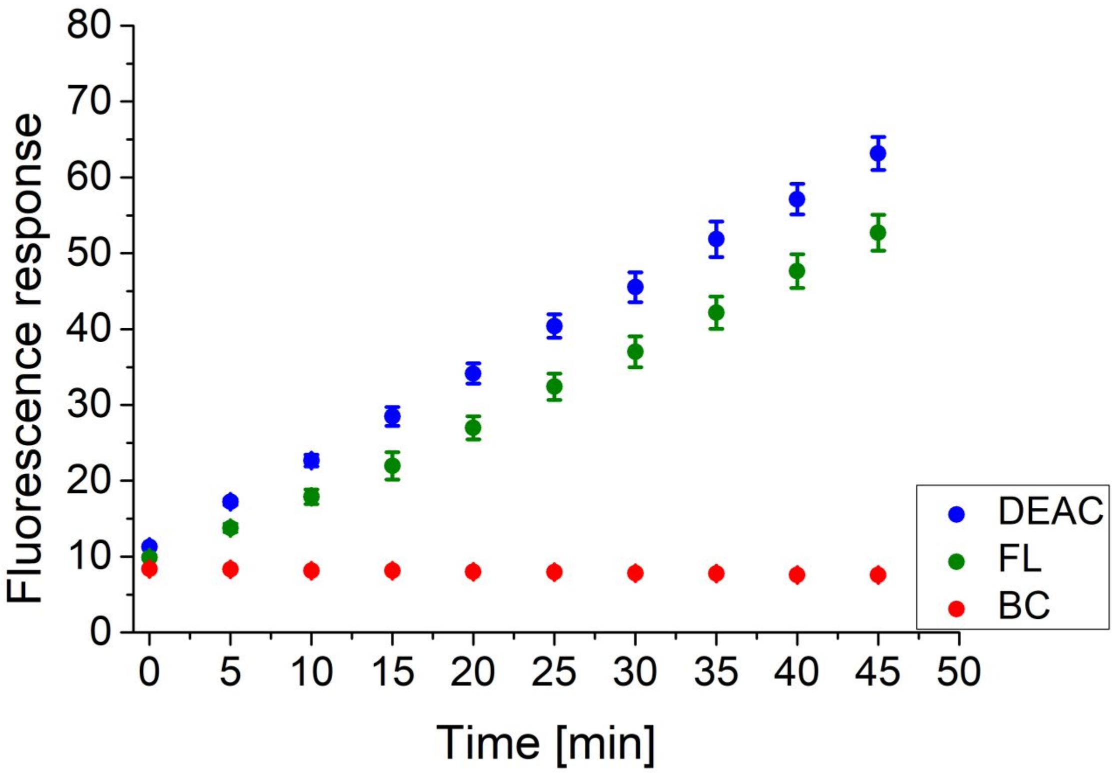

Preparation: 10 µM CP probe in DMSO (10 µL) and Tris buffer (970 µL) (0 min), then addition of ultrapure water (10 µL) with H_2O_2 (10 mM) and 1 mM HCl (10 µL) with chymotrypsin (1 µg) (0–45 min); Incubation: T=37 °C; Excitation: λ=425 nm; Emissions: λ_{DEAC}=477 nm, λ_{FL}=529 nm, λ_{BC}=722 nm; $Slit_{EXC}/Slit_{EMS}$=10/10 nm. All measurements were performed in three parallels. The corresponding average values and standard deviations are reported.

Table S28. Time-dependent fluorescence response of CP probe – detection of chymotrypsin (c=1 µg/mL) and hydrogen peroxide (c=200 µM). The visualization of the corresponding numerical data can be found in the graph under the table.

Time [min]	DEAC Average	DEAC St. Dev.	FL Average	FL St. Dev.	BC Average	BC St. Dev.
0	10.94	0.37	9.28	0.20	8.28	0.30
5	17.46	0.59	15.46	0.32	8.09	0.21
10	23.12	0.67	21.50	0.36	7.81	0.24
15	29.34	1.12	28.57	0.68	7.73	0.19
20	35.71	1.03	35.98	0.96	7.59	0.22
25	41.82	1.55	43.73	1.16	7.46	0.25
30	47.47	1.48	51.79	0.83	7.30	0.18
35	54.43	1.74	60.86	1.64	7.31	0.06
40	59.96	2.32	69.15	2.24	7.18	0.14
45	66.62	2.33	77.47	1.94	6.98	0.25

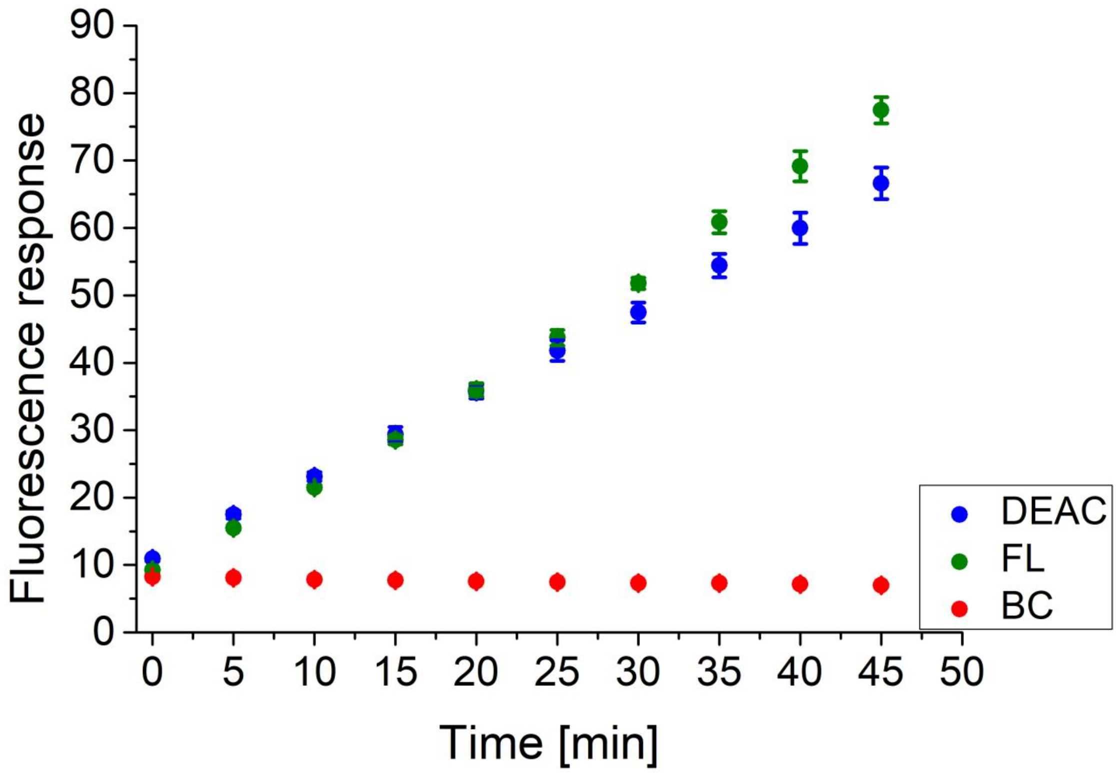

Preparation: 10 µM CP probe in DMSO (10 µL) and Tris buffer (970 µL) (0 min), then addition of ultrapure water (10 µL) with H_2O_2 (20 mM) and 1 mM HCl (10 µL) with chymotrypsin (1 µg) (0–45 min); Incubation: T=37 °C; Excitation: λ=425 nm; Emissions: λ_{DEAC}=477 nm, λ_{FL}=529 nm, λ_{BC}=722 nm; $Slit_{EXC}/Slit_{EMS}$=10/10 nm. All measurements were performed in three parallels. The corresponding average values and standard deviations are reported.

Table S29. Fluorescence response of CP probe after 45 min of incubation at 37 °C in the absence/presence of chymotrypsin and hydrogen peroxide in different concentration.

Protease [ng/mL]	H_2O_2 [µM]	DEAC Average	DEAC St. Dev.	FL Average	FL St. Dev.	DEAC/FL Average	DEAC/FL St. Dev.
0	0	12.10	0.29	12.04	0.43	1.00	0.04
50	0	14.99	0.30	12.89	0.53	1.16	0.05
100	0	18.74	0.82	15.47	0.05	1.21	0.05
200	0	21.90	0.74	15.23	0.39	1.44	0.06
500	0	34.67	1.30	19.15	0.93	1.81	0.11
1000	0	57.02	2.26	26.21	1.05	2.18	0.12
0	10	13.10	0.06	14.43	0.21	0.91	0.01
0	20	13.81	0.24	17.27	0.21	0.80	0.02
0	50	14.47	0.41	24.58	0.90	0.59	0.03
0	100	16.37	0.32	37.82	1.12	0.43	0.02
0	200	20.11	0.43	62.40	0.97	0.32	0.01
100	20	17.72	0.32	18.27	0.14	0.97	0.02
100	50	18.71	0.11	25.57	0.73	0.73	0.02
100	100	20.71	0.61	38.43	1.44	0.54	0.03
100	200	24.72	0.63	61.62	2.45	0.40	0.02
200	20	23.08	0.73	20.16	0.15	1.14	0.04
200	50	24.57	0.60	26.91	0.50	0.91	0.03
200	100	26.05	0.53	40.14	0.95	0.65	0.02
200	200	30.18	0.46	67.27	1.49	0.45	0.01
500	20	36.94	0.80	24.18	0.66	1.53	0.05
500	50	38.96	1.30	32.49	0.90	1.20	0.05
500	100	40.14	1.07	45.11	0.51	0.89	0.03
500	200	46.16	0.47	71.88	1.29	0.64	0.01
1000	20	60.33	0.71	31.70	0.55	1.90	0.04
1000	50	62.56	2.95	40.43	1.23	1.55	0.09
1000	100	63.14	2.18	52.72	2.36	1.20	0.07
1000	200	66.62	2.33	77.47	1.94	0.86	0.04

Preparation: 10 µM CP probe in DMSO (10 µL) and Tris buffer (970 µL), then addition of ultrapure water (10 µL) with H_2O_2 (1–20 mM) and 1 mM HCl (10 µL) with chymotrypsin (50–1000 ng); Incubation: T=37 °C; Excitation: λ=425 nm; Emissions: λ_{DEAC}=477 nm, λ_{FL}=529 nm; $Slit_{EXC}/Slit_{EMS}$=10/10 nm. All measurements were performed in three parallels. The corresponding average values and standard deviations are reported.

Table S30. Time-dependent fluorescence response of CP probe – blank sample. The visualization of the corresponding numerical data can be found in the graph under the table.

Time [min]	DEAC Average	DEAC St. Dev.	FL Average	FL St. Dev.	BC Average	BC St. Dev.
0	-	-	36.11	0.93	10.42	0.12
5	-	-	37.40	0.74	10.13	0.10
10	-	-	37.79	0.79	10.18	0.13
15	-	-	38.17	0.33	10.20	0.18
20	-	-	38.84	0.72	10.27	0.22
25	-	-	39.46	0.97	9.98	0.13
30	-	-	39.74	0.92	10.21	0.26
35	-	-	40.76	1.00	10.20	0.25
40	-	-	40.74	0.94	9.90	0.09
45	-	-	41.39	0.78	10.22	0.32

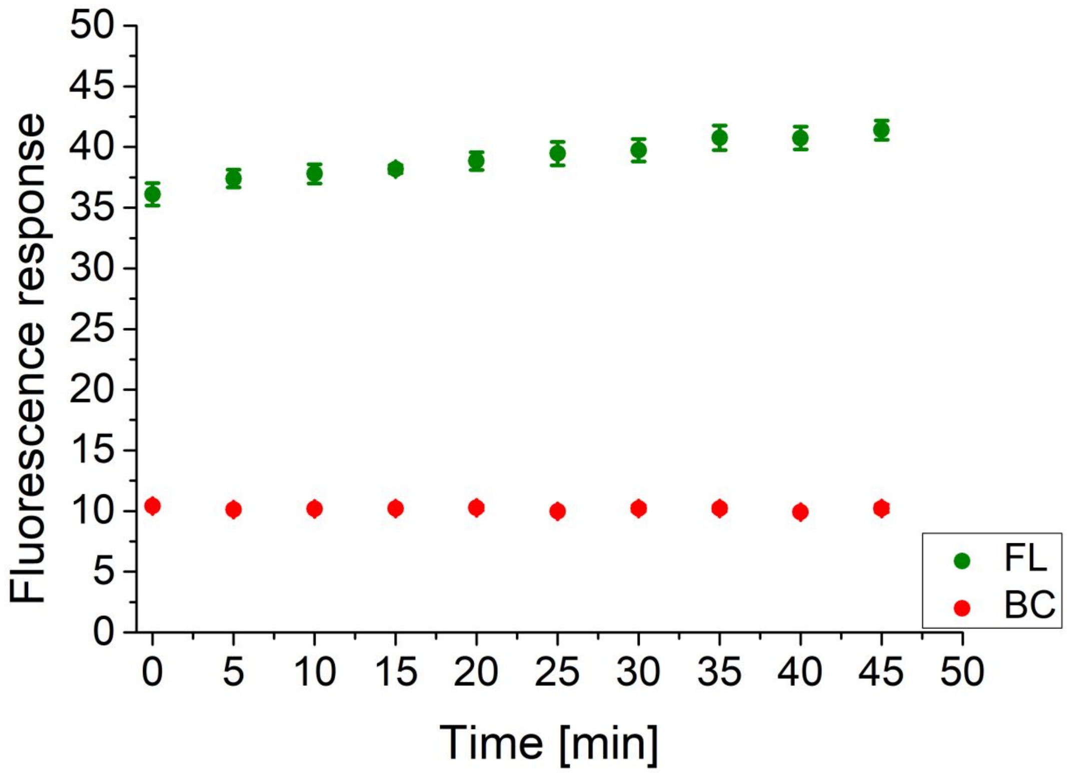

Preparation: 10 µM CP probe in DMSO (10 µL) and Tris buffer (970 µL) (0 min), then addition of ultrapure water (10 µL) and 1 mM HCl (10 µL) (0–45 min); Incubation: T=37 °C; Excitation: λ=500 nm; Emissions: λ_{FL}=529 nm, λ_{BC}=722 nm; $Slit_{EXC}/Slit_{EMS}$=10/10 nm. All measurements were performed in three parallels. The corresponding average values and standard deviations are reported.

Table S31. Time-dependent fluorescence response of CP probe – detection of chymotrypsin (c=1 µg/mL). The visualization of the corresponding numerical data can be found in the graph under the table.

Time [min]	DEAC Average	DEAC St. Dev.	FL Average	FL St. Dev.	BC Average	BC St. Dev.
0	-	-	33.81	0.32	10.07	0.27
5	-	-	35.17	0.73	10.25	0.06
10	-	-	36.31	0.68	10.49	0.11
15	-	-	37.45	0.75	10.66	0.30
20	-	-	38.10	0.36	10.87	0.14
25	-	-	39.43	0.54	10.75	0.20
30	-	-	40.19	0.44	10.92	0.15
35	-	-	41.00	0.66	11.00	0.15
40	-	-	42.29	0.77	11.27	0.13
45	-	-	43.62	0.70	11.41	0.32

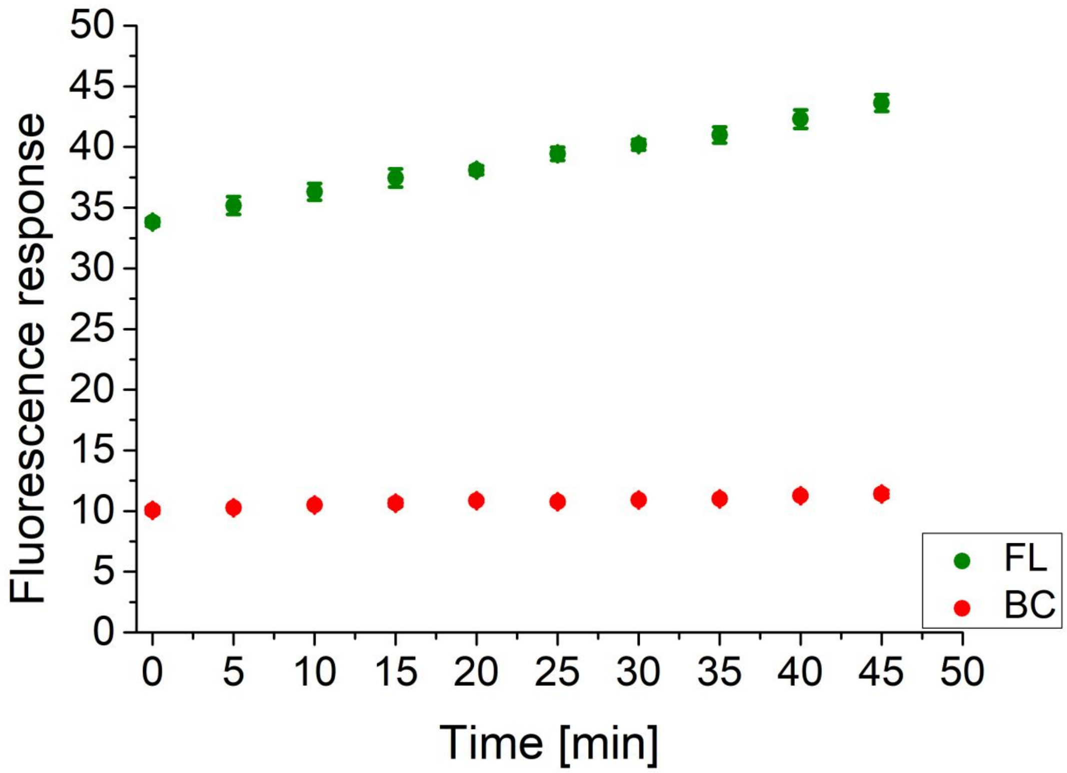

Preparation: 10 µM CP probe in DMSO (10 µL) and Tris buffer (970 µL) (0 min), then addition of ultrapure water (10 µL) and 1 mM HCl (10 µL) with chymotrypsin (1 µg) (0–45 min); Incubation: T=37 °C; Excitation: λ=500 nm; Emissions: λ_{FL}=529 nm, λ_{BC}=722 nm; $Slit_{EXC}$/$Slit_{EMS}$=10/10 nm. All measurements were performed in three parallels. The corresponding average values and standard deviations are reported.

Table S32. Time-dependent fluorescence response of CP probe – detection of hydrogen peroxide (c=200 µM). The visualization of the corresponding numerical data can be found in the graph under the table.

Time [min]	DEAC Average	DEAC St. Dev.	FL Average	FL St. Dev.	BC Average	BC St. Dev.
0	-	-	34.49	0.70	9.95	0.08
5	-	-	47.55	0.97	9.79	0.22
10	-	-	61.20	0.58	9.84	0.23
15	-	-	76.40	0.99	9.78	0.13
20	-	-	91.44	0.70	9.70	0.20
25	-	-	107.38	1.69	9.56	0.07
30	-	-	124.41	1.51	9.59	0.02
35	-	-	142.08	1.69	9.37	0.20
40	-	-	160.03	1.90	9.38	0.09
45	-	-	177.95	2.09	9.25	0.13

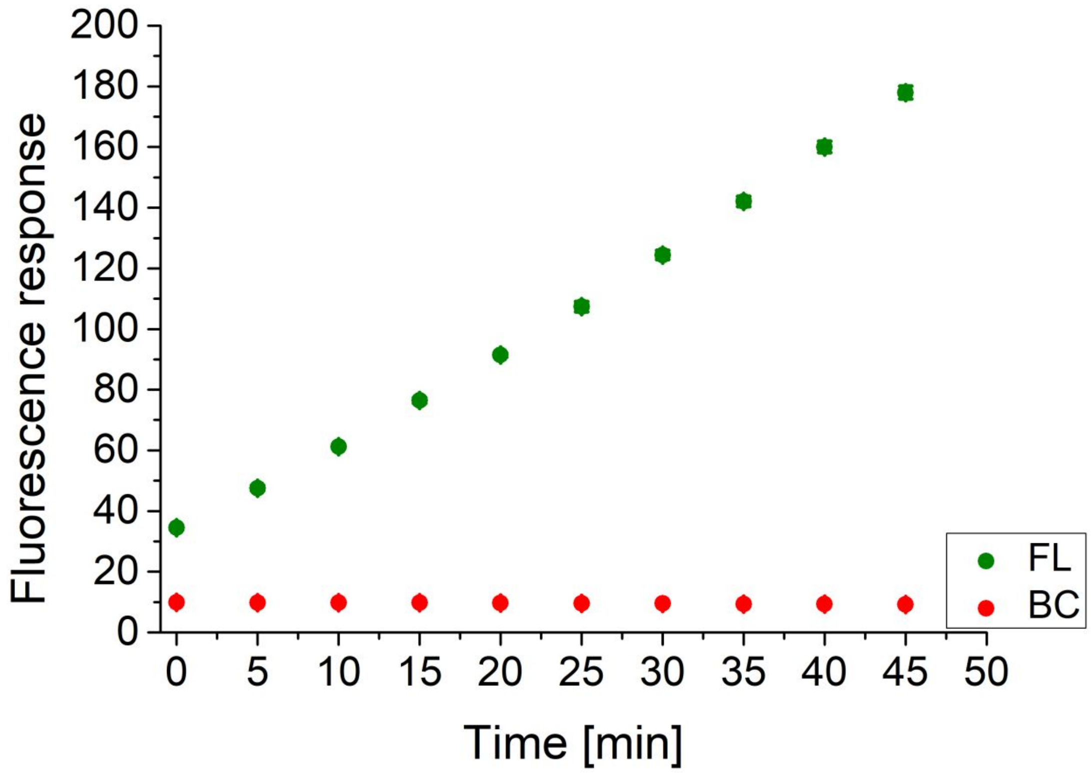

Preparation: 10 µM CP probe in DMSO (10 µL) and Tris buffer (970 µL) (0 min), then addition of ultrapure water (10 µL) with H_2O_2 (20 mM) and 1 mM HCl (10 µL) (0–45 min); Incubation: T=37 °C; Excitation: λ=500 nm; Emissions: λ_{FL}=529 nm, λ_{BC}=722 nm; $Slit_{EXC}/Slit_{EMS}$=10/10 nm. All measurements were performed in three parallels. The corresponding average values and standard deviations are reported.

Table S33. Time-dependent fluorescence response of CP probe – detection of hydrogen peroxide (c=200 µM). The visualization of the corresponding numerical data can be found in the graph under the table.

Time [min]	DEAC Average	DEAC St. Dev.	FL Average	FL St. Dev.	BC Average	BC St. Dev.
0	-	-	-	-	50.41	1.47
5	-	-	-	-	49.90	0.73
10	-	-	-	-	50.76	1.74
15	-	-	-	-	50.11	0.33
20	-	-	-	-	49.79	1.00
25	-	-	-	-	51.10	2.11
30	-	-	-	-	51.46	0.35
35	-	-	-	-	51.54	1.16
40	-	-	-	-	50.94	2.32
45	-	-	-	-	51.06	0.48

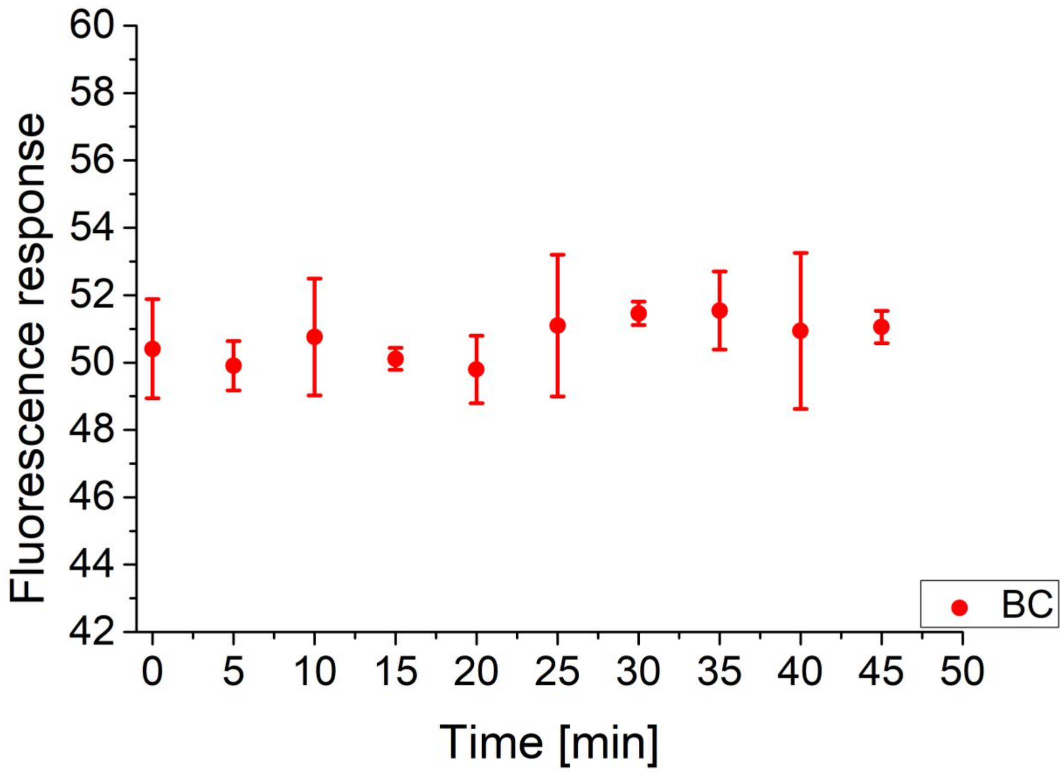

Preparation: 10 µM CP probe in DMSO (10 µL) and Tris buffer (970 µL) (0 min), then addition of ultrapure water (10 µL) with H_2O_2 (20 mM) and 1 mM HCl (10 µL) (0–45 min); Incubation: T=37 °C; Excitation: λ=680 nm; Emission: λ_{BC}=722 nm; $Slit_{EXC}/Slit_{EMS}$=10/10 nm. All measurements were performed in three parallels. The corresponding average values and standard deviations are reported.

Table S34. Time-dependent fluorescence response of C probe – blank sample. The visualization of the corresponding numerical data can be found in the graph under the table.

Time [min]	DEAC Average	DEAC St. Dev.	FL Average	FL St. Dev.	BC Average	BC St. Dev.
0	6.66	0.36	210.69	1.64	-	-
5	6.70	0.22	210.67	2.31	-	-
10	6.68	0.27	209.59	1.00	-	-
15	6.53	0.37	209.39	1.45	-	-
20	6.54	0.37	209.58	2.19	-	-
25	6.49	0.33	209.17	0.75	-	-
30	6.57	0.21	208.98	1.95	-	-
35	6.48	0.35	207.96	1.27	-	-
40	6.53	0.27	209.20	1.60	-	-
45	6.43	0.09	208.94	1.58	-	-

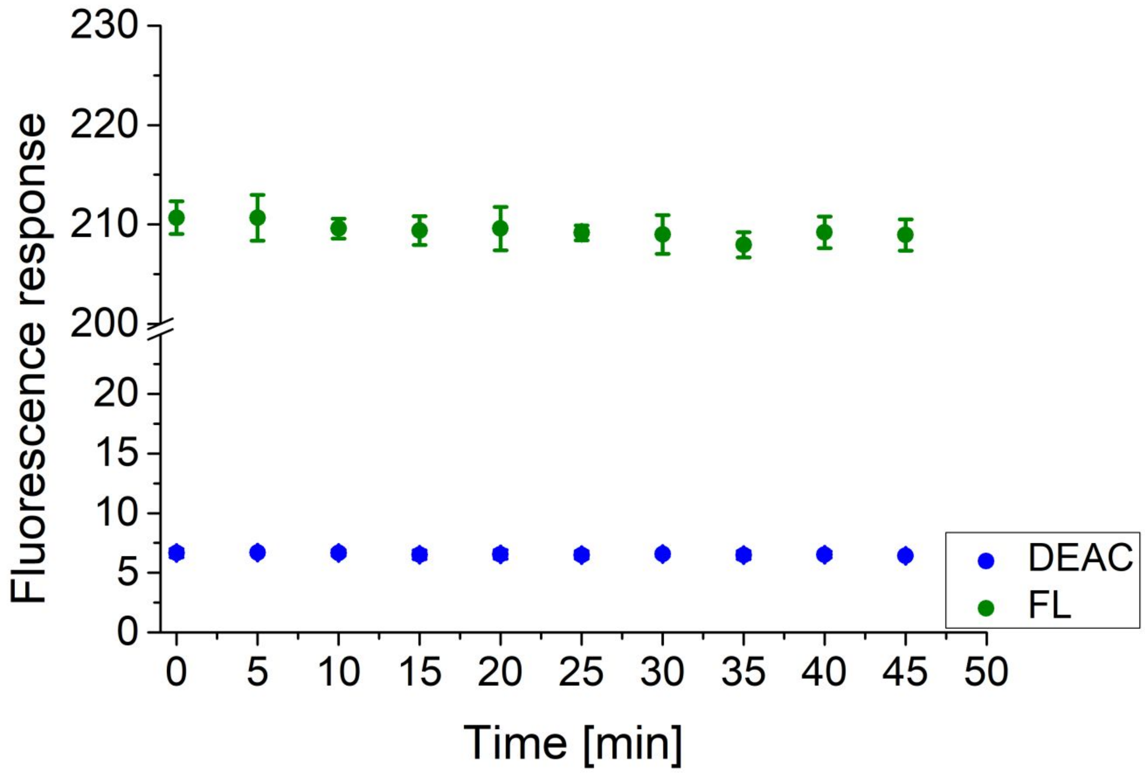

Preparation: 10 µM C probe in DMSO (10 µL) and Tris buffer (970 µL) (0 min), then addition of ultrapure water (10 µL) and 1 mM HCl (10 µL) (0–45 min); Incubation: T=37 °C; Excitation: λ=425 nm; Emissions: λ_{DEAC}=477 nm, λ_{FL}=529 nm; $Slit_{EXC}/Slit_{EMS}$=5/5 nm. All measurements were performed in three parallels. The corresponding average values and standard deviations are reported.

Table S35. Time-dependent fluorescence response of C probe – detection of chymotrypsin (c=10 µg/mL). The visualization of the corresponding numerical data can be found in the graph under the table.

Time [min]	DEAC Average	DEAC St. Dev.	FL Average	FL St. Dev.	BC Average	BC St. Dev.
0	6.25	0.10	211.82	1.33	-	-
5	9.05	0.18	210.15	2.61	-	-
10	12.00	0.23	210.30	1.87	-	-
15	14.68	0.10	211.15	2.27	-	-
20	17.25	0.33	210.93	2.20	-	-
25	19.46	0.34	211.65	1.72	-	-
30	21.71	0.07	211.15	1.56	-	-
35	23.87	0.17	210.93	2.71	-	-
40	25.60	0.37	212.25	2.52	-	-
45	27.60	0.25	211.18	3.05	-	-

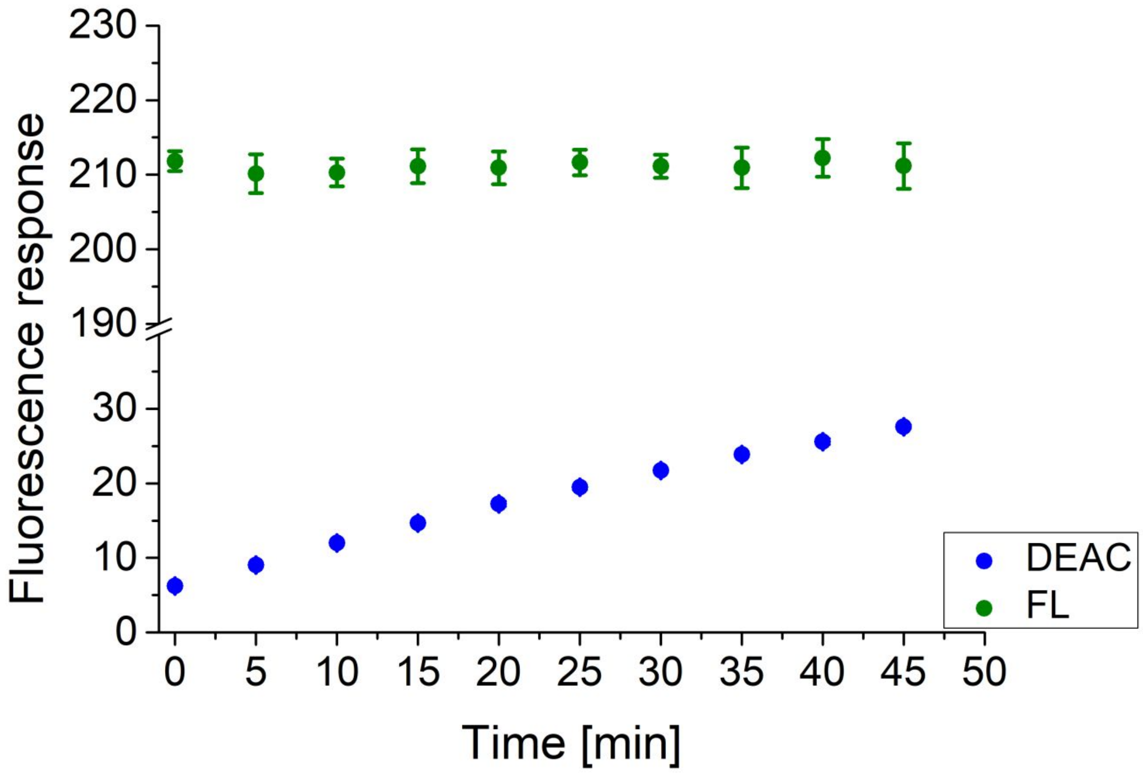

Preparation: 10 µM C probe in DMSO (10 µL) and Tris buffer (970 µL) (0 min), then addition of ultrapure water (10 µL) and 1 mM HCl (10 µL) with chymotrypsin (10 µg) (0–45 min); Incubation: T=37 °C; Excitation: λ=425 nm; Emissions: λ_{DEAC}=477 nm, λ_{FL}=529 nm; $Slit_{EXC}/Slit_{EMS}$=5/5 nm. All measurements were performed in three parallels. The corresponding average values and standard deviations are reported.

Table S36. Time-dependent fluorescence response of C probe – detection of hydrogen peroxide (c=2 mM). The visualization of the corresponding numerical data can be found in the graph under the table.

Time [min]	DEAC Average	DEAC St. Dev.	FL Average	FL St. Dev.	BC Average	BC St. Dev.
0	6.30	0.06	209.77	4.57	-	-
5	6.17	0.17	209.90	4.71	-	-
10	6.32	0.19	209.40	3.92	-	-
15	6.23	0.19	209.35	4.37	-	-
20	6.24	0.35	209.94	5.40	-	-
25	6.08	0.25	209.08	3.34	-	-
30	6.30	0.17	210.22	5.39	-	-
35	6.24	0.15	209.93	4.81	-	-
40	6.35	0.17	210.10	4.13	-	-
45	6.42	0.27	210.48	4.46	-	-

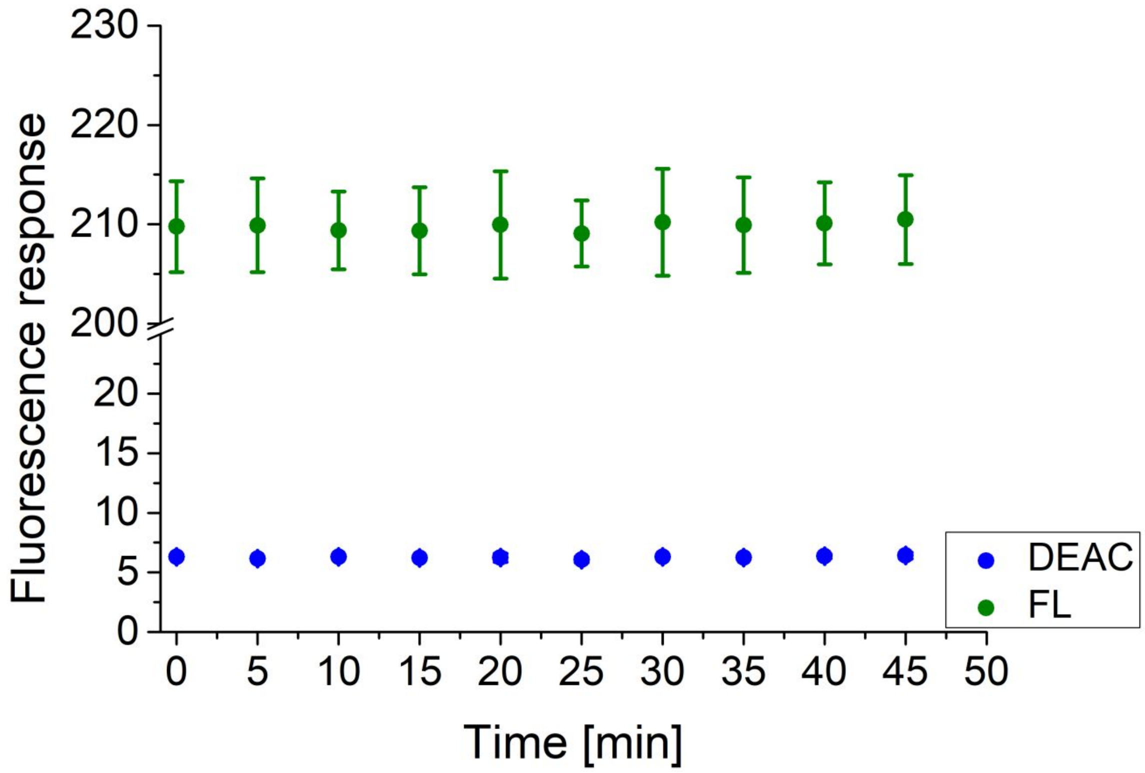

Preparation: 10 µM C probe in DMSO (10 µL) and Tris buffer (970 µL) (0 min), then addition of ultrapure water (10 µL) with H_2O_2 (200 mM) and 1 mM HCl (10 µL) (0–45 min); Incubation: T=37 °C; Excitation: λ=425 nm; Emissions: λ_{DEAC}=477 nm, λ_{FL}=529 nm; $Slit_{EXC}$/$Slit_{EMS}$=5/5 nm. All measurements were performed in three parallels. The corresponding average values and standard deviations are reported.

Table S37. Time-dependent fluorescence response of C probe – detection of chymotrypsin (10 µg/mL) and hydrogen peroxide (c=2 mM). The visualization of the corresponding numerical data can be found in the graph under the table.

Time [min]	DEAC Average	DEAC St. Dev.	FL Average	FL St. Dev.	BC Average	BC St. Dev.
0	6.48	0.03	214.48	2.33	-	-
5	9.50	0.15	213.22	1.38	-	-
10	12.62	0.47	212.02	1.84	-	-
15	15.37	0.40	213.23	1.57	-	-
20	18.01	0.33	212.67	1.75	-	-
25	20.90	0.37	212.79	2.16	-	-
30	22.93	0.43	212.58	2.85	-	-
35	25.00	0.93	213.23	2.37	-	-
40	27.53	0.52	213.60	2.61	-	-
45	29.53	0.56	212.93	2.23	-	-

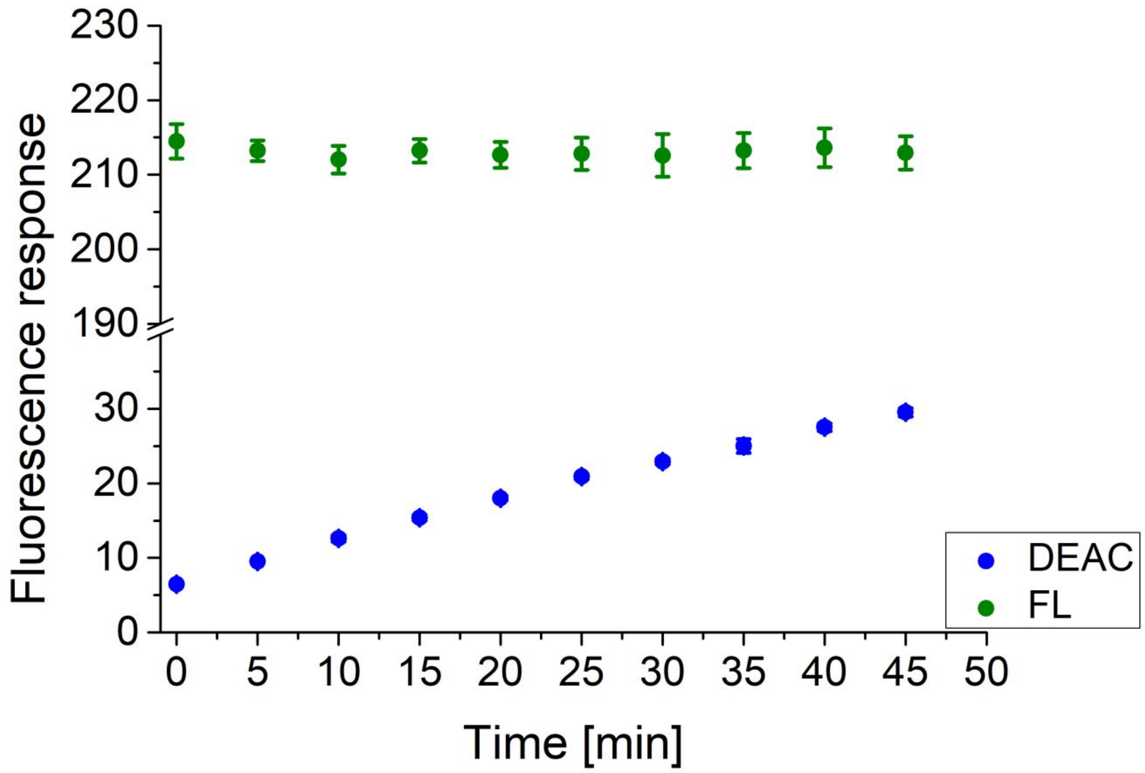

Preparation: 10 µM C probe in DMSO (10 µL) and Tris buffer (970 µL) (0 min), then addition of ultrapure water (10 µL) with H_2O_2 (200 mM) and 1 mM HCl (10 µL) with chymotrypsin (10 µg) (0–45 min); Incubation: T=37 °C; Excitation: λ=425 nm; Emissions: λ_{DEAC}=477 nm, λ_{FL}=529 nm; $Slit_{EXC}/Slit_{EMS}$=5/5 nm. All measurements were performed in three parallels. The corresponding average values and standard deviations are reported.

Figure S18. Normalized fluorescence excitation ($\lambda_{MAX.}$= 410 nm) and emission ($\lambda_{MAX.}$= 471 nm) profiles of DEAC dye (10 µM) in 1% DMSO (*V*/*V*) in 0.1 M Tris buffer (pH= 8.0) at 37 °C.

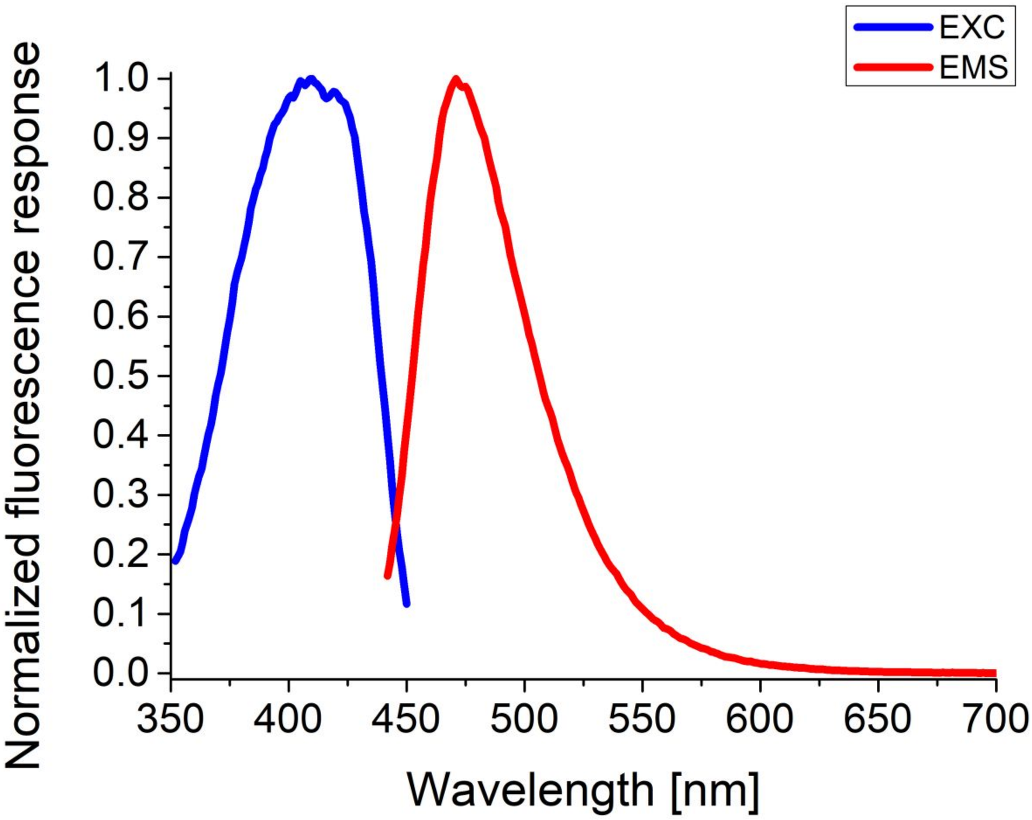

Figure S19. Normalized fluorescence excitation ($\lambda_{MAX.}$= 497 nm) and emission ($\lambda_{MAX.}$= 517 nm) profiles of FL dye (10 µM) in 1% DMSO (*V*/*V*) in 0.1 M Tris buffer (pH= 8.0) at 37 °C.

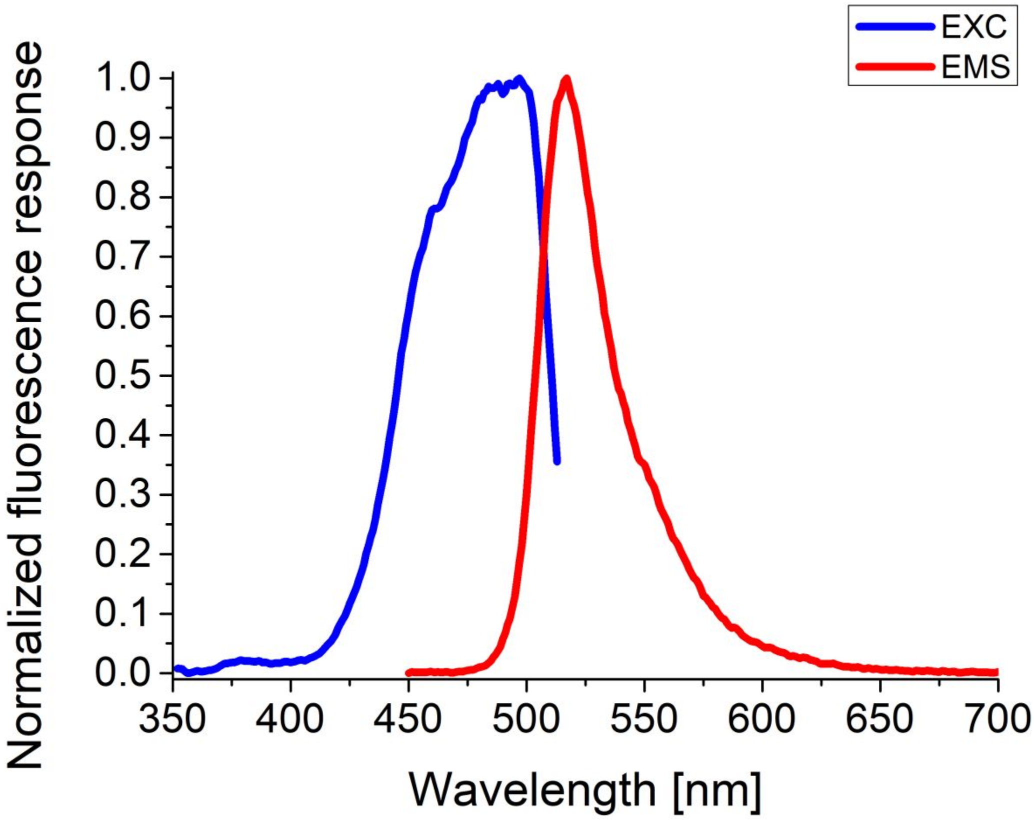

Figure S20. Normalized fluorescence excitation ($\lambda_{MAX.}$= 657 nm) and emission ($\lambda_{MAX.}$= 691 nm) profiles of BC dye (10 μM) in 1% DMSO (*V*/*V*) in 0.1 M Tris buffer (pH= 8.0) at 37 °C.

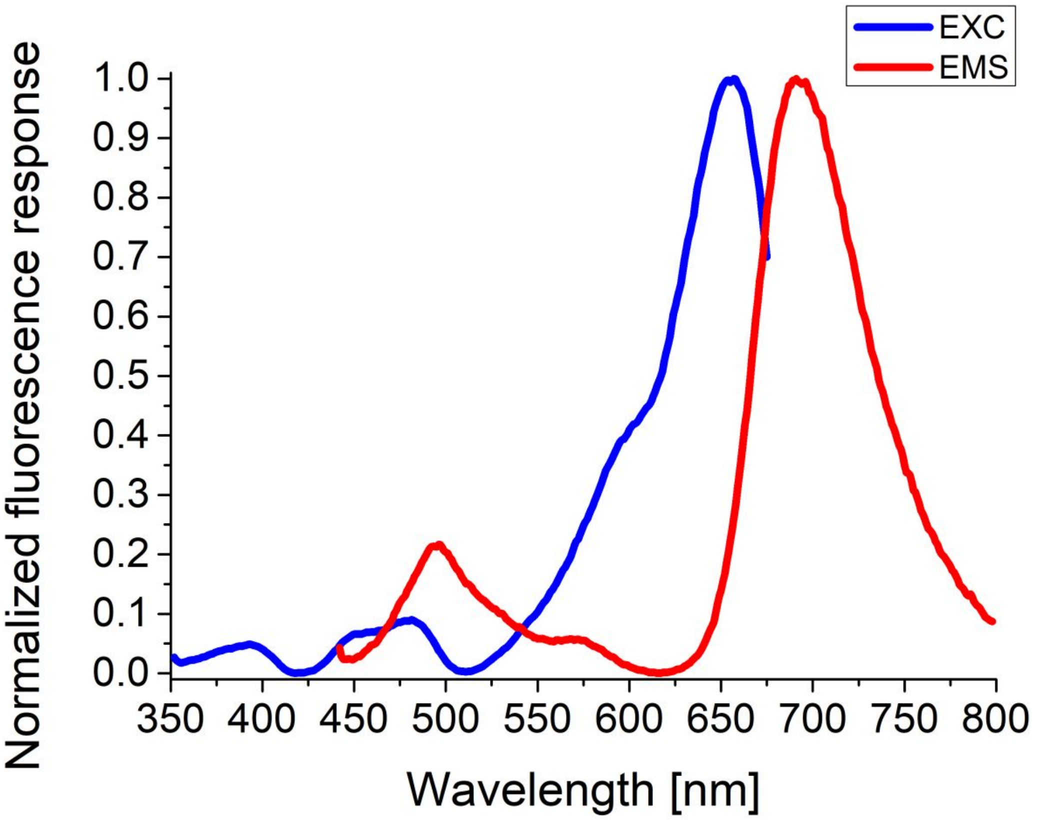

Table S38. The FL/DEAC ratio-based emission response of CP probe in the absence and presence of various reactive oxygen species (ROS). The visualization of the corresponding numerical data can be found in the graph under the table.

Entry (X-axis)	Species [100 µM]	DEAC Average	DEAC St. Dev.	FL Average	FL St. Dev.	FL/DEAC Average	FL/DEAC St. Dev.
**1**	Blank	11.70	0.03	14.30	0.73	1.22	0.06
**2**	H_2O_2	14.74	0.34	28.80	1.09	1.95	0.09
**3**	$^\bullet OH$	11.43	0.20	12.67	0.14	1.11	0.02
**4**	*t*BuOOH	11.82	0.27	13.74	1.09	1.16	0.10
**5**	$^\bullet O$*t*Bu	11.37	0.11	12.96	0.24	1.14	0.02
**6**	$O_2^{\bullet -}$	13.55	0.39	17.53	0.50	1.29	0.05
**7**	ClO^-	11.50	0.18	11.94	0.19	1.04	0.02

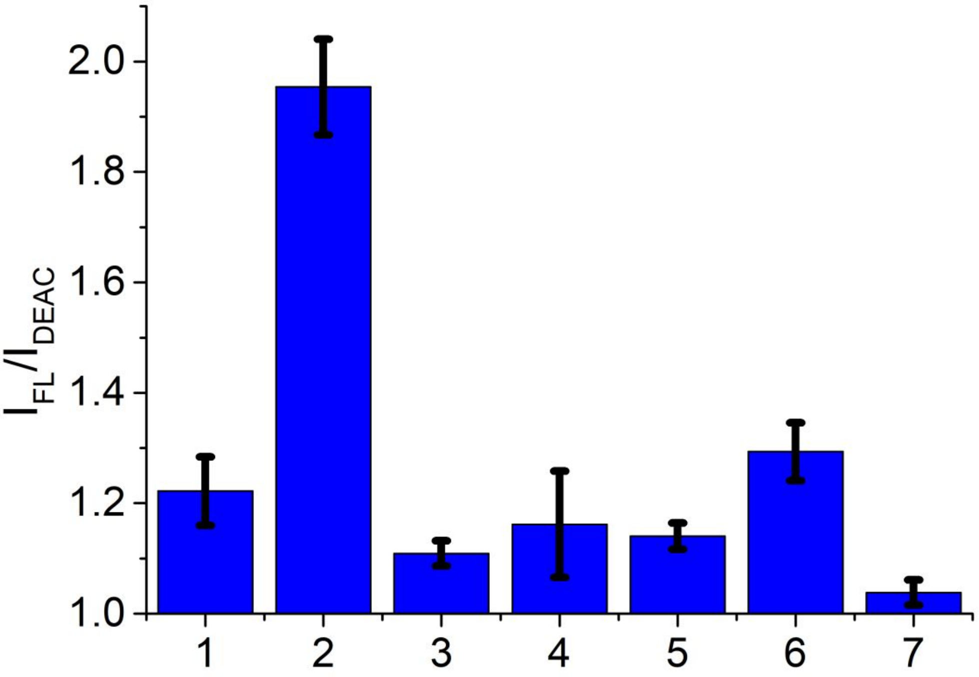

Preparation: 10 µM CP probe in DMSO (10 µL) and Tris buffer (980 µL), then addition of ultrapure water (10 µL) (1); 10 µM CP probe in DMSO (10 µL) and Tris buffer (980 µL), then addition of ultrapure water (10 µL) with H_2O_2 (10 mM) (2); 10 µM CP probe in DMSO (10 µL) and Tris buffer (980 µL), then addition of ultrapure water (10 µL) with $^\bullet OH$ (10 mM) (3); 10 µM CP probe in DMSO (10 µL) and Tris buffer (980 µL), then addition of ultrapure water (10 µL) with *t*BuOOH (10 mM) (4); 10 µM CP probe in DMSO (10 µL) and Tris buffer (980 µL), then addition of ultrapure water (10 µL) with $^\bullet O$*t*Bu (10 mM) (5); 10 µM CP probe in DMSO (2 µL) and Tris buffer (988 µL), then addition of DMSO (10 µL) with $O_2^{\bullet -}$ (10 mM) (6); 10 µM CP probe in DMSO (10 µL) and Tris buffer (980 µL), then addition of ultrapure water (10 µL) with ClO^- (10 mM) (7). Incubation: T=37 °C; Excitation: λ=425 nm; Emissions: λ_{DEAC}=477 nm, λ_{FL}=529 nm; $Slit_{EXC}/Slit_{EMS}$=10/10 nm. All measurements were performed in three parallels. The corresponding average values and standard deviations are reported.
